# Synthesis of *N*-Alkenylated Heterocycles via T_3_P-Promoted Condensation with Ketones

**DOI:** 10.1021/acs.joc.4c00803

**Published:** 2024-07-31

**Authors:** Lorenzo Jacopo Ilic Balestri, Julia Beveridge, Johan Gising, Luke R. Odell

**Affiliations:** Department of Medicinal Chemistry, Uppsala University, Box-574, SE-751 23 Uppsala, Sweden

## Abstract

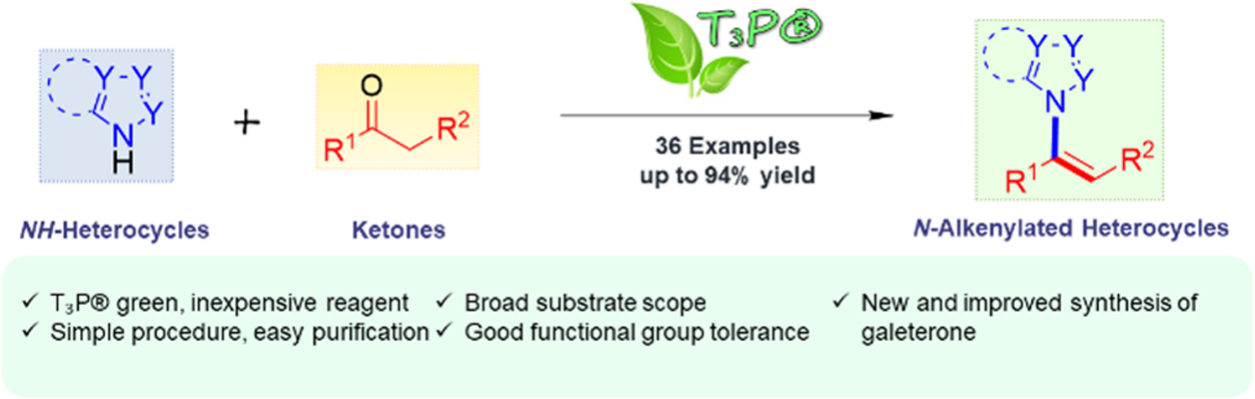

Herein, we describe a convenient protocol for the synthesis of *N*-alkenylated heterocycles using abundant ketone electrophiles and T_3_P as a water scavenger under microwave irradiation. The method can be applied to a diverse range of *NH*-heterocycles and ketones with good to excellent yields (up to 94%). This procedure is particularly attractive, as it is metal- and base-free, tolerates a variety of functional groups, and offers ease of product purification. The utility of the protocol was exemplified by synthesizing pharmaceutically relevant scaffolds containing the *N*-alkenyl motif and was further extended to a one-pot reductive amination sequence.

## Introduction

**Figure 1:**
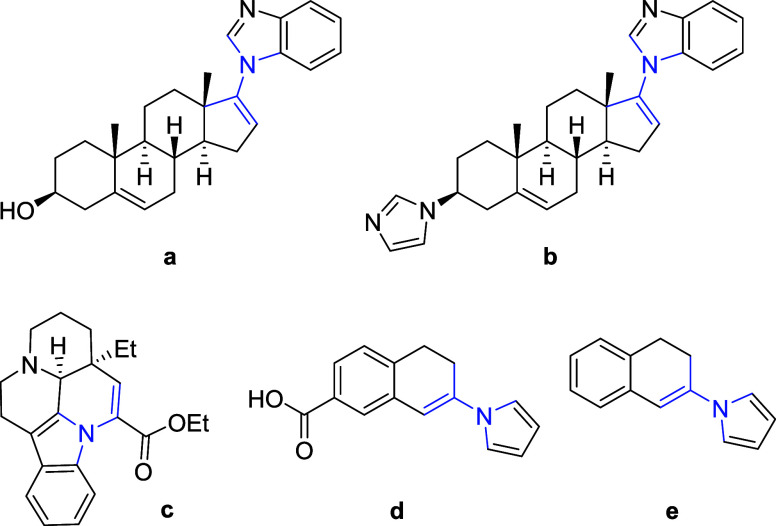
Biologically important *N*-(1-cycloalkenyl) heterocycles. Galeterone (a) and VNPP433–3β (b) are used for the treatment of prostate cancer. Vinpocetine (c) is a strong vasodilator, whereas rolafragel (d) is a potent inhibitor of thromboxane synthase. Finally, the 1-imidazole-substituted dihydronaphthalene (e) is an inhibitor of aldosterone synthase.

Nitrogen heterocycles represent the core of many natural products and are considered a “privileged structure” in medicinal chemistry due to their widespread occurrence in many approved pharmaceutical drugs.^1^ Consequently, organic chemists are continuously developing new synthetic methodologies devoted to introducing substituents or constructing these rings.^2−5^ Despite these advances, the selective *N*-functionalization of indoles^6,7^ and other *NH*-heterocycles^8−10^ is an ongoing challenge^11,12^ and the development of new and efficient methods is highly desirable.^6,13,14^*N*-Alkenyl heterocycles are an interesting and underexplored class of compounds^15^ where notable members of this family include the steroidal *N*-(1-cycloalkenyl) heterocycles galeterone^16^ (a) and VNPP433–3β^17^ (b) used for the treatment of castrate-resistant prostate cancer (Figure 1).^18^ Moreover, their utility extends beyond the anticancer field as demonstrated by vinpocetine,^19^ a derivative of the alkaloid vincamine with a strong vasodilatory effect that is used for the treatment of stroke, and rolafragel,^20^(d), a selective inhibitor of thromboxane-A synthase for the treatment of vasospasm and thrombosis. Finally, it is worth mentioning that poly(*N*-vinylindole)s have also been reported as promising semiconducting and photosensitive materials.^21,22^

Traditionally, *NH*-alkenylated heterocycles are prepared via multistep reaction sequences characterized by an initial C–N bond formation and a subsequent elimination step to install the alkenyl motif.^23−25^ More recent efforts have focused on developing one-pot approaches and include the trifluoroacetic acid (TFA)-catalyzed condensation of aldehydes and indole derivatives,^14^ potassium phosphate-promoted addition of alkynes to imidazoles,^26^ epoxide opening by *NH*-heterocycles^15^ as well as the vinylation of *NH*-heterocycles with vinyl sulfonium salts.^8,27^ Alternatively, the use of transition metals such as palladium,^6,7,10,28−30^ copper,^13,31^ and gold^32^ can facilitate C–N coupling between *NH-*heterocycles and a variety of vinylic, allylic, and alkynic substrates.^6,7,10,13,28−32^ Despite their utility, these methods suffer from a number of drawbacks including the use of strong acidic/basic conditions, advanced synthetic intermediates, or expensive transition metal catalysts. Consequently, new mild and convenient methods to construct this interesting motif are highly desired.

Propylphosphonic acid cyclic anhydride (T_3_P) is an efficient and green peptide coupling reagent that has also been employed as a water scavenger in a range of different transformations. The most attractive properties of this mild acid activation reagent include its low toxicity, broad functional group tolerance, and easy workup procedure.^33−37^

**Scheme 1:**
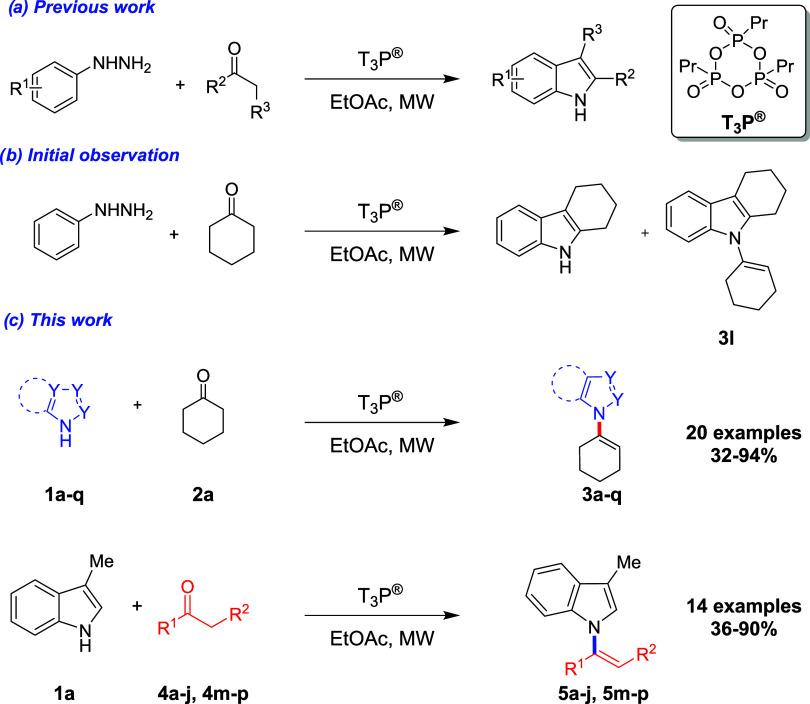
Background to Conceptualization of This Work (a) Previous work on the T_3_P-promoted indolization of arylhydrazines; (b) minor side reaction observed during method development; (c) a new one-pot, T_3_P-promoted strategy to obtain *N*-alkenylated heterocycles using ketone electrophiles.

In 2011, we described the Fischer indolization of phenylhydrazines and ketones/aldehydes using T_3_P as a dual water scavenger and acid source.^38^ During our follow-up studies, we observed formation of a recurrent minor side product when employing phenylhydrazine and an excess of cyclohexanone. Gas chromatography mass spectrometry (GCMS) and liquid chromatography-ultraviolet/MS (LC-UV/MS) analysis indicated that the product contained an additional alkenyl group and it was tentatively assigned as the *N*-alkenylated derivative **3l** (Scheme 1). Importantly, this suggested a potential new entry point into this class of compounds based on the simple condensation of an *NH*-heterocycle and a ketone. Accordingly, we explored this observation with the aim of developing a straightforward and metal-free synthesis of alkenylated *N*-heterocycles using cheap and readily available starting materials.

## Results and Discussion

The model reaction between 3-methylindole (**1a**) and cyclohexanone (**2a**) in the presence of T_3_P (50 wt % solution in EtOAc) was used as a starting point for our investigation (Table 1). Preliminary screening of the reaction conditions revealed that full conversion was reliably achieved by heating the reaction at 120 °C for 20 min using an excess of **2a** (3 equiv). Upon completion, the reaction was quenched by the addition of triethylamine (TEA) and purified via simple filtration through a silica plug to afford *N*-alkenylated product **3a** in 94% yield. The presence of T_3_P was crucial to promote the reaction as no conversion was observed (LC-UV/MS analysis) in a control experiment without the addition of T_3_P. With these conditions in hand, we further evaluated the method for its scope and general applicability. To this end, a variety of *NH*- heterocyclic compounds were reacted with cyclohexanone (**2a**), and the results are presented in Table 1.

**Table 1:**
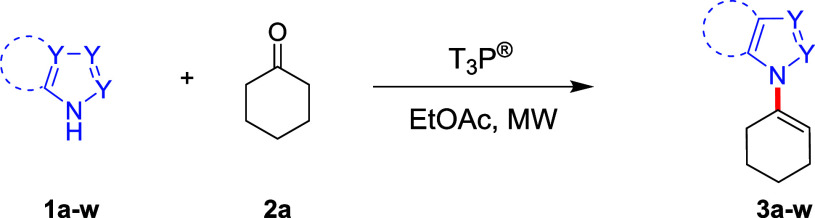

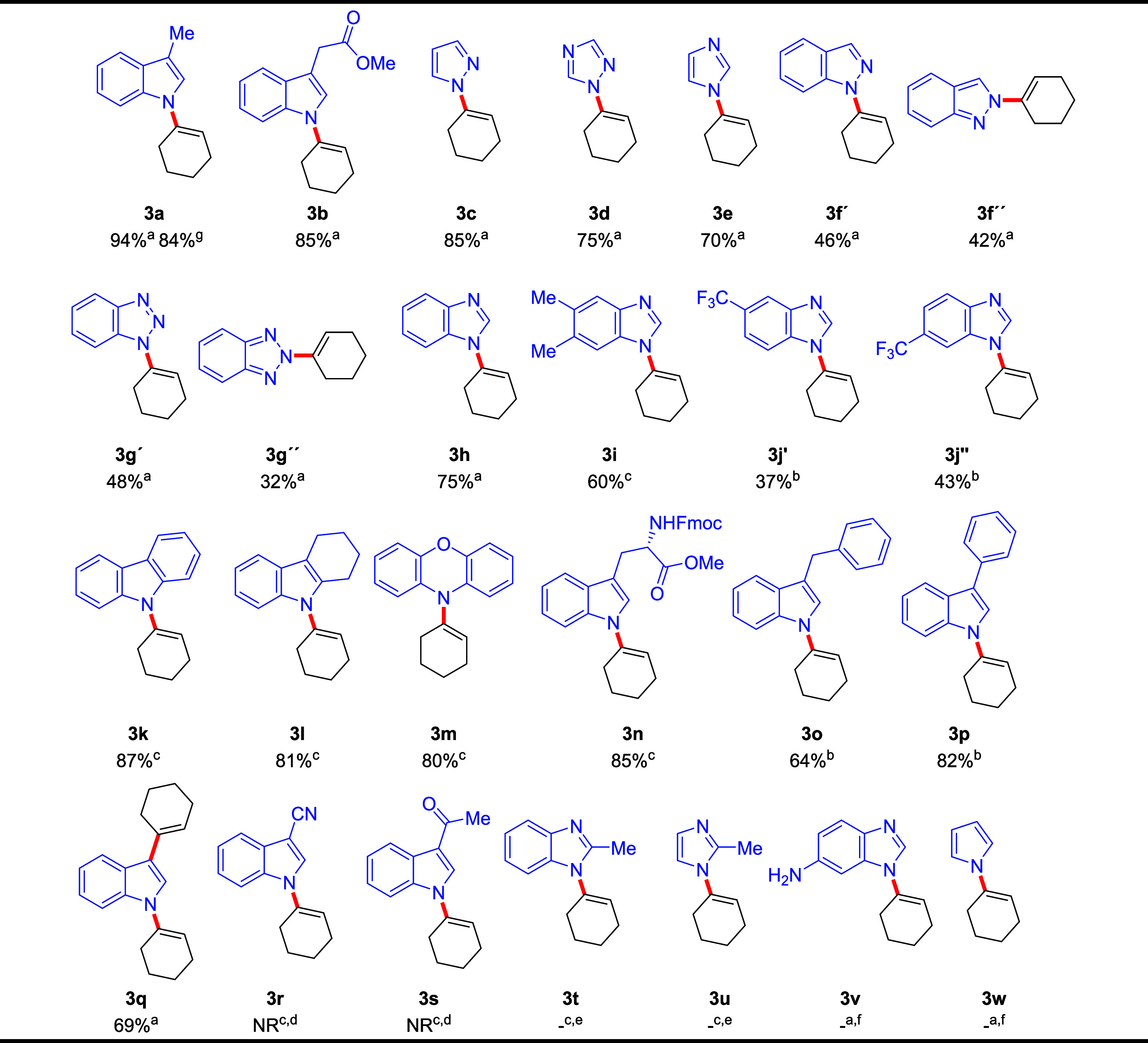
Substrate Scope Utilizing Cyclohexanone (**2a**) and Various *NH*-Heterocycles^a^

a Isolated yields. **2a** (3 equiv), T_3_P 50 wt % in EtOAc (1.5 equiv), EtOAc (0.5 mL), 120 °C, 20 min.

b 140 °C, 20 min.

c **2a** (5 equiv), 160 °C, 1 h.

d No reaction.

e Product unstable.

f Complex mixture.

g Reaction conducted on 1.5 mmol scale.

Pleasingly, the reaction worked well for a diverse array of *NH*-heterocycles including those bearing a benzo-fused ring (**1a**, **1b**, **1f**-**j**, **1n**-**q**) and five-membered heteroarenes with two or three nitrogen atoms such as pyrazole (**1c**), triazole (**1d**), and imidazole (**1e**) affording good to excellent yields (70–94%) of the desired alkenylated products (Table 1). In contrast, the tricyclic *NH*-heterocycles **1k**–**m** required higher temperatures (160 °C) and longer reaction times (up to 120 min) to reach completion, returning the products **3k**–**m** in excellent yields (80–87%). Generally, the reaction did not show any preference for the site of alkenylation, as exemplified in the case of **1f** where the *N-*1 and *N-*2 alkenylated isomers were isolated in 46 and 42% yields, respectively. Notably, the reaction tolerated a range of 3-substituents on the indole ring (**1b**, **1n**–**p**), including Fmoc-protected tryptophan that was efficiently transformed into the corresponding *N*-alkenylated amino acid **3n** in (85% yield). The reaction with indole returned a 69% yield of the C3 and N1-dialkenylated product **3q** and is in line with earlier studies on the C3-alkenylation of indoles with carbonyl compounds.^14^ The introduction of mesomerically electron-withdrawing groups at position 3 (**1r**, **1s**) was unfortunately not tolerated, and no conversion was observed even after extended heating times. This is consistent with previous reports^39^ and the reduced nucleophilicity of these substrates. Interestingly, the 2-substituted (benz)imidazoles **1t** and **1u** were efficiently converted into their alkenylated derivatives (LC- UV/MS analysis); however, the products rapidly degraded upon purification and isolation was ultimately unsuccessful. This instability may be due to reduced delocalization as the 2-substituent would force the alkenyl group to rotate out of plane to avoid unfavorable steric interactions.^40^ Unfortunately, the reaction with primary amine-substituted heterocycles such as **1v** led to the formation of complex mixtures derived from condensation of both the primary amine and NH-heterocycle groups. Finally, the reaction was successfully carried out on a 1.5 mmol scale, producing compound **3a** in 84% yield.

To further extend the scope of this methodology, a set of ketones including cyclic, acyclic branched, and unbranched substrates were investigated (Table 2). Cyclic ketones were found to be highly reactive (**4a**) and decoration with different functional groups (**4f**–**j**) was well-tolerated leading good to excellent yields of the alkenylated 3-methylindoles (**5a**,**f**-**j**). By testing the reactivity of acyclic ketones, we observed some interesting findings. For example, the reaction with acetone (**4l**) the simplest and smallest ketone led to a complex reaction mixture, whereas in the case of butanone (**4b**), or the symmetrical 3-pentanone (**4c**), the reaction proceeded smoothly, furnishing the corresponding products in 78 and 90% yields, respectively. Interestingly, the reaction with **4b** gave **5b** with complete selectivity toward the internal alkene, which is in contrast to the mixtures observed under traditional enamine synthesis conditions with this substrate.^41^ Intrigued by this behavior, we tested other acyclic/cyclic unsymmetrical ketones (**4m**, **4n**, and **4o**) under these conditions. Using ethyl acetoacetate (**4m**) as the electrophile, more forceful conditions (160 °C, 1 h) were required to obtain reasonable conversion, and a modest yield (33%) of the internal enamine **5b** was isolated. In the case of unsymmetrical **5n** and **5o**, the isomer distribution was found to be dependent on the reaction temperature. In both cases, the reaction at 140 °C led to an inseparable mixture of both isomers (^1^H NMR analysis; see Supporting Information (SI), S42–S43). However, increasing the temperature to 160 °C and time to 1 h allowed isolation of the tetra-substituted enamines. This is again in contrast to usual preference for secondary amines to afford the least-substituted enamine upon condensation with a ketone.^42^

**Table 2:**
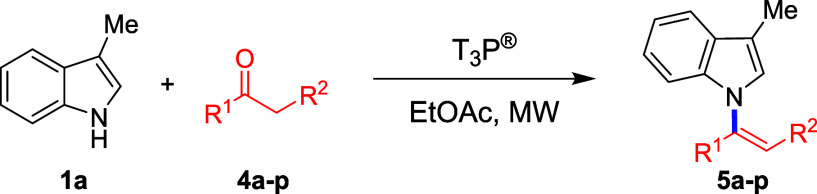

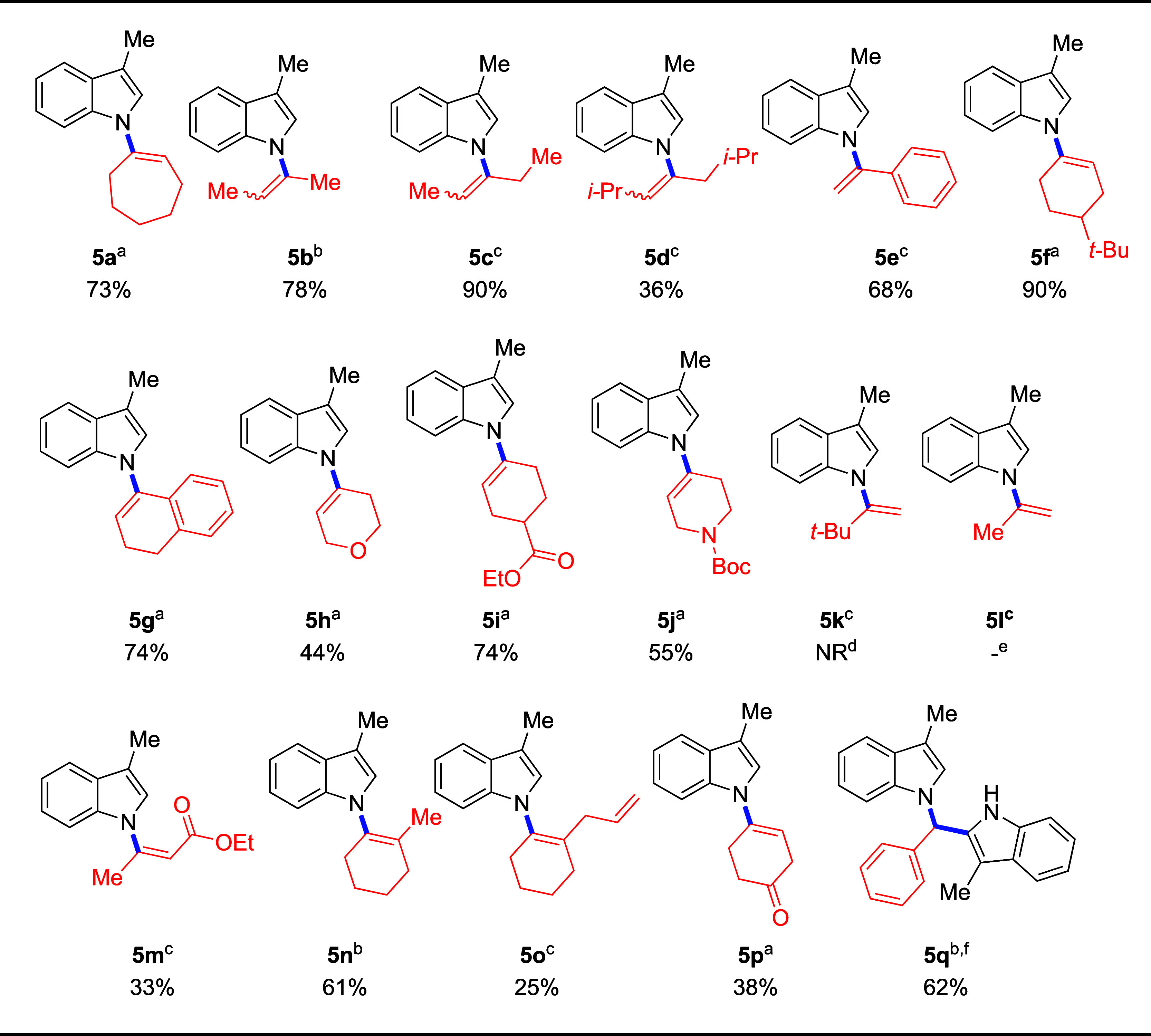
Substrate Scope Utilizing 3-Methylindole (**1a**) and Ketones^a^

a Isolated yields. Ketone (3 equiv), T_3_P 50 wt % in EtOAc (1.5 equiv), EtOAc (0.5 mL), 120 °C, 20 min.

b 140 °C, 20 min.

c Ketone (5 equiv), 160 °C, 1 h.

d No reaction.

e Complex mixture.

f Using benzaldehyde instead of a ketone.

As expected, lower reactivity was observed for acyclic ketones with greater steric bulk,^40^ as exemplified when comparing yields obtained from pinacolone (**4k**, no reaction observed), isobutyl ketone (**4d**, yield 36%) with 2-propone (**4b**, 78%, Table 2). Notably, heterocyclic scaffolds such as the oxan-4-one (**4h**) or the piperidone (**4j**) displayed high reactivity, and these reactions proceeded effectively even at room temperature. The reaction with 1,4-cyclohexanedione (**4p**) led to the formation of the monoalkenylated product **5p** in a moderate yield of 38%. Finally, when a nonenolizable aldehyde was used, as in the case of benzaldehyde (**4q**), the novel bisindole derivative **5q** was isolated in 62% yield. This likely proceeds via the initial formation of an iminium ion followed by an *aza*-Friedel–Crafts reaction from an additional indole nucleophile. Importantly, this represents a potentially powerful synthetic approach to access novel *N*-1,C2-linked isomers of C3,C3- and C2,C2-bis(indolyl)methanes, two widely explored scaffolds in medicinal chemistry and drug development.^43−45^

**Scheme 2:**
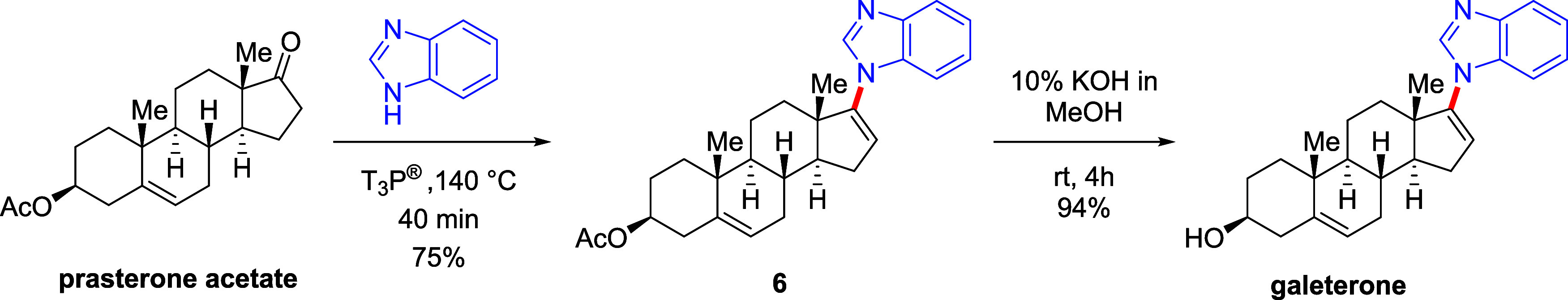
T_3_P-Promoted Synthesis of Galeterone

To demonstrate the utility of this protocol for the preparation of biologically active compounds, we selected the *N*-alkenyl-bearing drug galeterone^16^ as a synthetic target (Scheme 2). Galeterone is a steroidal antiandrogen and a potent inhibitor of the 17α-hydroxylase/17,20-lyase (CYP17), and currently used in the treatment of prostate cancer.^18^ The reported synthesis^16,17^ consists of four steps (Vilsmeier–Haack reaction/Cl-substitution/deformylation/hydrolysis), three of which require column chromatography purification, starting from the inexpensive starting material prasterone acetate, with an overall yield of 47%. However, with our protocol, we were able to synthesize galeterone in only two steps, with only one purification, in an overall yield of 68%. It is noteworthy that the key alkenylation reaction furnished the desired product **6** in a very good yield (75%), despite the high steric hindrance and competing electrophilic acetate center in prasterone acetate.

**Scheme 3:**
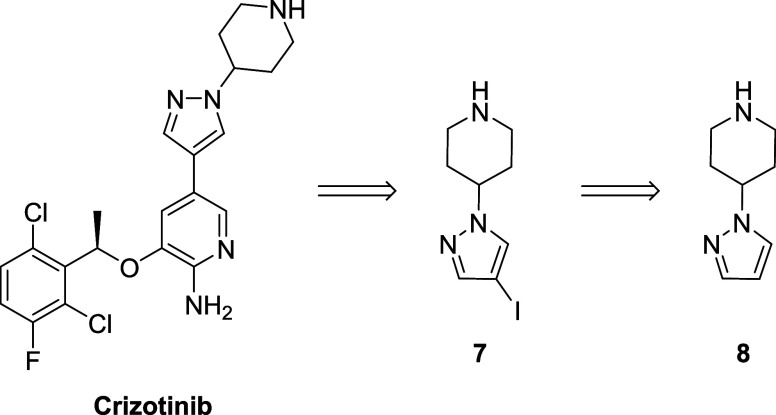
Structure of Antitumoral Drug Crizotinib and the Key Intermediate **7**

Furthermore, we believed that our T_3_P-promoted reaction could be useful for preparing compound **7** (Scheme 3), a key intermediate in the synthesis of crizotinib,^46^ an important antitumoral drug used for the treatment of non-small-cell lung carcinoma.^47^

**Scheme 4:**
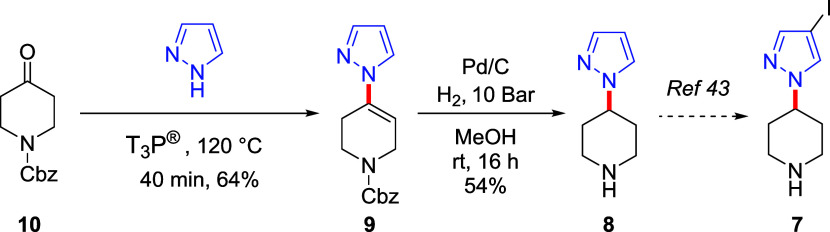
Synthesis of the Key Intermediate **8** Using Our T_3_P-Promoted Protocol

In the reported synthesis,^46^ the key intermediate **7** was easily obtained from 4-(1*H*-pyrazol-1-yl) piperidine (**8**) through iodination (Scheme 3). However, the formation of **8** involved a challenging pyridine reduction step, and this required long reaction times, high pressures, an expensive Rh-catalyst, and was highly sensitive to the presence of trace impurities.^38^ Therefore, we reasoned that our T_3_P protocol would provide an attractive alternative route to compound **8** by avoiding these drawbacks. To this end, we focused on the synthesis of the *N*-Cbz-protected piperidine derivative **9** starting from the corresponding piperidone **10** and pyrazole (Scheme 4). Pleasingly, the T_3_P-promoted reaction between the commercially available piperidone **10** and pyrazole afforded the alkenyl derivative **9** in a good yield (64%). Subsequent Cbz deprotection and enamine reduction using H_2_ and Pd/C gave iodination precursor **8** in an overall yield of 37%.

**Scheme 5:**
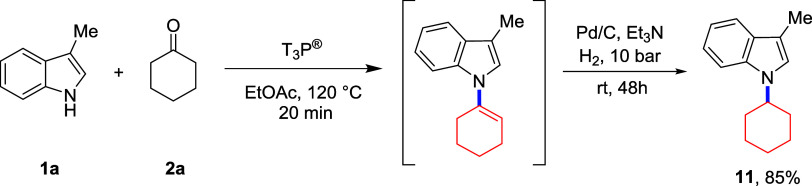
Telescoped Reductive Amination Sequence to Afford Indole **11**

Finally, we explored the possibility of reducing the formed *N*-alkenylated group in a telescoped fashion using 3-methylindole and cyclohexanone (Scheme 5). In this case, after completion of the alkenylation reaction, Pd/C and Et_3_N were directly added to the reaction mixture and stirred under a H_2_ atmosphere for 48 h. Gratifyingly, the expected *N*-alkylated product **11** was isolated in a yield of 85% over two steps. This reductive amination sequence represents a powerful new strategy for heterocycle *N*-alkylation that does not require strongly basic conditions and uses readily available ketone substrates.

**Scheme 6:**
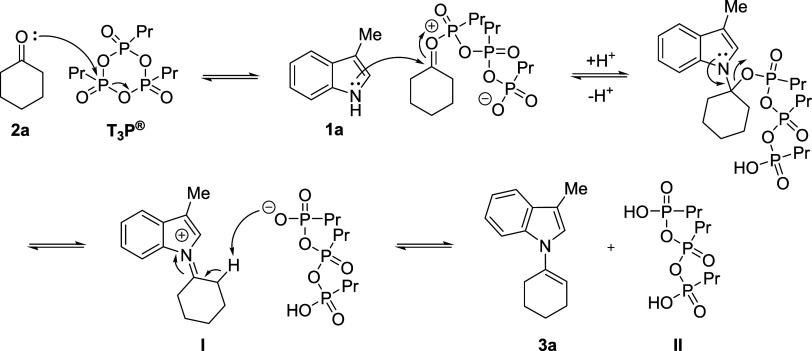
Suggested Mechanism for the T_3_P-Mediated Formation of **3a** from **1a** and **2b**

Mechanistically, the reaction is believed to proceed through an initial T_3_P-mediated activation of the ketone^38^ followed by nucleophilic addition of the *N*-heterocycle (Scheme 6). Subsequent elimination leads to the formation of iminium ion **I** and tautomerization affords the *N*-alkenylated product and the propylphosphonic acid byproduct **II**. Alternatively, compound **II** can act as an acid catalyst to activate the ketone for nucleophilic addition followed by T_3_P-mediated dehydration to give **I**. Given that the method relies on the innate nucleophilicity of the *N*-heterocycle, an excess of the ketone is required to afford high yields of the alkenylated products. Additionally, as the alkenyl motif is formed via tautomerization, the use of unsymmetrical ketones can lead to the formation of *E*/*Z* isomers. This is in contrast to other approaches using prefunctionalized alkenes where the stereochemical outcome is controlled by the configuration of the starting material^31^ or steric bias in an alkene-forming elimination step.^8^ Thus, while our approach offers the advantages of using abundant ketone electrophiles under metal- and base-free conditions, the trade-off is that it requires the use of excess electrophile, nucleophilic *N*-heterocycles, and does not allow for control of alkene configuration when using unsymmetrical substrates.

## Conclusions

In summary, we presented a convenient new synthesis of *N-*alkenylated heterocycles utilizing the eco-friendly coupling reagent T_3_P as a water scavenger and abundant ketone electrophiles under microwave irradiation. The protocol showcases several advantages, including metal and base-free conditions, ease of product purification, and good functional group tolerance. Demonstrating versatility, the methodology was exemplified in over 30 different *NH*-heterocycle and ketone coupling reactions with good to excellent yields (up to 94%). Furthermore, the application of this T_3_P-catalyzed protocol facilitated the development of a novel and improved two-step synthesis of galeterone, demonstrating its applicability to pharmaceutical-relevant scaffolds. Finally, a telescoped reductive amination process was devised to afford *N*-alkylated heterocycles from ketones in a one-pot fashion. We anticipate that these methodologies will offer appealing alternative strategies for accessing these valuable heterocyclic derivatives for future applications in organic and medicinal chemistry.

## Experimental Section

### General Chemistry Information

All reagents and solvents were of commercial quality and used without further purification. All reported yields are for isolated, homogeneous, and spectroscopically pure material. Silica gel chromatography was carried out on silica gel (60 Å pore size, particle size 40–63 nm) packed in glass columns. ^1^H NMR spectra were recorded at 400 MHz and ^13^C NMR spectra at 101 MHz. The chemical shifts (δ) for ^1^H NMR and ^13^C NMR were referenced to tetramethylsilane via residual solvent signals (^1^H: (CD_3_)_2_CO at 2.05 ppm, CDCl_3_ at 7.26 ppm, DMSO-*d*_6_ at 2.50 ppm and CD_3_CN at 2.01; ^13^C{^1^H}: (CD_3_)_2_CO at 25.8, 206.3 ppm, CDCl_3_ at 77.2 ppm, DMSO-*d*_6_ at 39.5 ppm, and CD_3_CN at 0.9 ppm). Structural assignments were made with additional information from the gCOSY, gHSQC, and gHMBC experiments. LC-UV/MS was performed on an instrument equipped with a CP-Sil8 CB capillary column (50 mm × 3.0 mm, particle size 2.6 μm, pore size 100 Å) running at an ionization potential of 70 eV with a CH_3_CN/H_2_O gradient (0.05% HCOOH). Accurate mass values were determined via electrospray ionization with a 7-T hybrid ion trap and a time-of-flight (TOF) detector running in positive or negative mode. All reactions requiring heat were performed under microwave conditions in a Biotage Initiator, and their temperature was determined using the built-in online infrared (IR)-sensor. All reactions were performed in sealed microwave-transparent vials designed for 0.2 and 2.0 mL reaction volumes.

### General Procedure

In a 0.2–2.0 mL Biotage Microwave reaction vial charged with a solution of the heterocycle (0.70 mmol, 1 equiv) in EtOAc (0.500 mL), T3P (50 wt % solution in EtOAc, 1.05 mmol, 1.50 equiv) and the appropriate ketone were added. The vial was capped and heated under MW conditions by following one of these procedures:

### Procedure A

Heterocycle (1 equiv), ketone/aldehyde (3 equiv), 20 min, 120 °C, MW

### Procedure B

Heterocycle (1 equiv), ketone/aldehyde (3 equiv), 20 min, 140 °C, MW

### Procedure C

Heterocycle (1 equiv), ketone/aldehyde (5 equiv), 60 min, 160 °C, MW

After the reaction had reached completion, triethylamine (0.5 mL) was added. The mixtures were filtered through a silica plug to obtain the desired product. An additional gradient separation with column chromatography was occasionally required.

#### 1-(Cyclohex-1-en-1-yl)-3-methyl-1*H*-indole (**3a**)

Synthesized according to the general procedure A. Isolated using flash column chromatography (silica gel, 2% EtOAc in isohexane) as a clear oil (139 mg, 94%), from 3-methylindole **1a** (92 mg, 0.70 mmol) and cyclohexanone **2a** (206 mg, 2.10 mmol). ^1^H NMR (400 MHz, (CD_3_)_2_CO) δ 7.51 (ddd, *J* = 7.8, 1.3, 0.8 Hz, 1H), 7.46 (ddd, *J* = 8.3, 1,0, 0.9 Hz, 1H), 7.13 (ddd, *J* = 8.3, 7.0, 1.3 Hz 1H), 7.09 (q, *J* = 1.1 Hz, 1H), 7.04 (ddd, *J* = 7.8, 7.0, 1.0 Hz, 1H), 5.89–5.83 (m, 1H), 2.48–2.41 (m, 2H), 2.32–2.24 (m, 2H), 2.28 (d, *J* = 1.1 Hz, 3H), 1.90–1.82 (m, 2H), 1.77–1.69 (m, 2H). ^13^C{^1^H} NMR (101 MHz, (CD_3_)_2_CO) δ: 136.8, 136.8, 130.2, 125.2, 122.4, 120.6, 119.8, 119.5, 111.8, 111.5, 29.4, 25.1, 23.7, 22.8, 9.6. High-resolution mass spectrometry (HRMS) (ESI+) *m*/*z* [M = C_15_H_17_N]: [M + H]^+^ calcd 212.1439, found 212.1439.

#### Methyl 2-(1-(Cyclohex-1-en-1-yl)-1*H*-indol-3-yl)acetate (**3b**)

Synthesized according to general procedure A. Isolated using flash column chromatography (silica gel, 5% EtOAc in isohexane) as a white solid (141 mg, 85%), from methyl 2-(1*H*-indol-3-yl)acetate **1b** (120 mg, 0.62 mmol) and cyclohexanone **2a** (181 mg, 1.85 mmol). ^1^H NMR (400 MHz, CDCl_3_) δ 7.62 (ddd, *J* = 7.8, 1.2, 1.0 Hz, 1H), 7.49 (ddd, *J* = 8.3, 1.0, 1.0 Hz, 1H), 7.24–7.12 (m, 3H), 5.93–5.84 (m, 1H), 3.79 (d, *J* = 0.9 Hz, 2H), 3.72 (s, 3H), 2.48–2.43 (m, 2H), 2.32–2. Twenty-eight (m, 2H), 1.94–1.88 (m, 2H), 1.79–1.73 (m, 2H). ^13^C{^1^H} NMR (101 MHz, CDCl_3_) δ: 172.4, 135.8, 135.7, 128.0, 125.5, 121.9, 121.3, 119.6, 118.9, 111.2, 107.7, 51.9, 31.1, 28.8, 24.5, 22.9, 22.0. HRMS (ESI+) *m*/*z* [M = C_17_H_20_NO_2_]: [M + H]^+^ calcd 270.1494, found 270.1498.

#### 1-(Cyclohex-1-en-1-yl)-1*H*-pyrazole (**3c**)^25^ CAS 25834–38–2

Synthesized according to the general procedure A. Isolated using flash column chromatography (silica gel, gradient elution 0–2% EtOAc in isohexane) as a yellow oil (63 mg, 85%), from pyrazole **1c** (34 mg, 0.50 mmol) and cyclohexanone **2a** (147 mg, 1.50 mmol). ^1^H NMR(400 MHz, (CD_3_)_2_CO) δ 7.63 (dd, *J* = 2.4, 0.7 Hz, 1H), 7.29–7.28 (m, 1H), 6.10 (dd, *J* = 2.5, 1.7 Hz, 1H), 5.94–5.92 (m, 1H), 2.40–2.35 (m, 2H), 2.04–1.96 (m, 2H), 1.63–1.57 (m, 2H), 1.47–1.41 (m, 2H). ^13^C{^1^H} NMR (101 MHz, (CD_3_)_2_CO) δ 138.8, 136.5, 125.5, 112.1, 105.4, 25.3, 23.5, 22.0, 21.6.

#### 1-(Cyclohex-1-en-1-yl)-1*H*-1,2,4-triazole (**3d**)

Synthesized according to the general procedure A. Isolated using flash column chromatography (silica gel, gradient elution 0–30% EtOAc in isohexane) as a yellow oil (57 mg, 75%), from 1,2,4-triazole **1d** (36 mg, 0.51 mmol) and cyclohexanone **2a** (150 mg, 1.53 mmol). ^1^H NMR (400 MHz, CDCl_3_) δ 8.21 (s, 1H), 7.95 (s, 1H), 6.24–6.22 (m, 1H), 2.55–2.51 (m, 2H), 2.25–2.21 (m, 2H), 1.88–1.82 (m, 2H), 1,71–1.66 (m, 2H). ^13^C{^1^H} NMR (101 MHz, CDCl_3_) δ 151.8, 140.0, 134.1, 117.7, 26.1, 24.1, 22.2, 21.8. HRMS (ESI+) *m*/*z* [M = C_8_H_12_N_3_]: [M + H]^+^ calcd 150.1031, found 150.1028.

#### 1-(Cyclohex-1-en-1-yl)-1*H*-imidazole (**3e**)^25^ CAS 74199–41–0

Synthesized according to the general procedure A. Isolated using flash column chromatography (silica gel, 75% EtOAc in isohexane) as an off-white solid (77 mg, 70%), from imidazole **1e** (51 mg, 0.74 mmol) and cyclohexanone **2a** (218 mg, 2.22 mmol). ^1^H NMR (400 MHz, CDCl_3_) δ 7.63 (s, 1H), 7.04 (d, *J* = 8.8 Hz, 2H), 5.82–5.79 (m, 1H), 2.42–2.37 (m, 2H), 2.19–2.13 (m, 2H), 1.84–1.77 (m, 2H), 1.68–1.61 (m, 2H). ^13^C{^1^H} NMR (101 MHz, CDCl_3_) δ 134.4, 133.7, 129.2, 116.5, 116.4, 27.3, 24.1, 22.3, 21.6.

#### 1-(Cyclohex-1-en-1-yl)-1*H*-indazole (**3f′**) and 2-(Cyclohex-1-en-1-yl)-2*H*-indazole (**3f″**)

Synthesized according to the general procedure A. Isolated using flash column chromatography (silica gel, 2% EtOAc in isohexane) yielding 1-(cyclohex-1-en-1-yl)-1*H*-indazole as a white solid (49 mg, 46% yield) and 2-(cyclohex-1-en-1-yl)-2*H*-indazole as a white solid (45 mg, 42% yield, isomer structure elucidation by NOESY) from indazole **1f** (63 mg, 0.53 mmol) and cyclohexanone **2a** (157.0 mg, 1.60 mmol).

#### 1-(Cyclohex-1-en-1-yl)-1*H*-indazole (**3f**′)

^1^H NMR (400 MHz, CDCl_3_) δ. 8.05 (d, *J* = 1.0 Hz, 1H), 7.72 (ddd, *J* = 8.1, 1.0, 1.0 Hz, 1H), 7.63 (ddd, *J* = 8.6, 1.5, 0.9 Hz, 1H), 7.36 (ddd, *J* = 8.5, 6.9, 1.1 Hz, 1H), 7.15 (ddd, *J* = 7.9, 6.9, 0.9 Hz, 1H), 6.04–6.01 (m, 1H), 2.64–2.68 (m, 2H), 2.34–2.29 (m, 2H), 1.92–1.86 (m, 2H), 1.79–1.73 (m, 2H). ^13^C{^1^H} NMR (101 MHz, CDCl_3_) δ: 138.6, 137.0, 133.6, 126.4, 124.5, 121.0, 120.9, 119.1, 111.0, 27.7, 24.4, 22.7, 22.0. HRMS: HRMS (ESI+) *m*/*z* [M = C_13_H_15_N_2_]: [M + H]^+^ calcd 199.1235, found 199.1248.

#### 2-(Cyclohex-1-en-1-yl)-2*H*-indazole (**3f″**)

^1^H NMR (400 MHz, CDCl_3_) δ 8.08 (d, *J* = 1.0 Hz, 1H), 7.70 (ddd, *J* = 8.8, 1.2, 1.0 Hz, 1H), 7.63 (ddd, *J* = 8.4, 1.1, 1.1 Hz, 1H), 7.32–7.22 (ddd, *J* = 8.8, 6.6, 1.1 Hz, 1H), 7.05 (ddd, *J* = 8.4, 6.6, 0.9 Hz, 1H), 6.48–6.45 (m, 1H), 2.74–2.69 (m, 2H), 2.32–2.27 (m, 2H), 1.92–1.86 (m, 2H), 1.75–1.69 (m, 2H). ^13^C{^1^H} NMR (101 MHz, CDCl_3_) δ: 148.7, 137.3, 126.3, 121.8, 121.7, 120.2, 119.5, 118.6, 117.5, 26.5, 24.3, 22.4, 21.8. HRMS (ESI+) *m*/*z* [M = C_13_H_15_N_2_]: [M + H]^+^ calcd 199.1235, found 199.1241.

#### 1-(Cyclohex-1-en-1-yl)-1*H*-benzo[*d*][1,2,3]triazole (**3g′**) and 1-(Cyclohex-1-en-1-yl)-2*H*-benzo[*d*][1,2,3]triazole (**3g″**)

Synthesized according to the general procedure A. Isolated using flash column chromatography (silica gel, 10% EtOAc in isohexane) yielding 2-(cyclohex-1-en-1-yl)-1*H*-benzo[*d*][1,2,3]triazole as a pale yellow oil (67 mg, 48%) and 2-(cyclohex-1-en-1-yl)-2*H*-benzo[*d*][1,2,3]triazole as a white solid (44 mg, 32% yield) from 1*H*-benzotriazole **1g** (83 mg, 0.70 mmol) and cyclohexanone **2a** (206 mg, 2.10 mmol).

#### 2-(Cyclohex-1-en-1-yl)-1*H*-benzo[*d*][1,2,3]triazole, (**3g′**)^48^ CAS 73006–66–3

^1^H NMR (400 MHz, (CD_3_)_2_CO) δ 8.03 (ddd, *J* = 8.3, 1.1, 1.1 Hz, 1H), 7.83 (ddd, *J* = 8.4, 1.0, 1.0 Hz, 1H), 7.56 (ddd, *J* = 8.3, 6.9, 1.1 Hz, 1H), 7.42 (ddd, *J* = 8.4, 6.9, 1.0 Hz, 1H), 6.28–6.26 (m, 1H), 2.81–2.74 (m, 2H), 2.39–2.35 (m, 2H), 1.97–1.91 (m, 2H), 1.81–1.75 (m, 2H). ^13^C {^1^H} NMR (101 MHz, (CD_3_)_2_CO) δ 146.0, 135.1, 131.9, 127.6, 124.0, 120.8, 119.6, 111.4, 27.3, 24.1, 22.3, 21.5.

#### 2-(Cyclohex-1-en-1-yl)-2*H*-benzo[*d*][1,2,3]triazole, (**3g″**)^48^ CAS 2414619–05–7

^1^H NMR (400 MHz, (CD_3_)_2_CO) δ 7.91–7.82 (m, 2H), 7.46–7.38 (m, 2H), 7.00–6.98 (m, 1H), 2.93–2.88 (m, 2H), 2.38–2.33 (m, 2H), 1.94–1.88 (m, 2H), 1.77–1.71 (m, 2H). ^13^C {^1^H} NMR (101 MHz, (CD_3_)_2_CO) δ 144.1, 138.1, 126.6, 120.1, 118.0, 25.2, 24.0, 22.1, 21.5.

#### 1-(Cyclohex-1-en-1-yl)-1*H*-benzo[*d*]imidazole (**3h**)^49^ CAS 1451090–71–3

Synthesized according to the general procedure A. Isolated using flash column chromatography (silica gel, 25% EtOAc in isohexane) as an off-white solid (74 mg, 75%), from benzimidazole **1h** (59 mg, 0.50 mmol) and cyclohexanone **2a** (147 mg, 1.50 mmol). ^1^H NMR (400 MHz, (CD_3_)_2_CO) δ 8.11 (s, 1H), 7.68–7.66 (m, 1H), 7.58–7.56 (m, 1H), 7.27–7.22 (m, 2H), 6.05–6.03 (m, 1H), 2.58–2.54 (m, 2H), 2.33–2.30 (m, 2H), 1.92–1.88 (m, 2H), 1.77–1.75 (m, 2H). ^13^C{^1^H} NMR (101 MHz, (CD_3_)_2_CO) δ 144.2, 141.8, 133.7, 133.4, 122.7, 122.0, 121.7, 119.9, 111.1, 28.1, 24.1, 22.5, 21.5.

#### 1-(Cyclohexen-1-yl)-5,6-dimethyl-benzimidazole (**3i**)

Synthesized according to the general procedure C. Isolated using flash column chromatography (silica gel, 30% EtOAc in isohexane) as an off-white solid (95 mg, 60%), from 5,6-dimethyl-1*H*-benzimidazole **1i** (102 mg, 0.70 mmol) and cyclohexanone **2a** (344 mg, 3.50 mmol). ^1^H NMR (400 MHz, (CD_3_)_2_CO) δ 7.96 (s, 1H), 7.44 (s, 1H), 7.36 (s, 1H), 6.01–5.98 (m, 1H), 2.56–2.51 (m, 2H), 2.35 (s, 3H), 2.33 (s, 3H) 2.31–2.27 (m, 2H), 1.91–1.85 (m, 2H), 1.77–1.71 (m, 2H). ^13^C{^1^H} NMR (101 MHz, (CD_3_)_2_CO) δ 143.8, 141.8, 134.8, 132.9, 132.5, 131.3, 122.0, 120.9, 112.3, 28.9, 25.0, 23.4, 22.4, 20.5, 20.2. HRMS (ESI+) *m*/*z* [M = C_15_H_18_N_2_]: [M + H]^+^ calcd 227.1548, found 227.1554.

#### 1-(Cyclohex-1-en-1-yl)-5-(trifluoromethyl)-1*H*-benzo[*d*]imidazole (**3j′**) and 1-(Cyclohex-1-en-1-yl)-6-(trifluoromethyl)-1*H*-benzo[*d*]imidazole (**3j″**)

Synthesized according to the general procedure B, the reaction time increased to 1 h. Isolated using flash column chromatography (silica gel, gradient elution 30% EtOAc in isohexane) as a pale red solid 1-(cyclohex-1-en-1-yl)-5-(trifluoromethyl)-1*H*-benzo[d]imidazole (61 mg, 36.5% yield, isomer structure elucidation by NOESY) (**3j″**) and 1-(cyclohex-1-en-1-yl)-6-(trifluoromethyl)-1*H*-benzo[d]imidazole as a pale red solid (80 mg, 43% yield) from 6-(trifluoromethyl)-1*H*-benzimidazole **1j** (130 mg, 0.70 mmol) and cyclohexanone **2a** (206 mg, 2.10 mmol).

#### 1-(Cyclohex-1-en-1-yl)-5-(trifluoromethyl)-1*H*-benzo[*d*]imidazole (**3j′**)

^1^H NMR (400 MHz, (CD_3_)_2_CO) δ ^1^H NMR (400 MHz, (CD_3_)_2_CO) δ 8.32 (s, 1H), 8.02 (m, 1H), 7.78–7.75 (m, 1H), 7.57 (dd, *J* = 8.5, 1.8 Hz, 1H), 6.09 (m, 1H), 2.59–2.53 (m, 2H), 2.34–2.28 (m, 2H), 1.92–1.86 (m, 2H), 1.78–1.72 (m, 2H). ^13^C NMR (101 MHz, (CD_3_)_2_CO) δ: 144.3, 143.6, 135.7, 133.3, 126.5, 123.8, 123.5, 119.5, 117.4, 112.1, 28.1, 24.2, 22.4, 21.4. HRMS (ESI+) *m*/*z* [M = C_14_H_13_F_3_N_2_]: [M + H] ^+^ calcd 267.1103, found 267.1107

#### 1-(Cyclohex-1-en-1-yl)-6-(trifluoromethyl)-1*H*-benzo[*d*]imidazole (**3j″**)

^1^H NMR (400 MHz, (CD_3_)_2_CO) δ 8.37 (s, 1H), 7.95–7.94 (m 1H), 7.90–7.87 (m, 1H), 7.59–7.56 (m, 1H), 6.15–6.12 (m, 1H), 2.62–2.57 (m, 2H), 2.35–2.31 (m, 2H), 1.94–1.89 (m, 2H), 1.78–1.74 (m, 2H). ^13^C NMR (101 MHz, (CD_3_)_2_CO) δ: 146.5, 144.8, 133.3, 133.0, 124.5, 123.7, 120.7, 118.6, 112.1, 108.9, 28.2, 24.2, 22.4, 21.3. HRMS (ESI+) *m*/*z* [M = C_14_H_13_F_3_N_2_]: [M + H] ^+^ calcd 267.1103, found 267.1103

#### 9-(Cyclohex-1-en-1-yl)-9*H*-carbazole (**3k**)

Synthesized according to the general procedure C. Isolated using flash column chromatography (silica gel, 10% EtOAc in isohexane) as a white solid (156 mg, 87%) from carbazole **1j** (119 mg, 0.71 mmol) and cyclohexanone **2a** (349 mg, 3.56 mmol). ^1^H NMR (400 MHz, DMSO) δ 8.15 (ddd, *J* = 7.7, 1.0, 1.0 Hz, 2H), 7.45–7.40 (m, 4H), 7.22 (ddd, *J* = 7.7, 5.1, 2.5 Hz, 2H), 6.05–6.03 (m, 1H), 2.36–2.31 (m, 2H), 2.30–2.25 (m, 2H) 1.92–1.84 (m, 2H), 1.82–1.74 (m, 2H). ^13^C{^1^H} NMR (101 MHz, DMSO) δ: 139.8, 133.7, 127.9, 125.8, 122.3, 120.3, 119.2, 109.8, 26.9, 24.5, 22.5, 21.5. HRMS (ESI+) *m*/*z* [M = C_18_H_18_N]: [M + H]^+^ calcd 248.1439, found 248.1432

#### 9-(Cyclohexen-1-yl)-1,2,3,4-tetrahydrocarbazole (**3l**)

Synthesized according to the general procedure C, heated for 2 h instead of 1 h. Isolated using flash column chromatography (silica gel, 5% EtOAc in isohexane) as a white solid (142 mg, 81%) from 2,3,4,9-tetrahydro-1*H*-carbazole **1k** (120 mg, 0.70 mmol) and cyclohexanone **2a** (344 mg, 3.50 mmol). ^1^H NMR (400 MHz, (CD_3_)CO) δ 7.38 (ddd, *J* = 7.5, 1.0, 1.0 Hz, 1H), 7.19 (ddd, *J* = 8.1, 1.0 1.0 Hz, 1H), 7.08–6.92 (m, 2H), 5.82–5.77 (m, 1H), 2.70–2.62 (m, 4H), 2.32–2.27 (m, 2H) 2.26–2.21 (m, 2H), 1.92–1.80 (m, 6H), 1.75 (m, 2H). ^13^C{^1^H} NMR (101 MHz, (CD_3_)CO) δ 137.5, 135.9, 135.7, 128.5, 127.8, 121.4, 119.5, 118.2, 110.4, 110.1, 29.6, 25.5, 24.1, 24.0, 23.7, 23.2, 22.6, 21.7. HRMS (ESI+) *m*/*z* [M = C_18_H_21_N]: [M + H]^+^ calcd 252.1747, found 252.1735.

#### 10-(Cyclohexen-1-yl)phenoxazine (**3m**)

Synthesized according to the general procedure C. Isolated using flash column chromatography (silica gel, 100% isohexane) as white solid (148 mg, 80%) from 10*H*-phenoxazine **1l** (128 mg, 0.70 mmol) and cyclohexanone **2a** (344 mg, 3.50 mmol). ^1^H NMR (400 MHz, CDCl_3_) δ 6.75–6.66 (m, 2H), 6.63–6.59 (m, 4H), 6.42–6.40 (m, 2H), 5.92–5.90 (m, 1H), 2.31–2.24 (m, 2H), 2.10–2.05 (m, 2H), 1.90–1.80 (m, 2H), 1.77–1.69 (m, 2H). ^13^C{^1^H} NMR (101 MHz, CDCl_3_) δ: 144.3, 134.7, 132.7, 132.4, 123.5, 121.0, 115.5, 112.8, 42.1, 27.2, 25.3, 25.2, 24.3, 23.0, 22.0. HRMS (ESI+) *m*/*z* [M = C_18_H_17_NO]: [M]^+^ calcd 263.1310, found 263.1313.

#### Methyl Nα-(((9*H*-Fluoren-9-yl)methoxy)carbonyl)-1-(cyclohex-1-en-1-yl)tryptophanate, (**3n**)

Synthesized according to the general procedure C. Isolated using flash column chromatography (silica gel, 20% EtOAc in pentane) as a pale yellow oil (182 mg, 85%) from Fmoc-Trp-OMe **1m** (154 mg, 0.35 mmol) and cyclohexanone **2a** (172 mg, 1.75 mmol). ^1^H NMR (400 MHz, CD_3_CN) δ 7.82 (d, *J* = 7.6 Hz, 2H), 7.63–7.57 (m, 3H), 7.49 (ddd, *J* = 8.3, 0.9, 0.9 Hz, 1H), 7.41 (ddd, *J* = 7.4, 0.9, 0.9 Hz, 2H), 7.30 (ddd, *J* = 8.7, 7.4, 1.2 Hz, 2H), 7.18 (ddd, *J* = 8.3, 7.0, 1.3 Hz, 1H), 7.12–7.08 (m, 2H), 6.05 (d, *J* = 8.3 Hz, 1H), 5.85–5.82 (m, 1H), 4.57 (ddd, *J* = 8.3, 8.1, 5.2 Hz, 1H), 4.33 (dd, *J* = 10.5, 7.1 Hz, 1H), 4.25 (dd, *J* = 10.5, 7.0 Hz, 1H), 4.16 (dd, *J* = 10.5, 7.1 Hz, 1H) 3.69 (s, 3H), 3.30 (dd, *J* = 14.7, 5.3 Hz, 1H), 3.17 (dd, *J* = 14.7, 8.0 Hz, 1H), 2.41–2.33 (m, 2H), 2.26–2.18 (m, 2H), 1.84–1.76 (m, 2H), 1.75–1.65 (m, 2H). ^13^C{^1^H} NMR (101 MHz, CD_3_CN) δ: 173.0, 156.4, 144.63, 144.56, 141.7, 136.4, 136.1, 128.8, 128.3, 127.7, 126.2, 125.8, 125.7, 122.5, 121.4, 120.5, 120.1, 119.2, 111.8, 110.7, 66.9, 55.3, 52.4, 47.6, 29.0, 27.9, 24.7, 23.2, 22.2. α_D_^20^ = +23.51° (c 1.17, CH_2_Cl_2_); HRMS (ESI+) *m*/*z* [M = C_33_H_33_N_2_O_4_]: [M + H]^+^ calcd 521.2440, found 521.2430.

#### 3-Benzyl-1-(cyclohex-1-en-1-yl)-1*H*-indole (**3o**)

Synthesized according to the general procedure B. Isolated using flash column chromatography (silica gel, 5% EtOAc in isohexane) as a yellow oil (68 mg, 64% yield) from 3-benzyl-1*H*-indole **1n** (77 mg, 0.37 mmol) and cyclohexanone **2a** (109 mg, 1.11 mmol). ^1^H NMR (400 MHz, (CD_3_)_2_CO) δ 7.35–7.34 (m, 1H), 7.33–7.31 (m, 1H), 7.20–7.18 (m, 2H), 7.15–7.10 (m, 2H), 7.05–6.95 (m, 3H), 6.85 (ddd, *J* = 7.9, 7.0, 1.0 Hz, 1H), 5.75–5.73 (m, 1H), 3.95 (s, 2H), 2.35–2.30 (m, 2H), 2.19–2.12 (m, 2H), 1.76–1.69 (m, 2H), 1.63–1.56 (m, 2H). ^13^C{^1^H} NMR (101 MHz, (CD_3_)_2_CO) δ 141.5, 136.2, 135.9, 128.6, 128.4, 128.2, 125.7, 124.8, 121.7, 120.2, 119.1, 115.2, 111.0, 31.1, 28.5, 24.2, 22.8, 21.8. HRMS (ESI+) *m*/*z* [M = C_21_H_21_N]: [M + H]^+^ calcd 288.1752, found 288.1747.

#### 1-(Cyclohex-1-en-1-yl)-3-phenyl-1*H*-indole (**3p**)

Synthesized according to the general procedure B. Isolated using flash column chromatography (silica gel, 5% EtOAc in isohexane) yielding 1-(cyclohex-1-en-1-yl)-3-phenyl-1*H*-indole as a pale yellow oil (52 mg, 82%) from 3-phenyl-1*H*-indole **1o** (45 mg, 0.23 mmol) and cyclohexanone **2a** (68 mg, 0.70 mmol). ^1^H NMR (400 MHz, CDCl_3_) δ 7.94 (ddd, *J* = 7.9, 1.3, 0.8 Hz, 1H), 7.69–7.66 (m, 2H), 7.52 (ddd, *J* = 8.3, 1.0, 1.0 Hz, 1H), 7.47–7.43 (m, 2H), 7.34 (s, 1H) 7.31–7.26 (m, 1H) 7.25–7.17 (m, 2H), 6.01–5.98 (m, 1H), 2.52–2.48 (m, 2H), 2.35–2.30 (m, 2H), 1.94–1.88 (m, 2H), 1.81–1.75 (m, 2H). ^13^C{^1^H} NMR (101 MHz, CDCl_3_) δ: 136.5, 135.7, 135.5, 128.8, 127.5, 126.5, 125.9, 124.5, 122.2, 122.1, 120.2, 119.9, 117.5, 111.4, 29.0, 24.6, 23.0, 22.0. HRMS (ESI+) *m*/*z* [M = C_20_H_19_N]: [M + H]^+^ calcd 274.1596, found 274.1599.

#### 1,3-Di(cyclohex-1-en-1-yl)-1*H*-indole (**3q**)

Synthesized according to the general procedure A. Isolated using flash column chromatography (silica gel, gradient elution 1% EtOAc in isohexane) as a brown oil (135 mg, 69% yield) from indole **1q** (82 mg, 0.70 mmol) and cyclohexanone **2a** (206 mg, 2.10 mmol). ^1^H NMR (400 MHz, (CD_3_)_2_CO) δ 7.84–7.81 (m, 1H), 7.47–7.42 (m, 1H), 7.24 (s, 1H), 7.15–7.02 (m, 2H), 6.23–6.20 (m, 1H), 5.88–5.81 (m, 1H), 2.44–2.39 (m, 4H), 2.27–2.19 (m, 4H), 1.86–1.76 (m, 4H), 1.72–1.64 (m, 4H).^13^C NMR (101 MHz, (CD_3_)_2_CO) δ 13C NMR (101 MHz, acetone) δ 136.6, 135.8, 131.3, 126.4, 123.7, 121.7, 121.6, 121.3, 120.7, 119.7, 118.6, 111.1, 28.2, 26.7, 25.5, 24.3, 23.1, 22.8, 22.4, 21.8. HRMS (ESI+) *m*/*z* [M = C_20_H_23_N]: [M + H] ^+^ calcd 278,1903, found 278.1906

#### 1-(Cyclohept-1-en-1-yl)-3-methyl-1*H*-indole (**5a**)^50^ CAS 2122299–51–6

Synthesized according to the general procedure A. Isolated using flash column chromatography (silica gel, 100% isohexane) as a white solid (115 mg, 73%) from 3-methyl-1*H*-indole **1a** (92 mg, 0.70 mmol) and cycloheptanone **4a** (236 mg, 2.10 mmol). ^1^H NMR (400 MHz, (CD_3_)_2_CO) δ 7.51 (ddd, *J* = 7.8, 1.3, 0.8 Hz, 1H), 7.36 (ddd, *J* = 8.3, 1.1, 0.9 Hz, 1H), 7.14 (ddd, *J* = 8.3, 7.0, 1.3 Hz, 1H), 7.04 (ddd, *J* = 7.8, 7.0, 1.2 Hz, 1H), 7.00 (q, *J* = 1.2 Hz, 1H), 5.94 (t, *J* = 6.7 Hz, 1H), 2.70–2.60 (m, 2H), 2.35–2.29 (m, 2H), 2.28 (d, *J* = 1.1 Hz, 3H), 1.91–1.82 (m, 2H), 1.81–1.70 (m, 2H), 1.70–1.61 (m, 2H). ^13^C{^1^H} NMR (101 MHz, (CD_3_)_2_CO) δ 143.2, 136.8, 130.3, 126.1, 125.7, 122.4, 119.7, 119.6, 111.7, 111.4, 34.4, 32.7, 27.7, 27.5, 27.1, 9.6.

#### 1-(But-2-en-2-yl)-3-methyl-1*H*-indole (**5b**)

Synthesized according to general procedure B, using 5 equiv of 2-butanone. Isolated using flash column chromatography (silica gel, 0.5% EtOAc in isohexane) yielding 1-(but-2-en-2-yl)-3-methyl-1*H*-indole (inseparable 1:1 mixture of *E* and *Z* isomers) as a yellow oil (71 mg, 78%) from 3-methyl-1*H*-indole **1a** (66 mg, 0.50 mmol) and 2-butanone **4b** (180 mg, 2.50 mmol). ^1^H NMR (400 MHz,(CD_3_)_2_CO) δ 7.54 (ddd, *J* = 7.8, 1.0, 1.0 Hz, 1H), 7.51 (ddd, *J* = 7.8, 1.3, 0.8 Hz, 1H), 7.40 (ddd, *J* = 8.2, 0.9, 0.9 p Hz, 1H), 7.18–7.10 (m, 3H), 7.08–7.02 (m, 3H), 6.99 (q, *J* = 1.1 Hz, 1H), 5.70 (qq, *J* = 7.0, 1.3 Hz, 1H), 5.65 (qq, *J* = 7.1, 1.2 Hz, 1H), 2.32 (d, *J* = 1.1 Hz, 3H), 2.29 (d, *J* = 1.1 Hz, 3H), 2.14–2.13 (m, 3H), 2.08–2.06 (m, 3H), 1.84 (dq, *J* = 7.0, 1.2 Hz, 3H), 1.38 (dq, *J* = 6.8, 1.6 Hz, 3H). ^13^C{^1^H} NMR (101 MHz,(CD_3_)_2_CO) δ 136.0, 135.7, 133.7, 129.2, 128.6, 124.7, 124.7, 121.5, 121.5, 120.5, 118.8, 118.7, 118.6, 118.6, 118.0, 111.0, 110.7, 110.5, 110.4, 110.0, 21.7, 15.8, 12.5, 12.2, 8.8, 8.7. HRMS (ESI+) *m*/*z* [M = C_13_H_15_N]: [M + H]^+^ calcd 186.1283, found 186.1278.

#### 3-Methyl-1-(pent-2-en-3-yl)-1*H*-indole (**5c**)

Synthesized according to the general procedure C, heated for 20 min instead of 60. Isolated using flash column chromatography (silica gel, 0.5% EtOAc in isohexane) yielding 3-methyl-1-(pent-2-en-3-yl)-1*H*-indole (inseparable 3:1 mixture of *Z* and *E* isomers determined by selective gradient NOESY experiment) as a yellow oil (90 mg, 90%) from 3-methylindole **1a** (66 mg, 0.50 mmol) and 3-pentanone **4c** (215 mg, 2.50 mmol). ^1^H NMR (400 MHz, (CD_3_)_2_CO) δ 7.54 (ddd, *J* = 7.8, 1.0, 1.0 Hz, 1H), 7.51 (ddd, *J* = 7.8, 1.3, 0.8 Hz, 0.3H), 7.37–7.35 (m, 0.3H), 7.17–7.10 (m, 3H), 7.07–7.02 (m, 1.6H), 6.97 (q, *J* = 1.1 Hz, 1H), 5.72 (qt, *J* = 6.8, 1.2 Hz, 1H), 5.63–5.58 (m, 0.3H), 2.63–2.57 (m, 0.8H), 2.48–2.42 (m, 2H), 2.33 (d, *J* = 1.1 Hz, 3H), 2.30 (d, *J* = 1.1 Hz, 1H), 1.86 (dt, *J* = 7.0, 0.8 Hz, 1H), 1.38 (dt, *J* = 6.8, 1.4 Hz, 3H), 0.86 (t, *J* = 7.6 Hz, 3H) 0.83 (t, *J* = 7.6 Hz, 1H). ^13^C{^1^H} NMR (101 MHz,(CD_3_)_2_CO) δ 139.4, 139.4, 136.9, 136.4, 129.0, 128.5, 125.1, 124.9, 121.5, 121.5, 119.4, 118.9, 118.7, 118.7, 118.6, 118.6, 110.9, 110.5, 110.4, 110.4, 29.4, 23.1, 12.3, 11.9, 11.5, 11.2, 8.8, 8.7. HRMS (ESI+) *m*/*z* [M = C_14_H_17_N]: [M + H]^+^ calcd 200.1439, found 200.1440.

#### 1-(2,6-Dimethylhept-3-en-4-yl)-3-methyl-1*H*-indole (**5d**)

Synthesized according to general procedure C, using 5 equiv of 2,6-dimethylheptan-4-one **4d**. Isolated using flash column chromatography (silica gel, 100% isohexane) yielded 1-(2,6-dimethylhept-3-en-4-yl)-3-methyl-1*H*-indole (inseparable 1:1 mixture of *E* and *Z* isomers) as a yellow oil (65 mg, 36%) from 3-methyl-1*H*-indole **1a** (91.8 mg, 0.700 mmol) and 2,6-dimethylheptan-4-one **4d** (498 mg, 0.615 mL, 3.50 mmol). ^1^H NMR (400 MHz, CDCl_3_) δ 7.58–7.57 (m, 1H), 7.56–7.55 (m, 1H) 7.40–7.39 (m, 1H), 7.23–7.15 (m, 3H), 7.13–7.07 (m, 2H), 6.94 (q, *J* = 1.1 Hz, 1H), 6.78 (q, *J* = 1.1 Hz, 1H), 5.40–5.35 (m 2H), 2.67–2.56 (m, 1H), 2.34 (d, *J* = 1.1 Hz, 3H), 2.33 (d, *J* = 1.1 Hz, 3H), 2.15–2.14 (m, 2H), 2.05–2.04 (m, 2H), 1.72–1. 65 (m, 1H), 1.29–1.23 (m,2H), 1.09–1.07 (m, 6H), 0.99–0.88 (m, 12H), 0.74 (d, *J* = 6.6 Hz, 3H), 0.56 (d, *J* = 6.5 Hz, 3H). ^13^C{^1^H} NMR (101 MHz, CDCl_3_) δ: 135.0, 132.0, 131.5, 129.2, 128.7, 125.4, 124.9, 121.8, 121.6, 119.1, 118.9, 118.8, 111.1, 111.0, 110.5, 47.1, 47.1, 30.7, 29.9, 26.1, 25.8, 23.4, 23.4, 22.6, 22.4, 21.7, 21.6, 17.2, 9.7, 9.7. HRMS (ESI+) *m*/*z* [M = C_18_H_26_N]: [M + H]^+^ calcd 256.2065, found 256.2062

#### 3-Methyl-1-(1-phenylvinyl)-1*H*-indole (**5e**)^51^ CAS 1176684–23–3

Synthesized according to the general procedure C. Isolated using flash column chromatography (silica gel, 0.5% EtOAc in isohexane) as a clear oil (78 mg, 68%) from 3-methylindole **1a** (66 mg, 0.50 mmol) and acetophenone **4e** (294 mg, 2.45 mmol). ^1^H NMR (400 MHz, (CD_3_)_2_CO) δ 7.58 (ddd, *J* = 7.7, 1.6, 0.8 Hz, 1H), 7.44–7.34 (m, 3H), 7.33–7.27 (m, 2H), 7.12–7.01 (m, 3H), 7.03–6.98 (m, 1H), 5.57 (d, *J* = 0.5 Hz, 1H), 5.33 (d, *J* = 0.5 Hz, 1H), 2.33 (d, *J* = 1.2 Hz, 3H). ^13^C{^1^H} NMR (101 MHz,(CD_3_)_2_CO) δ 145.1, 137.3, 136.7, 129.9, 129.1, 128.6, 126.9, 126.3, 121.9, 119.5, 118.9, 111.9, 111.6, 107.0, 8.8.

#### 1-(4-*tert*-Butylcyclohexen-1-yl)-3-methyl-indole (**5f**)

Synthesized according to the general procedure A. Isolated using flash column chromatography (silica gel, 5% EtOAc in isohexane) as a white solid (169 mg, 90% yield) from 3-methylindole **1a** (91.8 mg, 0.70 mmol) and 4-*tert*-butylcyclohexanone **4f** (324 mg, 2.10 mmol). ^1^H NMR (400 MHz, (CD_3_)_2_CO) δ 7.53 (ddd, *J* = 7.8, 1.1, 1.1 Hz, 1H), 7.49 (ddd, *J* = 8.3, 1.0, 1.0 Hz, 1H), 7.15 (ddd, *J* = 8.3, 7.0, 1.3 Hz, 1H), 7.09–7.05 (m, 2H), 5.88–5.85 (m, 1H), 2.53–2.49 (m, 2H), 2.36–2.32 (m, 1H), 2.31 (d, *J* = 1.1 Hz, 3H), 2.12–2.00 (m, 2H), 1.54–1.39 (m, 2H), 0.97 (s, 9H). ^13^C{^1^H} NMR (101 MHz, (CD_3_)_2_CO) δ: 136.8, 136.6, 130.2, 125.1, 122.4, 120.3, 119.8, 119.5, 111.8, 111.5, 44.6, 32.7, 30.6, 27.6, 26.7, 25.1, 9.6. HRMS (ESI+) *m*/*z* [M = C_19_H_26_N]: [M + H]^+^ calcd 268.2065, found 268.2076.

#### 1-(3,4-Dihydronaphthalen-2-yl)-3-methyl-indole (**5g**)

Synthesized according to general procedure A. Isolated using flash column chromatography (silica gel, 5% EtOAc in isohexane) as a clear oil (134 mg, 74% yield) from 3-methylindole **1a** (91.8 mg, 0.70 mmol) and tetralin-2-one **4g** (307 mg, 2.10 mmol). ^1^H NMR (400 MHz, (CD_3_)_2_CO) δ 7.68 (ddd, *J* = 8.3, 0.9, 0.9 Hz, 1H), 7.59 (ddd, *J* = 7.8, 1.1, 1.1 Hz, 1H), 7.30–7.21 (m, 2H), 7.21–7.11 (m, 5H), 6.69–6.68 (m, 1H), 3.06 (dd, *J* = 9.2, 7.0 Hz, 2H), 2.90–2.84 (m, 2H), 2.34 (d, *J* = 1.2 Hz, 3H). ^13^C{^1^H} NMR (101 MHz, (CD_3_)_2_CO) δ 138.0, 135.7, 134.1, 133.2, 130.2, 127.2, 126.7, 126.5, 126.1, 124.0, 122.2, 119.8, 119.0, 116.1, 112.3, 111.9, 28.2, 27.2, 8.8. HRMS (ESI+) *m*/*z* [M = C_19_H_18_N]: [M + H]^+^ calcd 260.1439, found 260.1434.

#### 1-(3,6-Dihydro-2*H*-pyran-4-yl)-3-methyl-1*H*-indole (**5h**)

Synthesized according to general procedure A, but instead of heating, the reaction mixture was stirred at room temperature for 3 h. Isolated using flash column chromatography (silica gel, 15% CH_2_Cl_2_ in pentane) as a yellow solid (66 mg, 44% yield) from 3-methylindole **1a** (91.8 mg, 0.70 mmol) and tetrahydropyran-4-one **4h** (210 mg, 2.10 mmol). ^1^H NMR (400 MHz, (CD_3_)_2_CO) δ 7.56 (ddd, *J* = 8,3, 0.9, 0.9 Hz, 1H), 7.54 (ddd, *J* = 7.7, 1.1, 1.1 Hz, 1H), 7.22–7.15 (m, 2H), 7.09 (ddd, *J* = 8.0, 7.0, 1.0 Hz, 1H), 5.93–5.92 (m, 1H), 4.35–4.33z (m, 2H), 3.96–3.94 (m 2H), 2.62–2.58 (m, 2H), 2.30 (d, *J* = 1.2 Hz, 3H). ^13^C{^1^H} NMR (101 MHz, (CD_3_)_2_CO) δ 135.7, 133.3, 129.7, 123.6, 121.9, 119.4, 118.8, 116.5, 111.5, 111.1, 64.3, 64.0, 28.6, 8.7. HRMS (ESI+) *m*/*z* [M = C_14_H_15_NO]: [M + H]^+^ calcd 214.1232, found 214.1238.

#### Ethyl 4-(3-Methyl-1*H*-indol-1-yl)cyclohex-3-ene-1-carboxylate (**5i**)

Synthesized according to the general procedure A and heated for 60 min. Isolated using flash column chromatography (silica gel, 10% EtOAc in pentane) as a yellow oil (147 mg, 74% yield) from 3-methylindole **1a** (91.8 mg, 0.70 mmol) and ethyl 4-oxocyclohexanecarboxylate **4i** (357 mg, 2.10 mmol). ^1^H NMR (400 MHz, (CD_3_)_2_CO) δ 7.52–7.46 (m, 2H), 7.16–7.09 (m, 2H), 7.05 (ddd, *J* = 8.0, 7.0, 1.1 Hz, 1H), 5.88–5.87 (m, 1H), 4.15 (q, *J* = 7.3 Hz, 2H), 2.79–2.71 (m, 1H), 2.61–2.50 (m, 4H), 2.28 (d, *J* = 1.1 Hz, 3H), 2.23–2.16 (m, 1H), 2.01–1.91 (m, 1H), 1.26 (t, *J* = 7.1 Hz, 3H).^13^C{^1^H} NMR (101 MHz, (CD_3_)_2_CO) δ 174.3, 136.0, 135.5, 129.4, 124.2, 121.6, 119.1, 118.7, 117.8, 110.9, 59.9, 38.5, 27.5, 26.6, 25.3, 13.7, 8.7. HRMS (ESI+) *m*/*z* [M = C_18_H_21_NO_2_]: [M + H]^+^ calcd 284.1651, found 284.1639.

#### *tert*-Butyl 4-(3-Methylindol-1-yl)-3,6-dihydro-2*H*-pyridine-1-carboxylate (**5j**)

Synthesized according to general procedure A, but instead of heating, the reaction mixture was stirred at room temperature for 4 h. Isolated using flash column chromatography (silica gel, 40% CH_2_Cl_2_ in pentane) as a yellow oil (120 mg, 55% yield) from 3-methylindole **2a** (91.8 mg, 0.70 mmol) and *tert*-butyl 4-oxopiperidine-1-carboxylate **4j** (418 mg, 2.10 mmol). ^1^H NMR (400 MHz, (CD_3_)_2_CO) δ 7.56–7.55 (m, 1H), 7.54–7.53 (m, 1H), 7.20–7.16 (m, 2H), 7.10 (ddd, *J* = 8.0, 7.0, 0.8 Hz, 1H), 5.93–5.92 (m, 1H), 4.18–4.17 (m, 2H), 3.76–3.73 (m, 2H), 2.66–2.61 (m, 2H), 2.31 (d, *J* = 1.1 Hz, 3H), 1.51 (s, 9H).^13^C{^1^H} NMR (101 MHz, (CD_3_)_2_CO) δ 152.0, 135.7, 129.6, 123.9, 121.9, 119.3, 118.8, 111.4, 111.0, 80.8, 79.0, 40.9, 27.7, 23.5, 20.4, 16.9, 16.9, 8.7, 7.3. HRMS (ESI+) *m*/*z* [M = C_19_H_24_N_2_O_2_]: [M + H]^+^ calcd 313.1916, found 313.1925.

#### Ethyl (*E*)-3-(3-Methyl-1*H*-indol-1-yl)but-2-enoate (**5m**)

Synthesized according to the general procedure C and heated for 20 min. Isolated using flash column chromatography (silica gel, 2% EtOAc in pentane) as a yellow oil (56 mg, 33% yield) from 3-methylindole **1a** (91.8 mg, 0.70 mmol) and ethyl acetoacetate **4m** (455 mg, 3.50 mmol). ^1^H NMR (400 MHz, (CD_3_)_2_CO) δ δ 7.71 (ddd, *J* = 8.4, 1.0, 0.9 Hz, 1H), 7.57 (ddd, *J* = 7.8, 1.3, 0.7 Hz, 1H), 7.39 (q, *J* = 1.2 Hz, 1H), 7.28 (ddd, *J* = 8.4, 7.1, 1.4 Hz, 1H), 7.18 (ddd, *J* = 8.0, 7.1, 1.0 Hz, 1H), 6.05 (q, *J* = 0.9 Hz, 1H), 4.19 (q, *J* = 7.2 Hz, 2H), 2.80 (d, *J* = 0.9 Hz, 3H), 2.30 (d, *J* = 1.2 Hz, 3H), 1.28 (td, *J* = 7.1 Hz, 3H).^13^C{^1^H} NMR (101 MHz, (CD_3_)_2_CO) δ 166.5, 151.6, 135.4, 131.3, 123.9, 123.1, 121.0, 119.3, 114.6, 112.6, 106.6, 59.4, 17.6, 13.8, 8.6. HRMS (ESI+) *m*/*z* [M = C_15_H_17_NO_2_]: [M + H]^+^ calcd 244.1338, found 244.1346.

#### 3-Methyl-1-(2-methylcyclohex-1-en-1-yl)-1*H*-indole (**5n**)

Synthesized according to general procedure B. Isolated using flash column chromatography (silica gel, 1% EtOAc in pentane) as a yellow oil (95 mg, 61% yield) from 3-methylindole **1a** (91.8 mg, 0.70 mmol) and 2-methylcyclohexanone **2a** (236 mg, 2.10 mmol). ^1^H NMR (400 MHz, (CD_3_)_2_CO) δ 7.54–7. 51 (m, 1H), 7.15–7.02 (m, 3H), 6.96–6.95 (m, 1H), 2.32–2.25 (m, 5H), 2.24–2.20 (m, 2H), 1.86–1.75 (m, 4H), 1.40–1.39 (m, 3H).^13^C{^1^H} NMR (101 MHz, (CD_3_)_2_CO) δ 136.1, 131.2, 129.5, 128.4, 125.1, 121.3, 118.6, 118.5, 110.4, 110.1, 30.6, 29.7, 23.2, 22.5, 17.8, 8.8. HRMS (ESI+) *m*/*z* [M = C_16_H_19_N]: [M + H]^+^ calcd 226.1596, found 226.1587.

#### 1-(2-Allylcyclohex-1-en-1-yl)-3-methyl-1*H*-indole (**5o**)

Synthesized according to general procedure C. Isolated using flash column chromatography (silica gel, 100% pentane) as a yellow oil (44 mg, 25% yield) from 3-methylindole **1a** (91.8 mg, 0.70 mmol) and 2-ethylcyclohexanone **4o** (484 mg, 3.50 mmol). ^1^H NMR (400 MHz, (CD_3_)_2_CO) δ 7.53 (ddd, *J* = 7.8, 1.0, 0.9 Hz, 1H), 7.19–7.08 (m, 2H), 7.05 (ddd, *J* = 8.0, 6.9, 1.4 Hz, 1H), 6.95 (q, *J* = 1.1 Hz, 1H), 5.66 (ddt, *J* = 18.1, 9.1, 6.8 Hz, 1H), 4.95–4. 94 (m, 1H), 4.93–4.89 (m, 1H), 2.53–2.47 (m, 2H), 2.33–2.31 (m, 5H), 2.26–2.23 (m, 2H), 1.87–1.81 (m, 2H), 1.80–1.73 (m, 2H). ^13^C {^1^H} NMR (101 MHz, (CD_3_)_2_CO) δ 136.3, 136.0, 134.0, 134.0, 130.6, 128.6, 125.5, 121.4, 118.6, 115.6, 110.4, 110.0, 36.5, 29.7, 28.0, 23.1, 22.4, 8.8. HRMS (ESI+) *m*/*z* [M = C_18_H_21_N]: [M + H]^+^ calcd 252.1752, found 252.1742.

#### 4-(3-Methyl-1*H*-indol-1-yl)cyclohex-3-en-1-one (**5p**)

Synthesized according to the general procedure, but instead of heating in the microwave at 120 °C, the temperature was lowered to 80 °C for 20 min. Isolated using flash column chromatography (silica gel, gradient elution 15% EtOAc in isohexane) as a beige solid (60 mg, 38% yield) from 3-methylindole **1a** (91.8 mg, 0.70 mmol) and 1,4-cyclohexanedione **2p** (235 mg, 2.10 mmol). ^1^H NMR (400 MHz, (CD_3_)_2_CO) δ 7.57–7.51 (m, 2H), 7.20–7.14 (m, 2H), 7.08 (ddd, J = 8.0, 7.0, 1.0 Hz, 1H), 5.95 (m, 1H), 3.13–3.11 (m, 2H), 3.02–2.96 (m, 2H), 2.73 (m, 2H), 2.30 (d, *J* = 1.1 Hz, 3H). ^13^C NMR (101 MHz, (CD_3_)_2_CO) δ: 207.7, 136.8, 136.8, 130.5, 125.3, 122.8, 120.2, 119.7, 116.5, 112.4, 112.0, 39.0, 38.9, 9.6. HRMS (ESI+) *m*/*z* [M = C_15_H_15_NO]: [M + H] ^+^ calcd 226,1233, found 226.1230

#### 3-Methyl-2-((3-methyl-1*H*-indol-1-yl)(phenyl)methyl)-1*H*-indole (**5q**)

Synthesized according to the general procedure B. Isolated using flash column chromatography (silica gel, gradient elution 0–10% EtOAc in isohexane) as a white solid (48 mg, 62% yield) from 3-methylindole **1a** (91.8 mg, 0.70 mmol) and benzaldehyde **4p** (223 mg, 2.10 mmol). ^1^H NMR (400 MHz, (CD_3_)_2_CO) δ 9.78 (s, 1H), 7.57–7.50 (m, 2H), 7.39–7.26 (m, 5H), 7.24 (s, 1H), 7.18–7.13 (m, 2H), 7.12–7.00 (m, 4H), 6.86 (q, *J* = 1.1 Hz, 1H), 2.25 (d, *J* = 1.1 Hz, 3H), 2.18 (s, 3H). ^13^C {^1^H} NMR (101 MHz, (CD_3_)_2_CO) δ 140.5, 137.7, 137.1, 133.0, 130.1, 129.8, 129.6, 128.6, 128.3, 125.2, 122.7, 122.3, 119.8, 119.8, 119.6, 119.4, 112.1, 111.1, 110.8, 110.2 56.9, 9.8, 8.6. HRMS (ESI+) *m*/*z* [M = C_25_H_23_N_2_]: [M + H]^+^ calcd 351.1861, found 351.1852.

#### Larger-Scale Synthesis of 1-(Cyclohex-1-en-1-yl)-3-methyl-1*H*-indole (**3a**)

Synthesized according to the general procedure A. Isolated using flash column chromatography (silica gel, gradient elution 0–2% EtOAc in isohexane) as a clear oil (250 mg, 84%), from 3-methylindole **1a** (185 mg, 1.41 mmol) and cyclohexanone **2a** (412 mg, 4.23 mmol). ^1^H NMR (400 MHz, (CD_3_)_2_CO) δ 7.52 (ddd, *J* = 7.8, 1.2, 0.8 Hz, 1H), 7.48 (ddd, *J* = 8.3, 1.0, 0.9 Hz, 1H), 7.14 (ddd, *J* = 8.3, 7.0, 1.3 Hz, 1H), 7.11 (q, *J* = 1.1 Hz, 1H), 7.06 (ddd, *J* = 8.0, 7.0, 1.0 Hz, 1H), 5.89–5.86 (m, 1H), 2.49–2.44 (m, 2H), 2.33–2.26 (m, 5H), 1.91–1.85 (m, 2H), 1.78–1.72 (m, 2H).^13^C{^1^H} NMR (101 MHz, (CD_3_)_2_CO) δ 135.9, 135.9, 129.3, 124.3, 121.5, 119.8, 118.9, 118.7, 110.9, 110.7, 28.5, 24.2, 22.8, 21.9, 8.7.

#### 3β-Acetoxy-17-(1*H*-benzimidazol-1-yl)-androsta-5,16-diene (**6**)^52^ CAS 851895–79–9

Synthesized according to general procedure B and heated for 40 min. Isolated using flash column chromatography (silica gel, isohexane-AcOEt-Et_3_N 7.5:3.0:0.5 v/v) as a clear oil (98 mg, 75% yield) from benzimidazole **1h** (179 mg, 1.51 mmol) and prasterone acetate (100 mg, 0.30 mmol). ^1^H NMR (400 MHz, CDCl_3_) δ 7.89 (s, 1H), 7.77–7.72 (m, 1H), 7.45–7.39 (m, 1H), 7.26–7.20 (m, 2H), 5.91 (dd, *J* = 3.2, 1.7 Hz, 1H), 5.37–5.35 (m, 1H), 4.59–4.51 (m, 1H), 2.41–2.21 (m, 3H), 2.18–2.01 (m, 2H), 1.97 (s, 3H), 1.87–1.40 (m, 10H), 1.20–1.05 (m, 2H), 1.01 (s, 3H), 0.95 (s, 3H). ^13^C{^1^H} NMR (101 MHz, CDCl_3_) δ: 170.6, 147.2, 143.3, 141.7, 140.1, 134.6, 124.2, 123.4, 122.5, 122.0, 120.2, 111.1, 73.8, 55.8, 50.4, 47.2, 38.1, 36.9, 36.8, 34.8, 31.1, 30.4, 30.3, 27.7, 21.4, 20.6, 19.3, 16.0.

#### Galeterone^52^ CAS 851983–85–2

3β-Acetoxy-17-(1*H*-benzimidazol-1-yl)-androsta-5,16-diene **6** (20 mg, 0.05 mmol) was dissolved in methanol (0.5 mL), and the resulting solution was treated with 10% methanolic KOH (12.4 μL). The mixture was stirred at room temperature for 1.5 h and then concentrated under reduced pressure. This solution was poured into ice water (8 mL), and the resulting white precipitate was filtered, washed with water, and dried affording galeterone as a white solid (17 mg, 94%). ^1^H NMR (400 MHz, CDCl_3_) δ 7.89 (s, 1H), 7.76–7.67 (m, 1H), 7.45–7.36 (m, 1H), 7.26–7.23 (m, 2H), 5.93 (dd, *J* = 3.3, 1.7 Hz, 1H), 5.34–5.32 (m, 1H), 3.50–3.41 (m, 1H), 2.41–2.01 (m, 5H), 1.83–1.37 (m, 11H), 1.10–1.02 (m, 2H), 0.99 (s, 3H), 0.95 (s, 3H). ^13^C{1H} NMR (101 MHz, CDCl_3_) δ 147.0, 142.6, 141.5, 141.3, 134.4, 124.6, 123.6, 122.7, 120.9, 119.8, 111.2, 71.3, 55.9, 50.5, 48.6, 47.2, 42.0, 37.1, 36.7, 34.8, 31.3, 31.1, 30.3, 20.6, 19.3, 15.9.

#### Benzyl 4-(1*H*-Pyrazol-1-yl)-3,6-dihydropyridine-1(2*H*)-carboxylate (**9**)

Synthesized according to the general procedure A. Isolated using flash column chromatography (silica gel, 50% Et_2_O in isohexane) as a yellow oil (140 mg, 64% yield) from pyrazole **1c** (157 mg, 2.31 mmol) and benzyl 4-oxopiperidine-1-carboxylate (180 mg, 0.77 mmol). ^1^H NMR (400 MHz, CDCl_3_) δ 7.64–7.62 (m 2H), 7.43–7.32 (m, 5H), 6.37 (t, *J* = 2.1 Hz, 1H), 6.07–6.05 (m, 1H), 5.20 (s, 2H), 4.21–4.19 (m, 2H), 3.82–3.79 (m, 2H), 2.79–2.75 (m, 2H). ^13^C {^1^H} NMR (101 MHz, CDCl_3_) δ 155.2, 140.3, 139.9, 136.6, 135.2, 128.6, 128.1, 128.0, 126.9, 126.0, 109.7, 106.7, 67.3, 42.3, 40.1, 25.8. HRMS (ESI+) *m*/*z* [M = C_16_H_17_N_3_O_2_]: [M + H]^+^ calcd 284.1399, found 284.1389.

#### 4-(1*H*-Pyrazol-1-yl)piperidine (**8**)^46^ CAS 762240–09–5

To a solution of benzyl 4-pyrazol-1-yl-3,6-dihydro-2*H*-pyridine-1-carboxylate (70 mg, 0.25 mmol) in methanol (3 mL) were added two drops of acetic acid, and the resulting suspension was hydrogenated over Pd/C (10 wt %) catalyst (15 mg, 0.14 mmol) at 10 bar for 48 h at room temperature. The reaction mixture was filtered over Celite, and the solvent was removed under reduced pressure, affording the 4-(1*H*-pyrazol-1-yl) piperidine acetate salt a yellow solid (28 mg, 54% yield) without any further purification. ^1^H NMR (400 MHz, CDCl_3_) δ 7.96 (bs, 2H), 7.45 (d, *J* = 1.9 Hz, 1H), 7.39 (d, *J* = 2.4 Hz, 1H), 6.21 (t, *J* = 2.1 Hz, 1H), 4.32–4.24 (m, 1H), 3.37–3.32 (m, 2H), 2.90–2.82 (m, 2H), 2.22–2.18 (m, 2H), 2.13–2.02 (m, 2H), 1.94 (s, 3H). ^13^C {^1^H} NMR (101 MHz, CDCl_3_) δ: 177.5, 139.1, 126.6, 105.6, 57.2, 43.4, 30.9, 23.1.

#### 1-Cyclohexyl-3-methyl-1*H*-indole (**11**)^53^ CAS 1037739–68–6

Compound **3a** was synthesized according to general procedure A, from 3-methylindole **1a** (103 mg, 0.79 mmol) and cyclohexanone **2a** (231 mg, 2.37 mmol). Once cooled, Et_3_N (0.3 mL, 1.95 mmol) and Pd/C (10% w/w) (83.6 mg, 0.08 mmol) were added. The reaction mixture was then hydrogenated under 10 bar of H_2_ at room temperature for 48 h. Isolated using flash column chromatography (silica gel, 0.5% EtOAc in isohexane as a colorless oil; 142 mg, 85% over two steps). ^1^H NMR (400 MHz, CDCl_3_) δ 7.58 (ddd, *J* = 7.8, 1.3, 0.8 Hz, 1H), 7.39–7.32 (m, 1H), 7.20 (ddd, *J* = 8.2, 6.9, 1.2 Hz, 1H), 7.10 (ddd, *J* = 7.9, 7.0, 1.0 Hz, 1H), 7.01 (q, *J* = 1.1 Hz, 1H), 4.22–4.14 (m, 1H), 2.35 (d, *J* = 1.0 Hz, 3H), 2.17–2.08 (m, 2H), 2.00–1.89 (m, 2H), 1.83–1.76 (m, 1H), 1.75–1.65 (m, 2H), 1.55–1.43 (m, 2H), 1.34–1.23 (m, 1H). ^13^C {^1^H} NMR (101 MHz, CDCl_3_) δ 135.8, 128.5, 121.7, 121.0, 119.0, 118.4, 110.0, 109.2, 54.8, 33.6, 26.0, 25.7, 9.7.

## Data Availability

The data underlying this study are available in the published article and its Supporting Information.

## Supporting Information Available

NMR spectra (^1^H NMR, ^13^C{^1^H} NMR) of compounds **3a**–**3q**, **5a**–**5j**, **5m–q**, **6, 8, 9, 11**, and galaterone (PDF)
