# Supplementary material for: Synthesis of N-Alkenylated Heterocycles via T3P-Promoted Condensation with Ketones
Source: J Org Chem. 2024 Jul 31;89(16):11203–14. doi: 10.1021/acs.joc.4c00803 (PMC11334184; doi:10.1021/acs.joc.4c00803)
Supplement: Supplementary file 1 — jo4c00803_si_001.pdf [file jo4c00803_si_001.pdf]

SUPPORTING INFORMATION

Synthesis of *N*-Alkenylated Heterocycles via T_3P®-Promoted Condensation with Ketones

*Lorenzo Jacopo Ilic Balestri, Julia Beveridge, Johan Gising, Luke R. Odell,**

Department of Medicinal Chemistry, Uppsala University, Box-574, SE-751 23 Uppsala, Sweden

*Corresponding author: luke.odell@ilk.uu.se Telephone: +46 018-471 4297

Contents

Copies of NMR Spectra 4

1-(cyclohex-1-en-1-yl)-3-methyl-1H-indole, 3a 4

Methyl 2-(1-(cyclohex-1-en-1-yl)-1H-indol-3-yl)acetate, 3b 5

1-(cyclohex-1-en-1-yl)-1H-pyrazole, 3c CAS 25834-38-2[1] 6

1-(cyclohex-1-en-1-yl)-1H-pyrazole, 3d 7

1-(cyclohex-1-en-1-yl)-1H-imidazole, 3e CAS 74198-41-0[1] 8

1-(cyclohex-1-en-1-yl)-1H-indazole, 3f-1 9

2-(cyclohex-1-en-1-yl)-2H-indazole, 3f-2 11

1-(cyclohex-1-en-1-yl)-1H-benzo[d][1,2,3]triazole , 3g-1 CAS 73006-66-3[2] 13

1-(cyclohex-1-en-1-yl)-2H-benzo[d][1,2,3]triazole, 3g-2 CAS 2414619-05-7[2] 14

1-(cyclohex-1-en-1-yl)-1H-benzo[d]imidazole, 3h CAS 1451090-71-3[3] 15

1-(Cyclohexen-1-yl)-5,6-dimethyl-benzimidazole, 3i 16

1-(cyclohex-1-en-1-yl)-5-(trifluoromethyl)-1H-benzo[d]imidazole,3j-1 17

1-(cyclohex-1-en-1-yl)-6-(trifluoromethyl)-1H-benzo[d]imidazole 3j-2 19

9-(Cyclohex-1-en-1-yl)-9H-carbazole, 3k 20

9-(cyclohexen-1-yl)-1,2,3,4-tetrahydrocarbazole, 3l 21

10-(cyclohexen-1-yl)phenoxazine, 3m 22

Methyl Nα-(((9H-fluoren-9-yl)methoxy)carbonyl)-1-(cyclohex-1-en-1-yl)tryptophanate, 3n 23

3-benzyl-1-(cyclohex-1-en-1-yl)-1H-indole, 3o 24

1-(cyclohex-1-en-1-yl)-3-phenyl-1H-indole, 3p 25

1,3-di(cyclohex-1-en-1-yl)-1H-indole, 3q 26

1-(cyclohept-1-en-1-yl)-3-methyl-1H-indole, 5a CAS 2122299-51-6 [4] 27

1-(but-2-en-2-yl)-3-methyl-1H-indole, 5b 28

3-methyl-1-(pent-2-en-3-yl)-1H-indole, 5c 29

1-[(Z)-1-isobutyl-3-methyl-but-1-enyl]-3-methyl-indole and 1-[(E)-1-isobutyl-3-methyl-but-1-enyl]-3-methyl-indole, 5d 31

3-methyl-1-(1-phenylvinyl)-1H-indole, 5e CAS1176684-23-3[5] 32

1-(4-tert-butylcyclohexen-1-yl)-3-methyl-indole, 5f 33

1-(3,4-dihydronaphthalen-2-yl)-3-methyl-indole, 5g 34

1-(3,6-dihydro-2H-pyran-4-yl)-3-methyl-1H-indole), 5h 35

Ethyl 4-(3-methyl-1H-indol-1-yl)cyclohex-3-ene-1-carboxylate, 5i 36

Tert-butyl 4-(3-methylindol-1-yl)-3,6-dihydro-2H-pyridine-1-carboxylate, 5j 37

ethyl (E)-3-(3-methyl-1H-indol-1-yl)but-2-enoate, 5m....................38
3-methyl-1-(2-methylcyclohex-1-en-1-yl)-1H-indole, 5n....................40
1-(2-allylcyclohex-1-en-1-yl)-3-methyl-1H-indole, 5o....................41
4-(3-methyl-1H-indol-1-yl)cyclohex-3-en-1-one, 5p....................44
Large scale synthesis of 1-(Cyclohex-1-en-1-yl)-3-methyl-1H-indole, 3a....................46
3β-acetoxy-17-(1H-benzimidazol-1-yl)-androsta-5,16-diene, 6 CAS 851895-79-9[6]....................47
Synthesis of Galeterone, CAS 851983-85-2[6]....................48
Benzyl 4-(1H-pyrazol-1-yl)-3,6-dihydropyridine-1(2H)-carboxylate, 9....................49
4-(1H-pyrazol-1-yl)piperidine, 8 CAS 762240-09-5[7]....................50
1-Cyclohexyl-3-methyl-1H-indole, 11 CAS 1037739-68-6[8]....................51
References....................52

Copies of NMR Spectra

1-(cyclohex-1-en-1-yl)-3-methyl-1H-indole, 3a

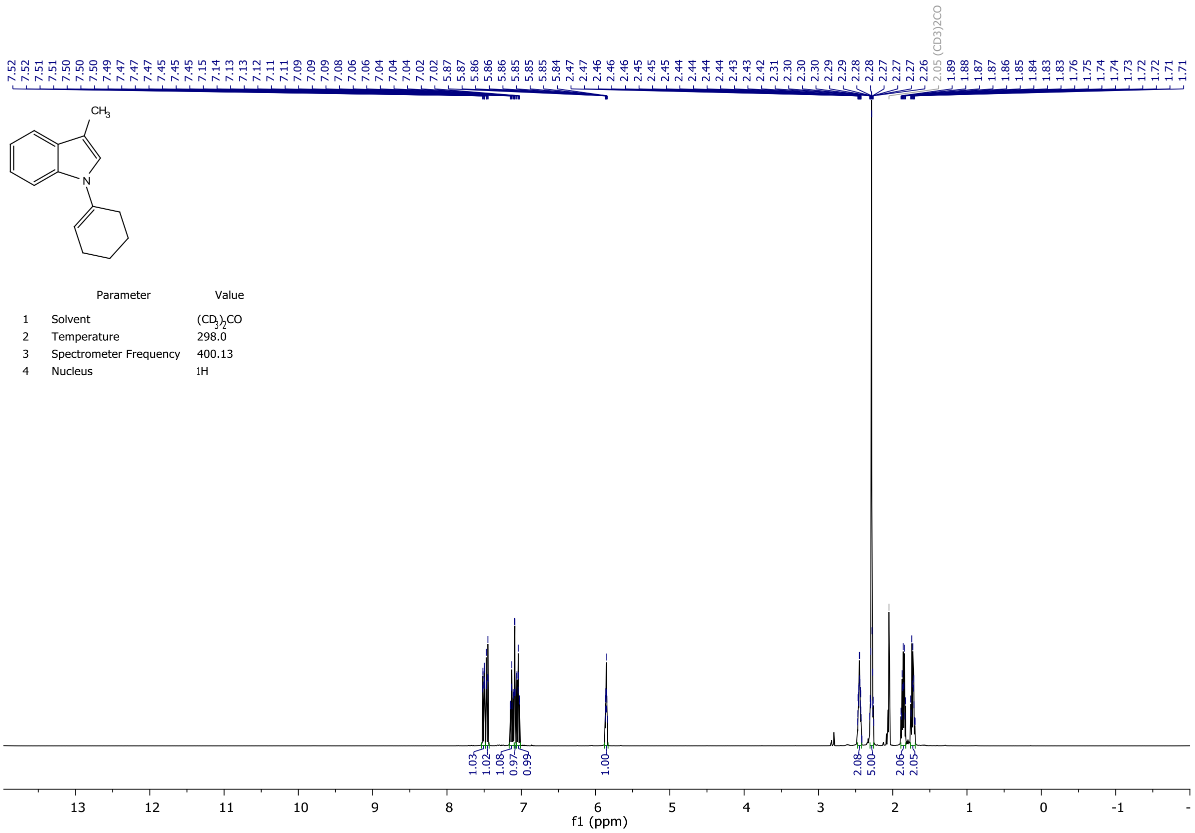

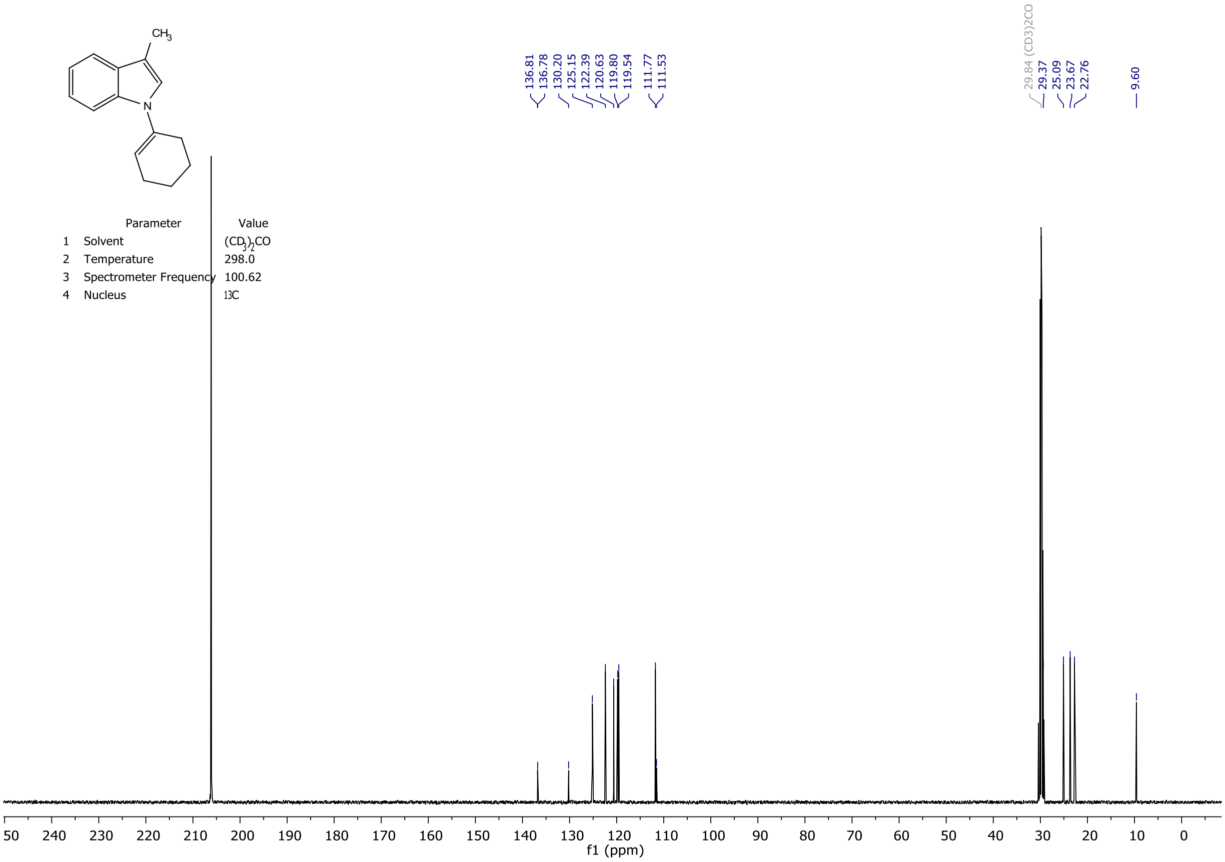

Methyl 2-(1-(cyclohex-1-en-1-yl)-1H-indol-3-yl)acetate, 3b

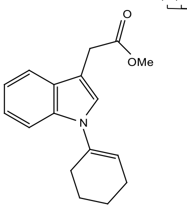

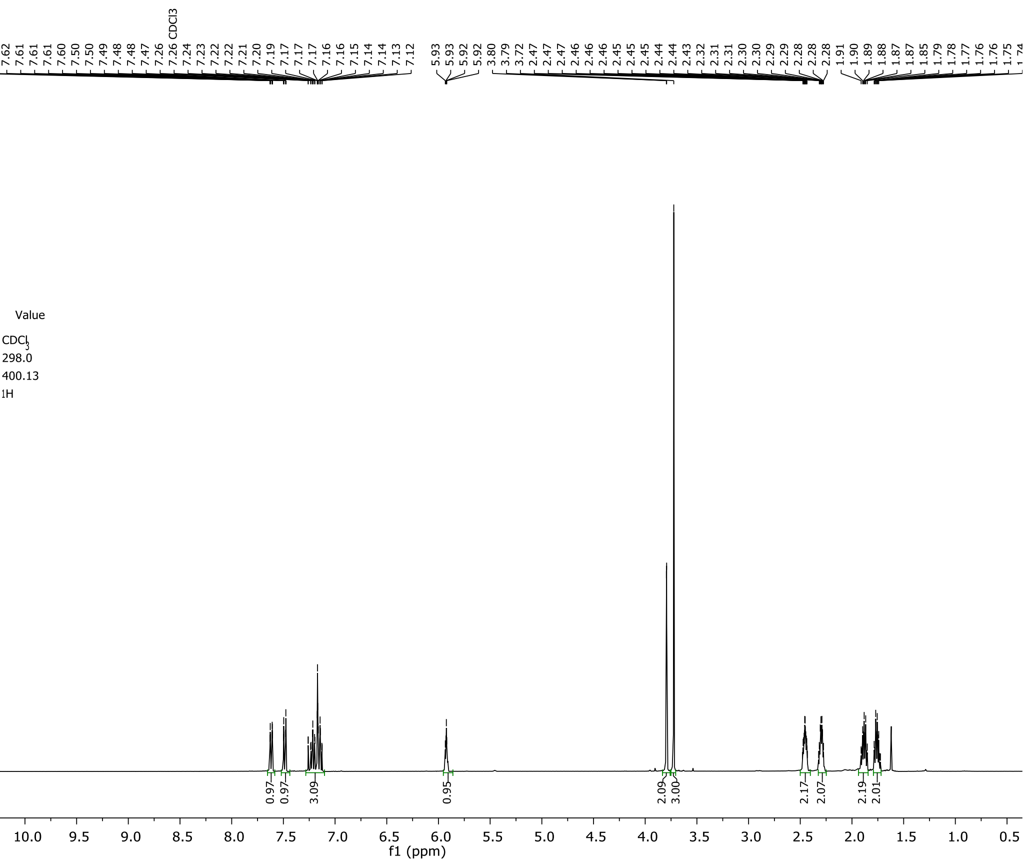

	Parameter	Value
1	Solvent	$CDCl_3$
2	Temperature	298.0
3	Spectrometer Frequency	400.13
4	Nucleus	^1H

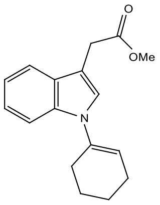

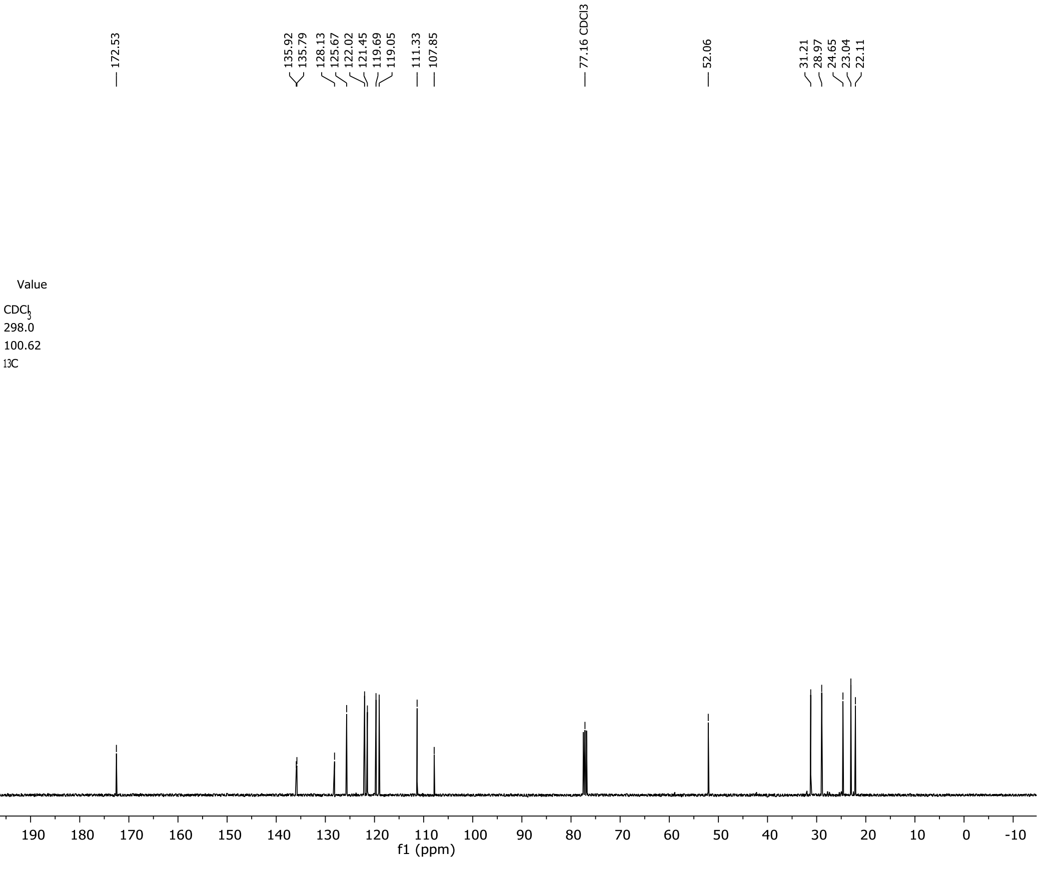

	Parameter	Value
1	Solvent	$CDCl_3$
2	Temperature	298.0
3	Spectrometer Frequency	100.62
4	Nucleus	^{13}C

1-(cyclohex-1-en-1-yl)-1H-pyrazole, 3c CAS 25834-38-2[1]

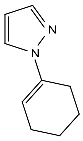

	Parameter	Value
1	Solvent	$(CD_3)_2CO$
2	Temperature	298.0
3	Spectrometer Frequency	400.13
4	Nucleus	1H

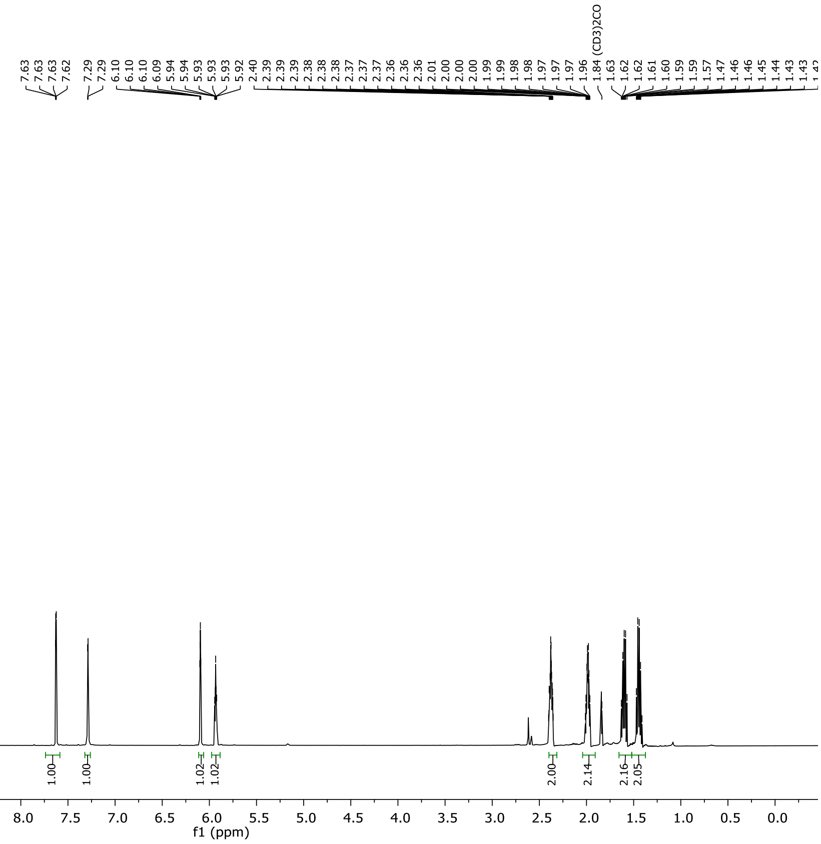

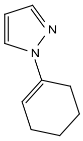

	Parameter	Value
1	Solvent	$(CD_3)_2CO$
2	Temperature	298.0
3	Spectrometer Frequency	100.62
4	Nucleus	13C

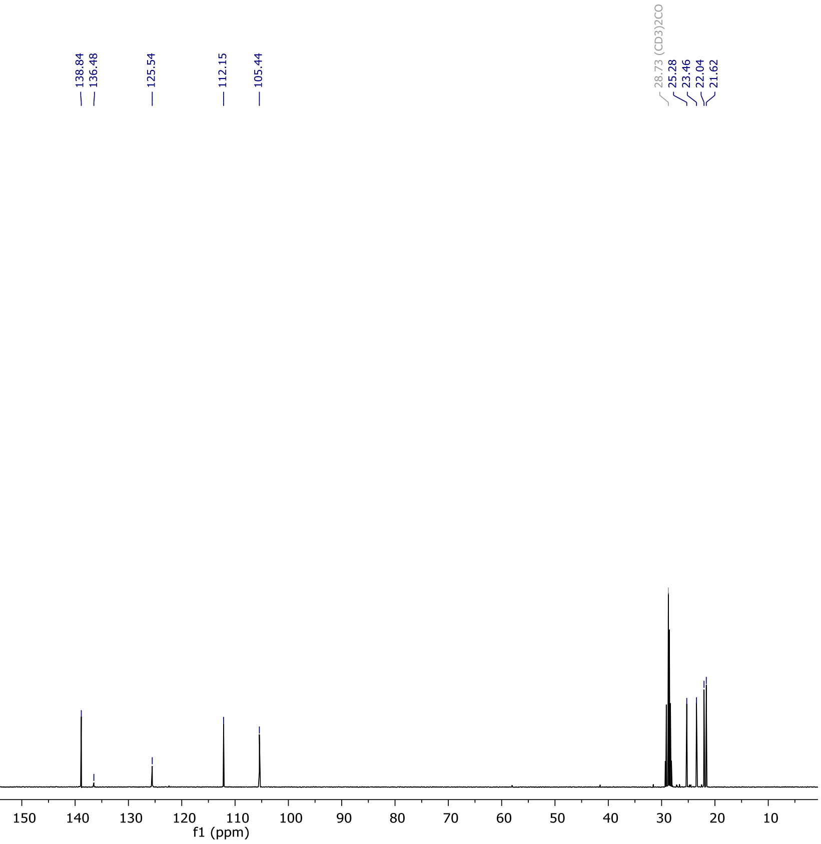

1-(cyclohex-1-en-1-yl)-1H-pyrazole, 3d

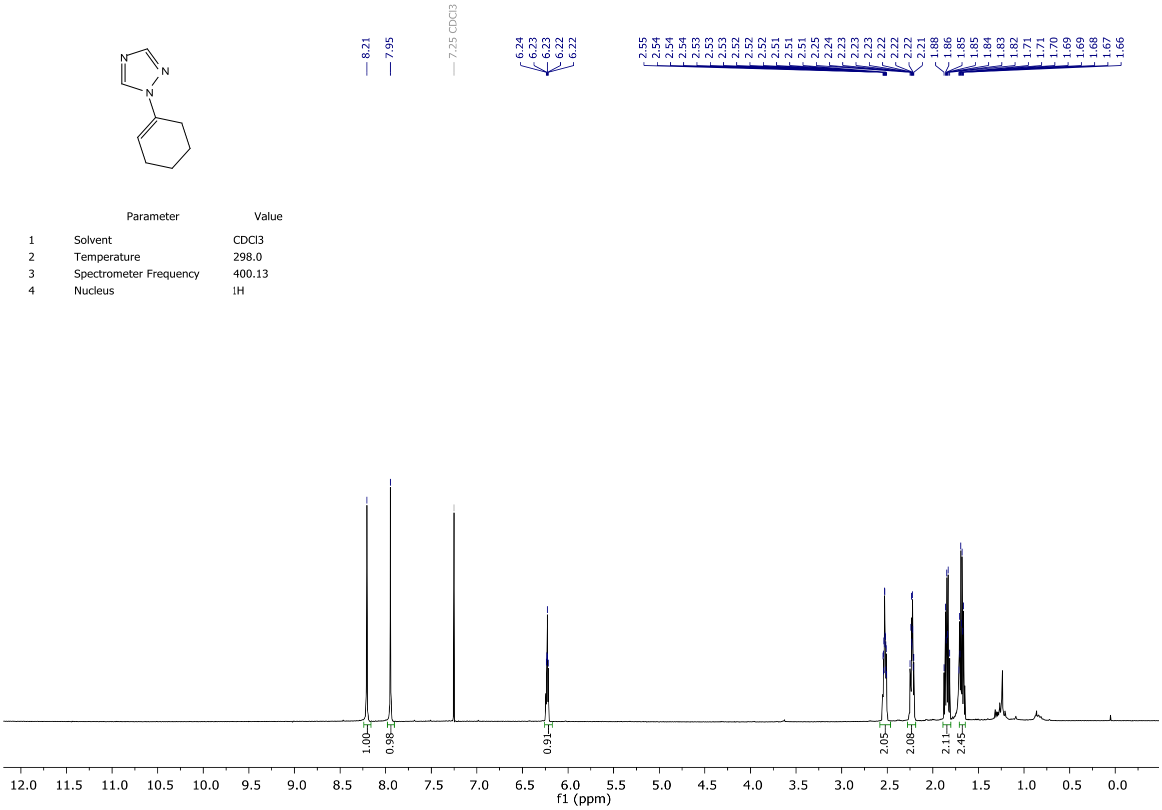

	Parameter	Value
1	Solvent	CDCl3
2	Temperature	298.0
3	Spectrometer Frequency	400.13
4	Nucleus	1H

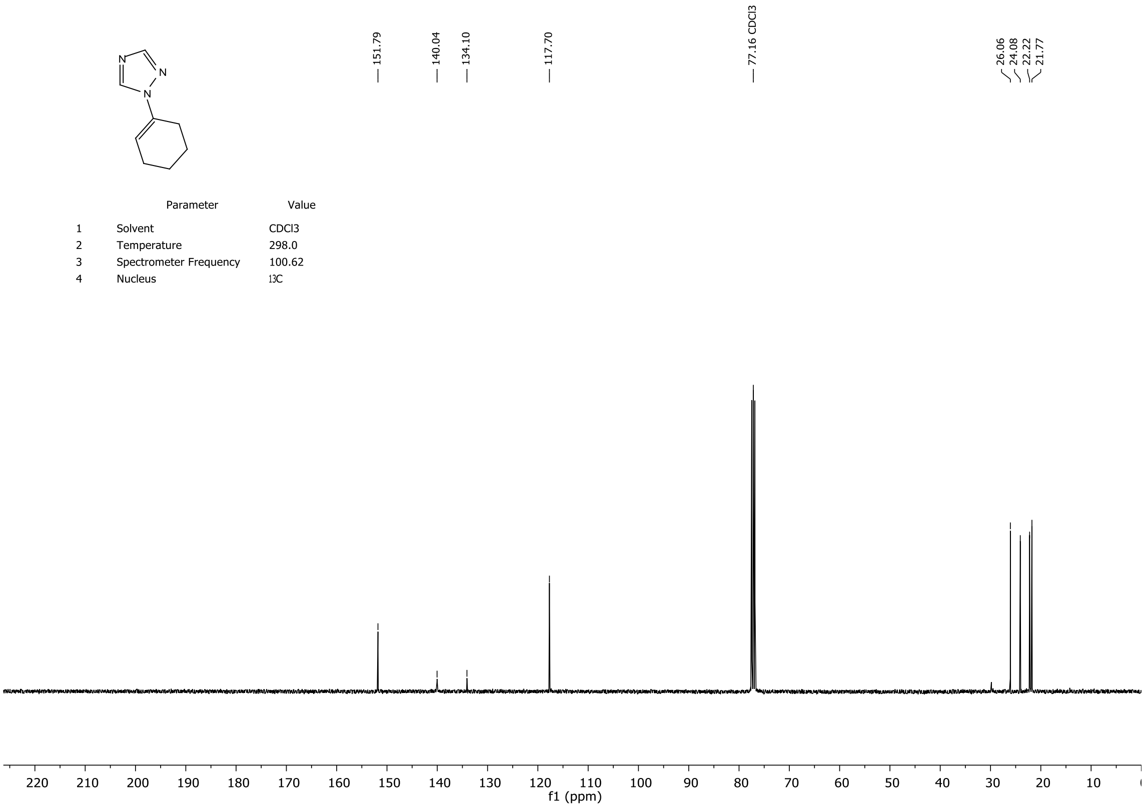

	Parameter	Value
1	Solvent	CDCl3
2	Temperature	298.0
3	Spectrometer Frequency	100.62
4	Nucleus	13C

1-(cyclohex-1-en-1-yl)-1H-imidazole, 3e CAS 74198-41-0[1]

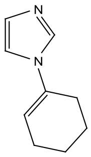

	Parameter	Value
1	Solvent	$CDCl_3$
2	Temperature	298.0
3	Spectrometer Frequency	400.13
4	Nucleus	1H

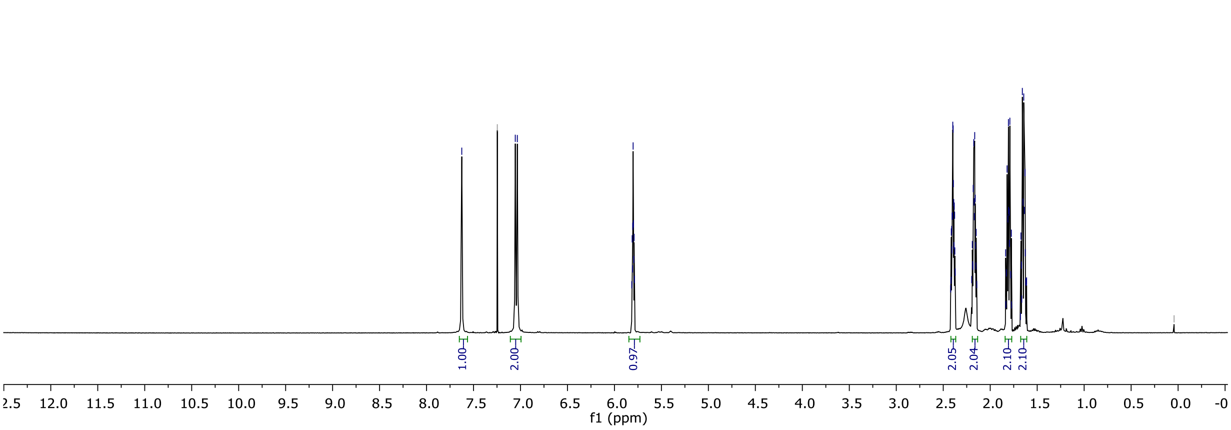

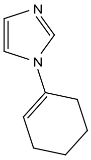

	Parameter	Value
1	Solvent	$CDCl_3$
2	Temperature	298.0
3	Spectrometer Frequency	100.62
4	Nucleus	13C

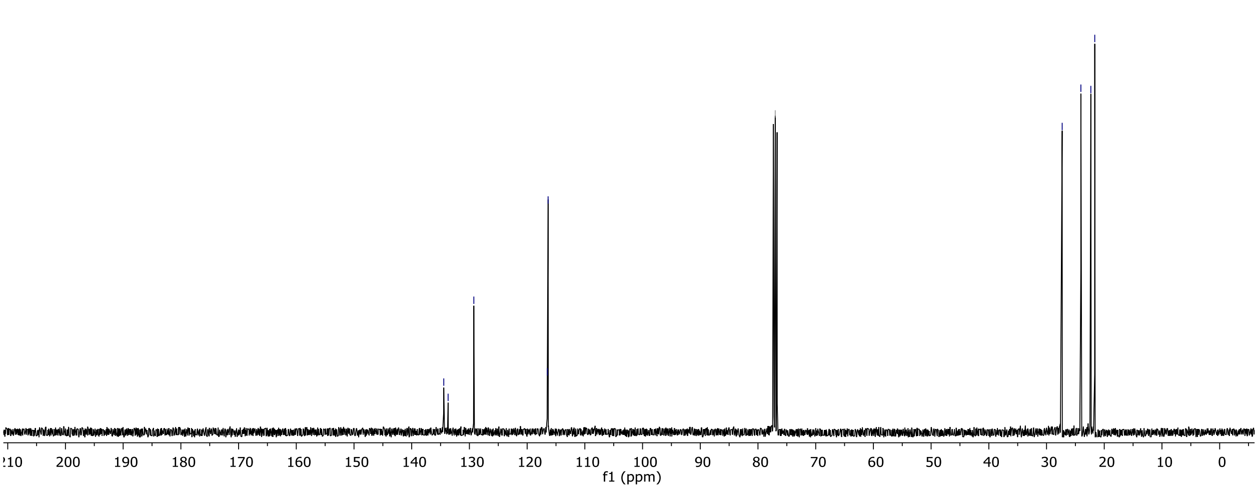

1-(cyclohex-1-en-1-yl)-1H-indazole, 3f-1

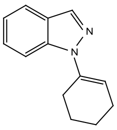

	Parameter	Value
1	Solvent	(CDCl$_3$
2	Temperature	298.0
3	Spectrometer Frequency	400.13
4	Nucleus	1H

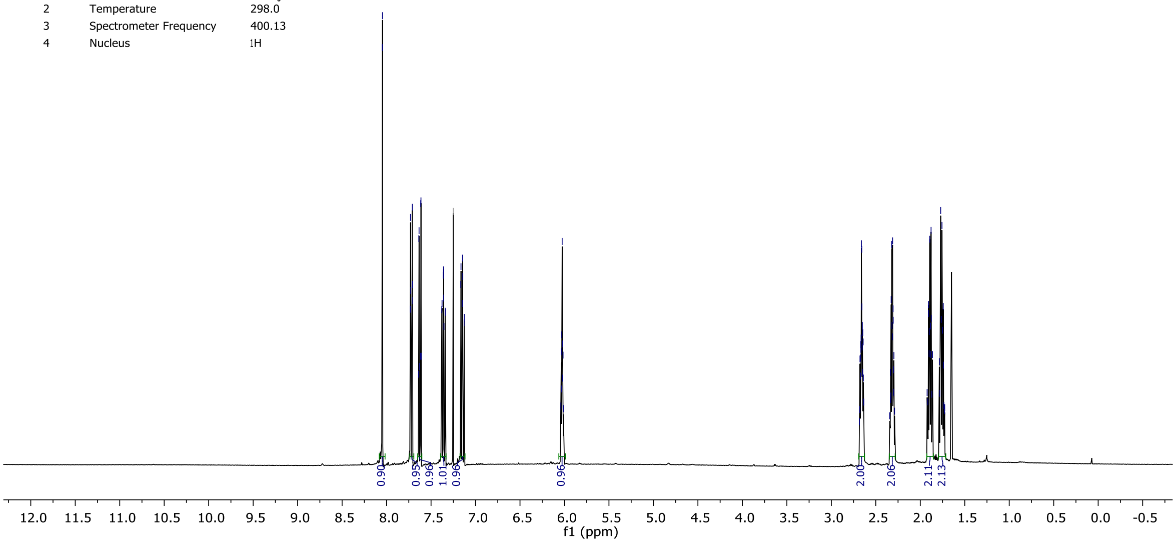

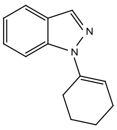

	Parameter	Value
1	Solvent	(CDCl$_3$
2	Temperature	298.0
3	Spectrometer Frequency	100.62
4	Nucleus	13C

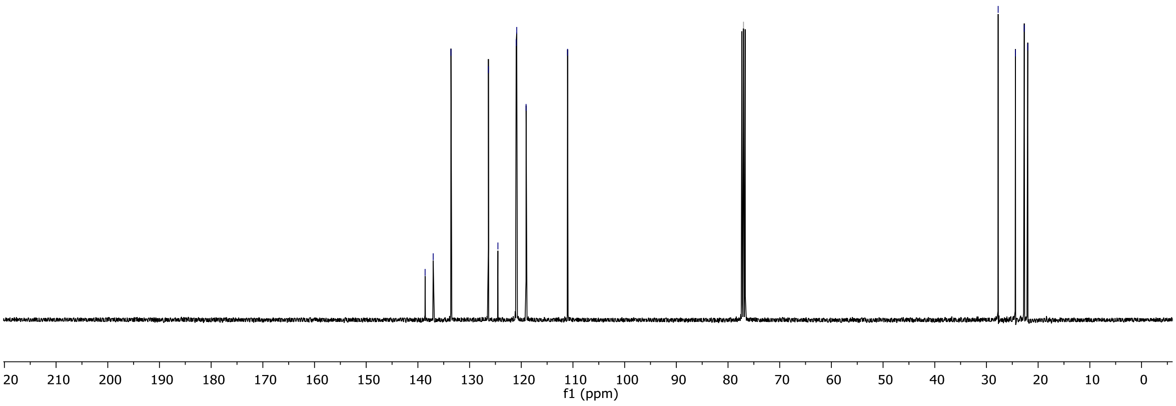

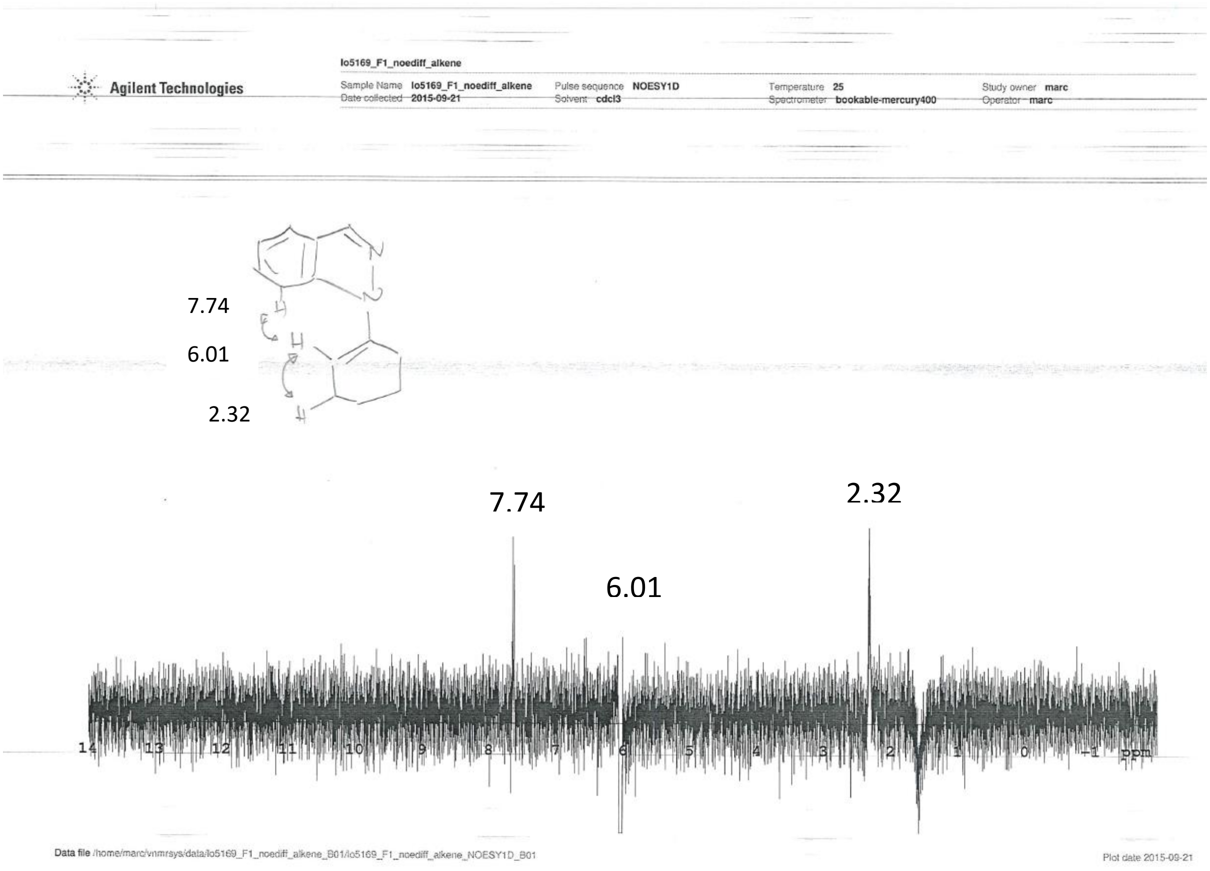
lo5169_F1_noediff_alkene
Agilent Technologies
Sample Name lo5169_F1_noediff_alkene
Date collected 2015-09-21
Pulse sequence NOESY1D
Solvent cdcl3
Temperature 25
Spectrometer bookable-mercury400
Study owner marc
Operator marc
7.74
6.01
2.32
7.74
6.01
2.32
ppm
Data file /home/marc/vnmrsys/data/lo5169_F1_noediff_alkene_B01/lo5169_F1_noediff_alkene_NOESY1D_B01
Plot date 2015-09-21

2-(Cyclohex-1-en-1-yl)-2H-indazole, 3f-2

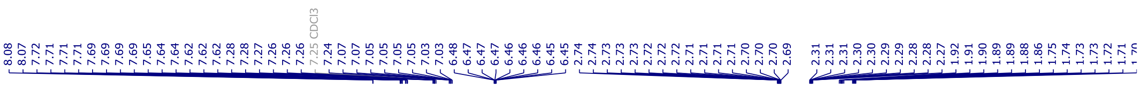

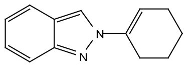

	Parameter	Value
1	Solvent	$(CDCl_3$
2	Temperature	298.0
3	Spectrometer Frequency	400.13
4	Nucleus	1H

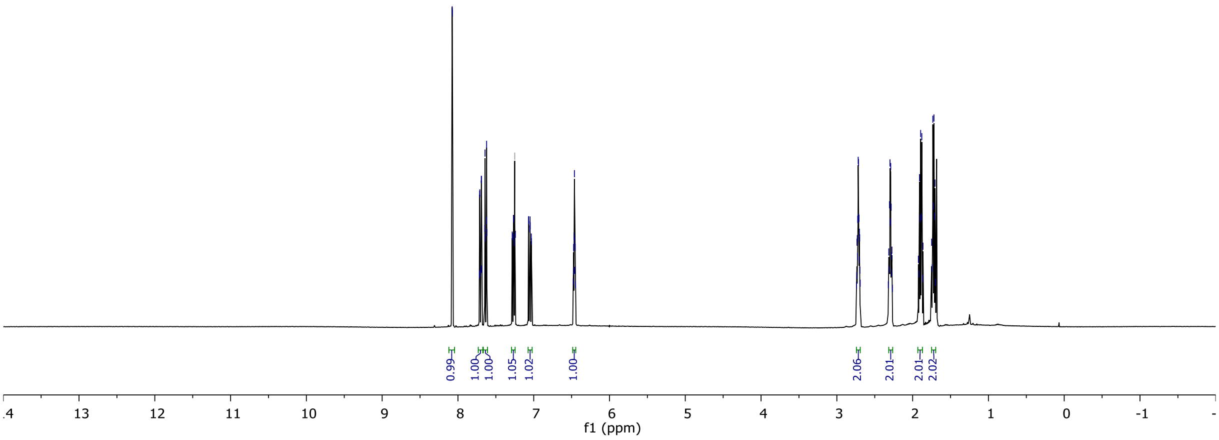

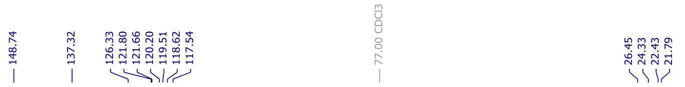

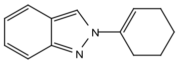

	Parameter	Value
1	Solvent	$(CDCl_3$
2	Temperature	298.0
3	Spectrometer Frequency	100.62
4	Nucleus	13C

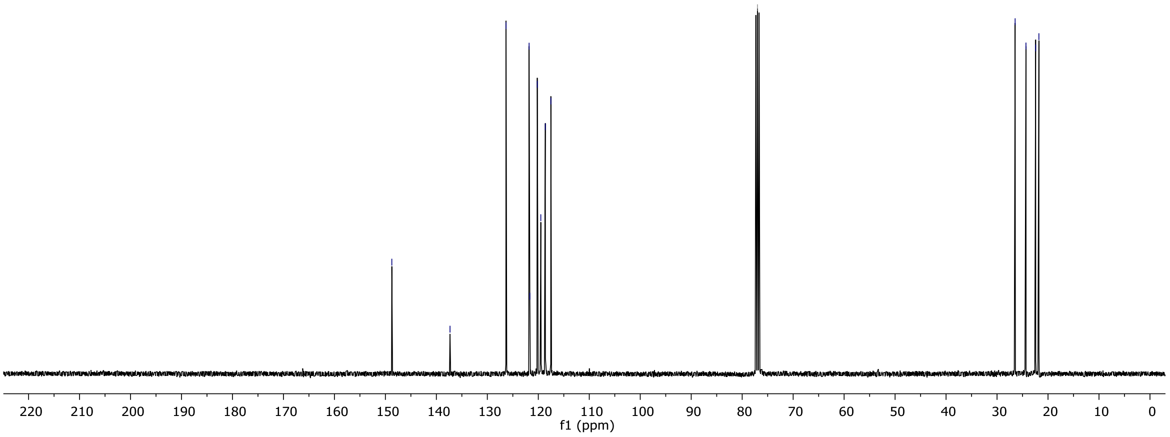

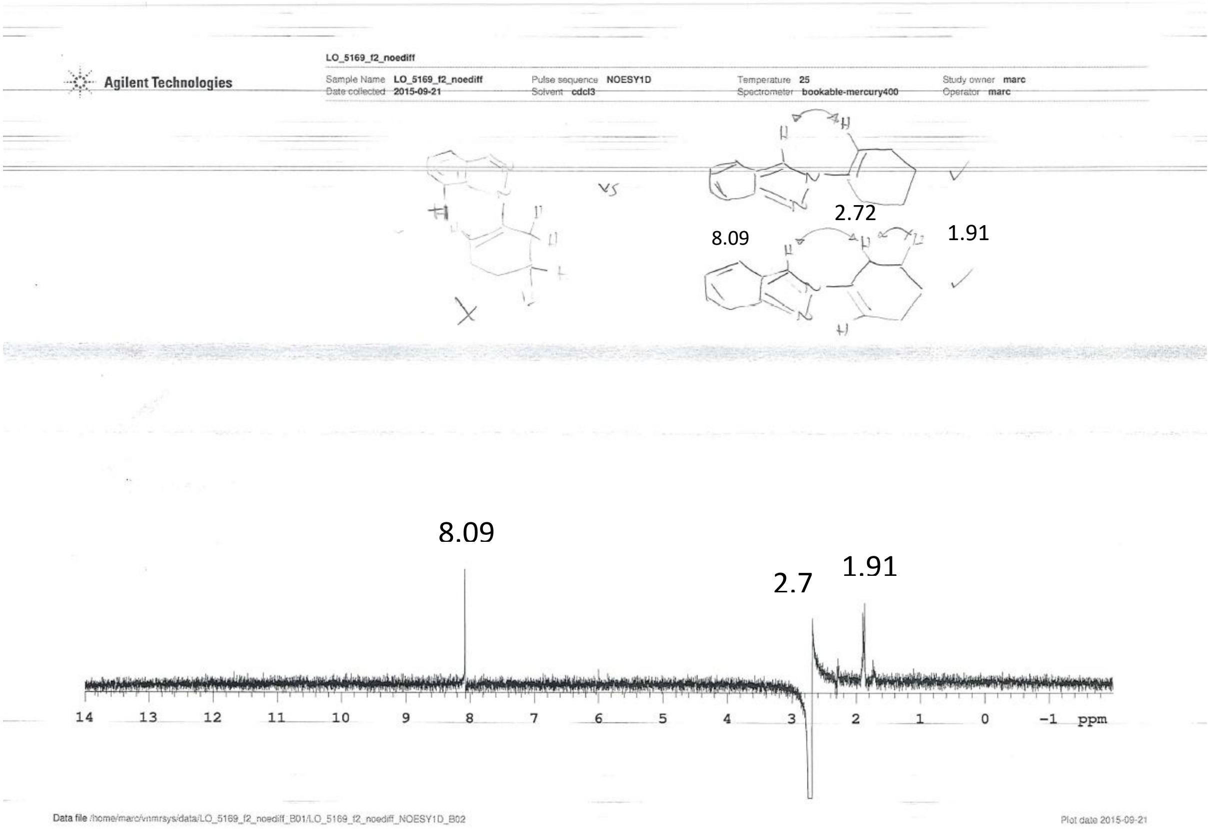
LO_5169_f2_noediff
Agilent Technologies
Sample Name LO_5169_f2_noediff
Date collected 2015-09-21
Pulse sequence NOESY1D
Solvent cdcl3
Temperature 25
Spectrometer bookable-mercury400
Study owner marc
Operator marc
vs
2.72
8.09
1.91
8.09
2.7
1.91
14 13 12 11 10 9 8 7 6 5 4 3 2 1 0 -1 ppm
Data file /home/marc/vnmrsys/data/LO_5169_f2_noediff_B01/LO_5169_f2_noediff_NOESY1D_B02
Plot date 2015-09-21

1-(cyclohex-1-en-1-yl)-1H-benzo[d][1,2,3]triazole , 3g-1 CAS 73006-66-3[2]

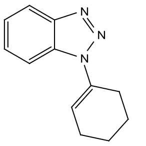

	Parameter	Value
1	Solvent	$(CD_3)_2CO$
2	Temperature	298.0
3	Spectrometer Frequency	400.13
4	Nucleus	1H

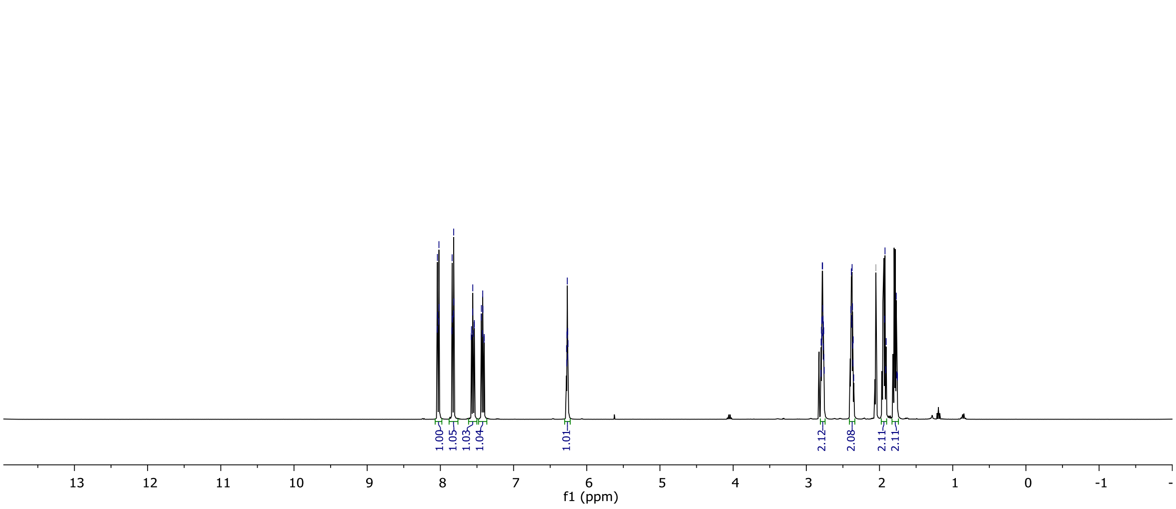

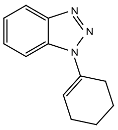

	Parameter	Value
1	Solvent	$(CD_3)_2CO$
2	Temperature	298.0
3	Spectrometer Frequency	100.62
4	Nucleus	13C

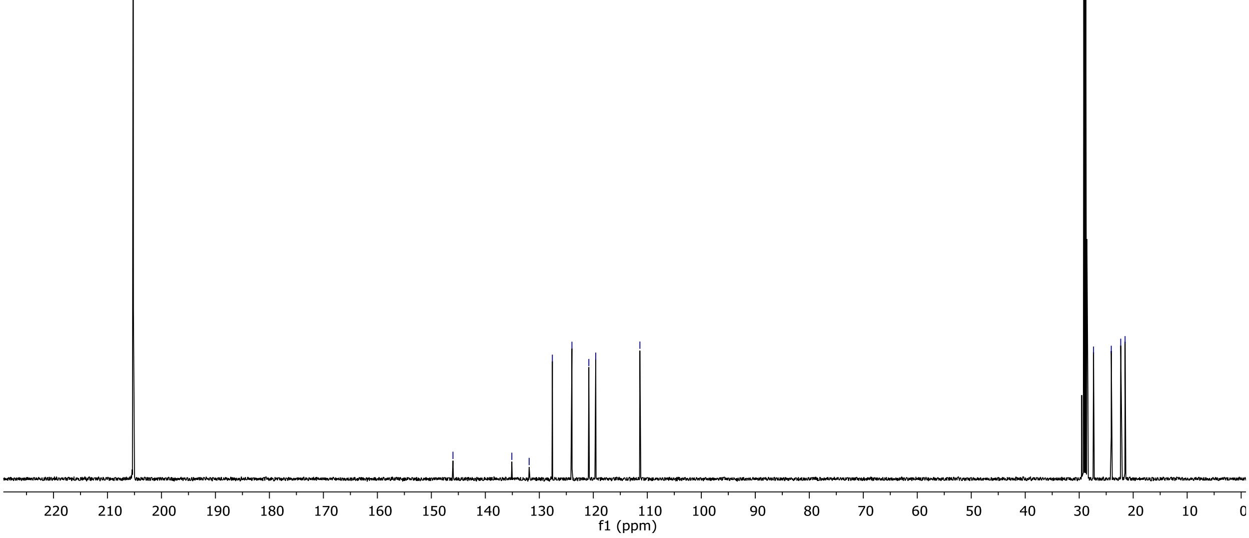

1-(cyclohex-1-en-1-yl)-2H-benzo[d][1,2,3]triazole, 3g-2 CAS 2414619-05-7[2]

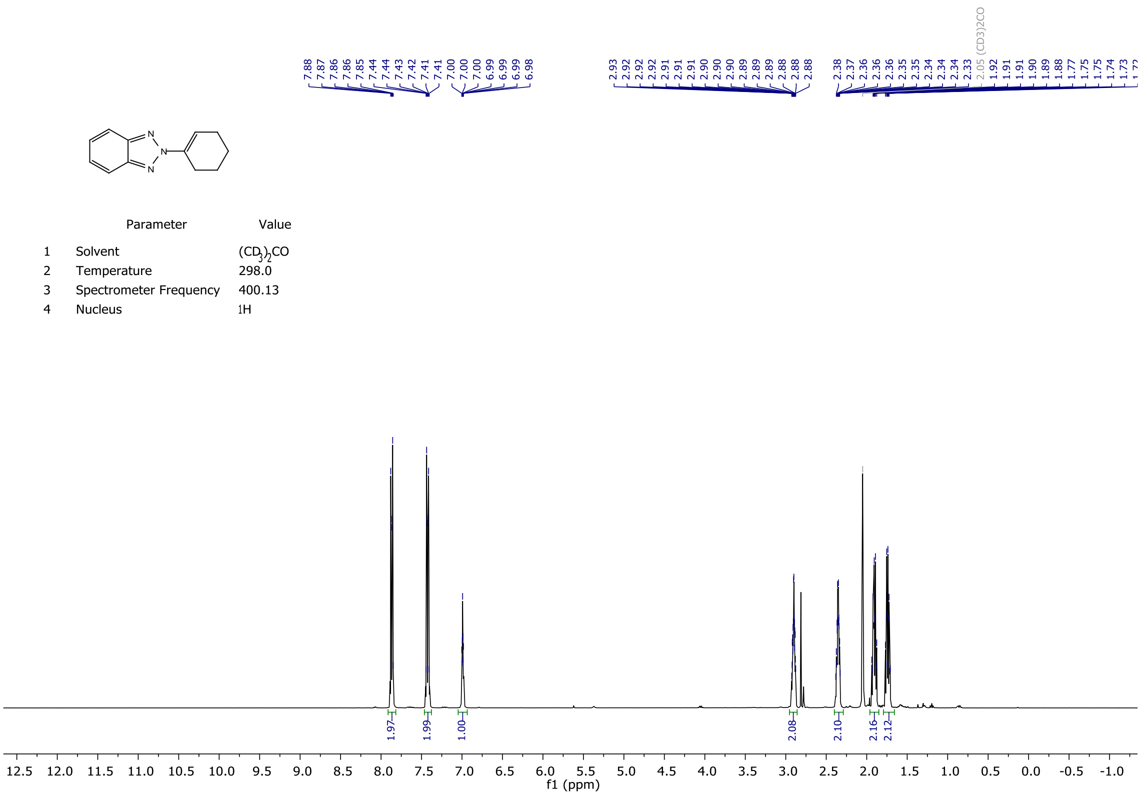

	Parameter	Value
1	Solvent	$(CD_3)_2CO$
2	Temperature	298.0
3	Spectrometer Frequency	400.13
4	Nucleus	^1H

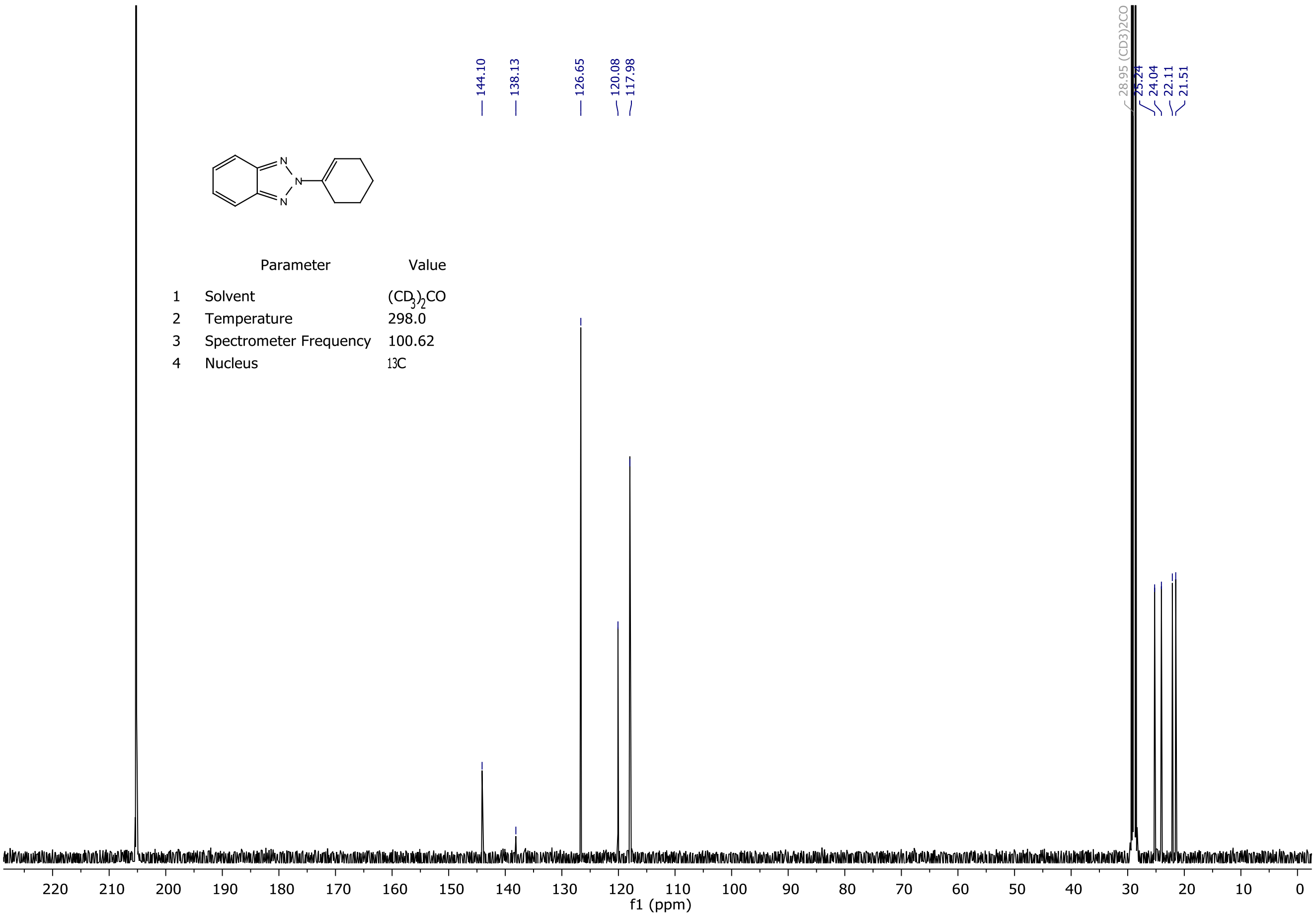

	Parameter	Value
1	Solvent	$(CD_3)_2CO$
2	Temperature	298.0
3	Spectrometer Frequency	100.62
4	Nucleus	^{13}C

1-(cyclohex-1-en-1-yl)-1H-benzo[d]imidazole, 3h CAS 1451090-71-3[3]

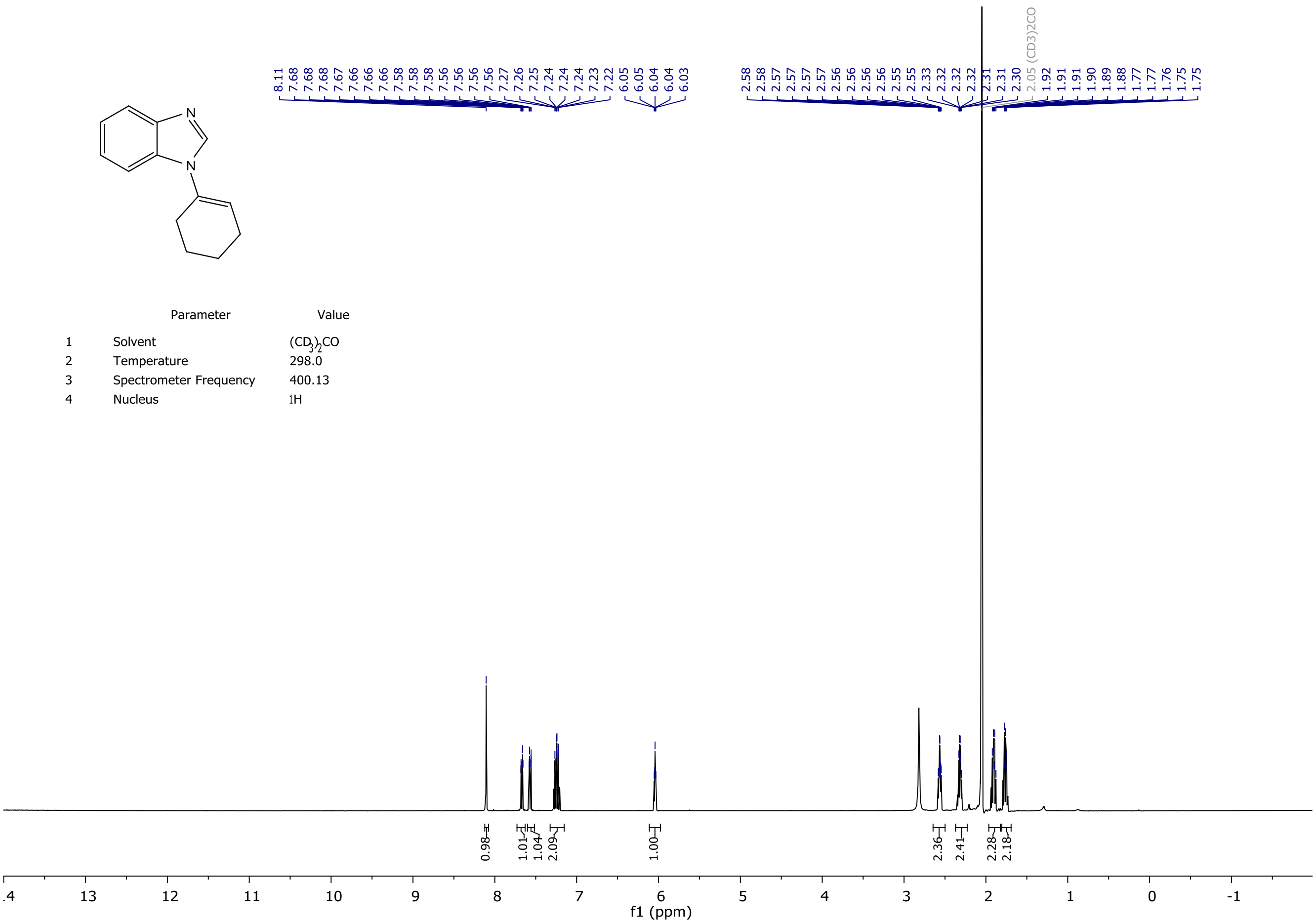

	Parameter	Value
1	Solvent	$(CD_3)_2CO$
2	Temperature	298.0
3	Spectrometer Frequency	400.13
4	Nucleus	1H

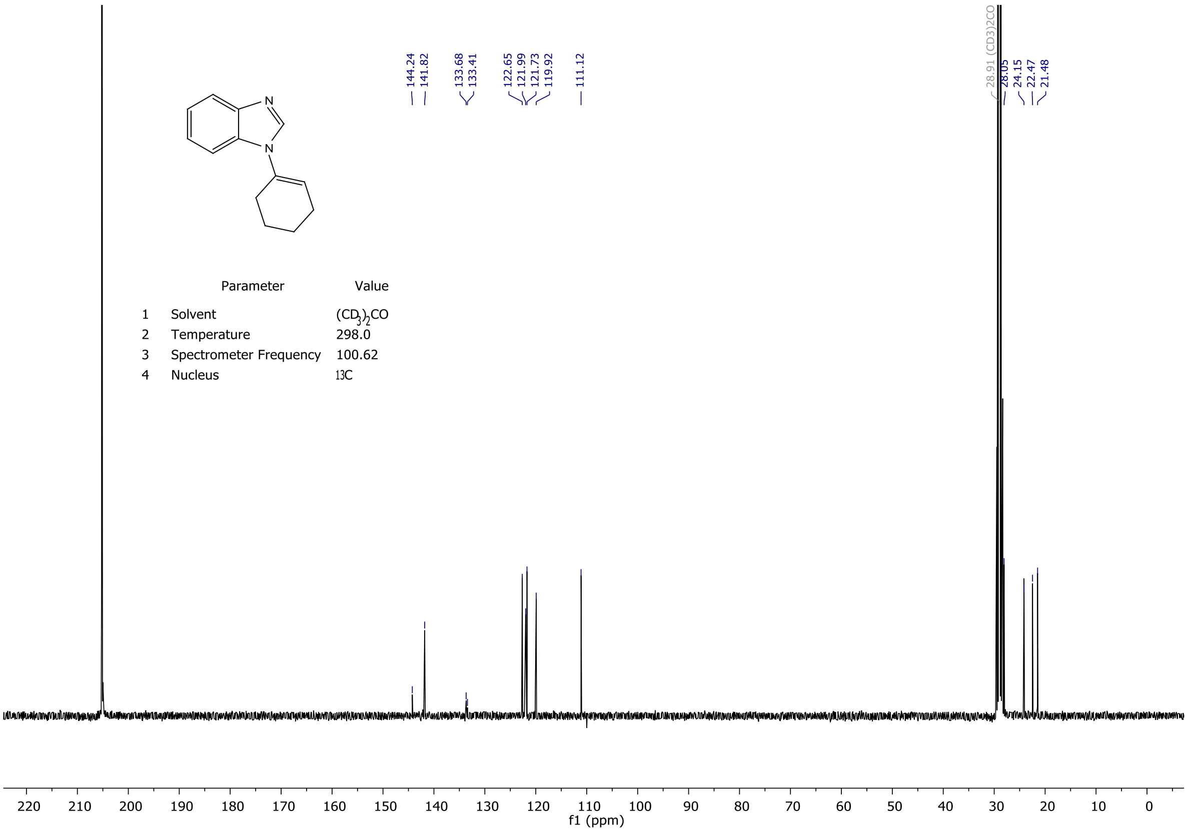

	Parameter	Value
1	Solvent	$(CD_3)_2CO$
2	Temperature	298.0
3	Spectrometer Frequency	100.62
4	Nucleus	13C

1-(Cyclohexen-1-yl)-5,6-dimethyl-benzimidazole, 3i

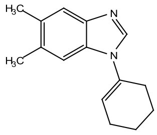

	Parameter	Value
1	Solvent	$(CD_3)_2CO$
2	Temperature	298.0
3	Spectrometer Frequency	400.13
4	Nucleus	1H

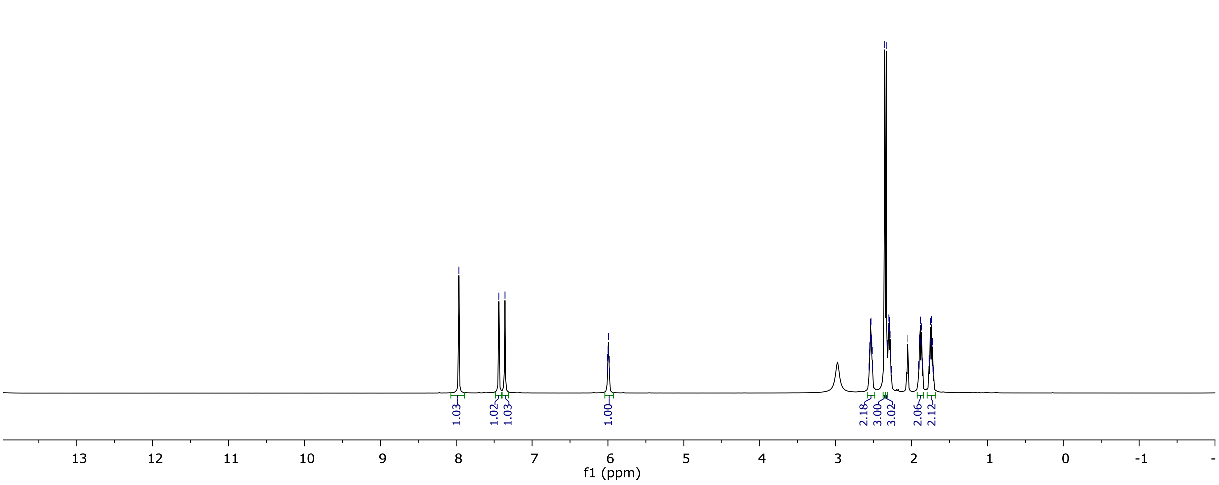

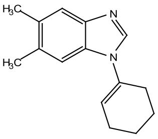

	Parameter	Value
1	Solvent	$(CD_3)_2CO$
2	Temperature	298.0
3	Spectrometer Frequency	100.62
4	Nucleus	13C

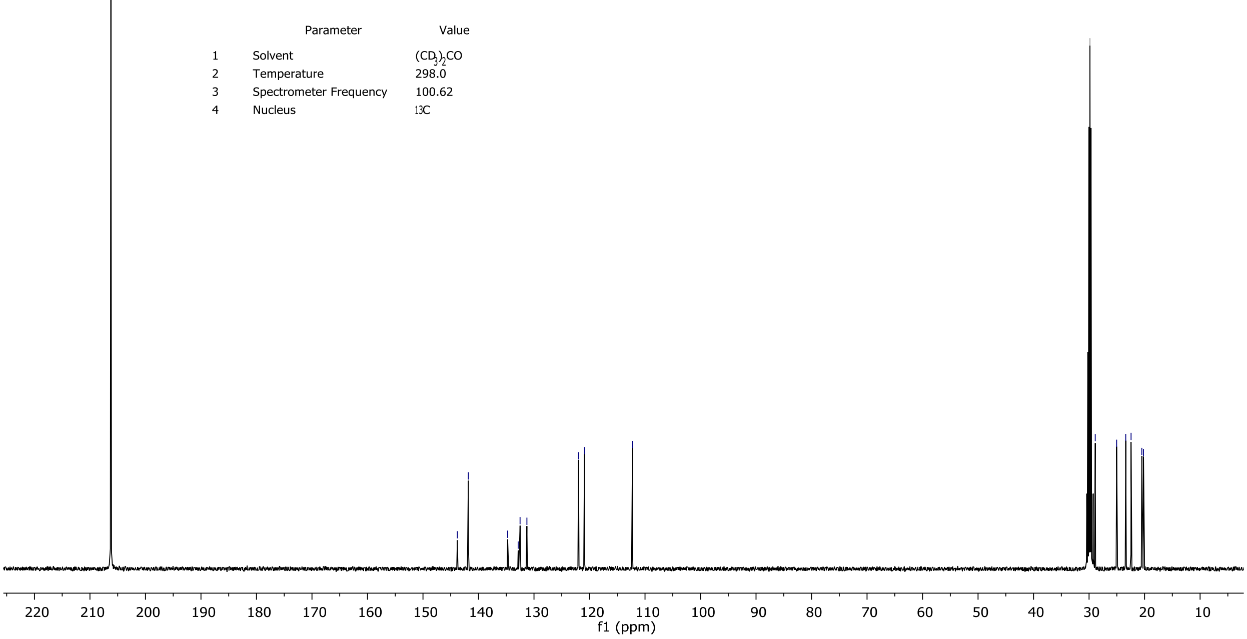

1-(cyclohex-1-en-1-yl)-5-(trifluoromethyl)-1H-benzo[d]imidazole,3j-1

BZ9485_FCC_fR12-12.1.fid

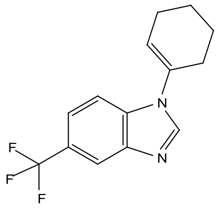

	Parameter	Value
1	Solvent	$(CD_3)_2CO$
2	Temperature	298.0
3	Spectrometer Frequency	400.13
4	Nucleus	1H

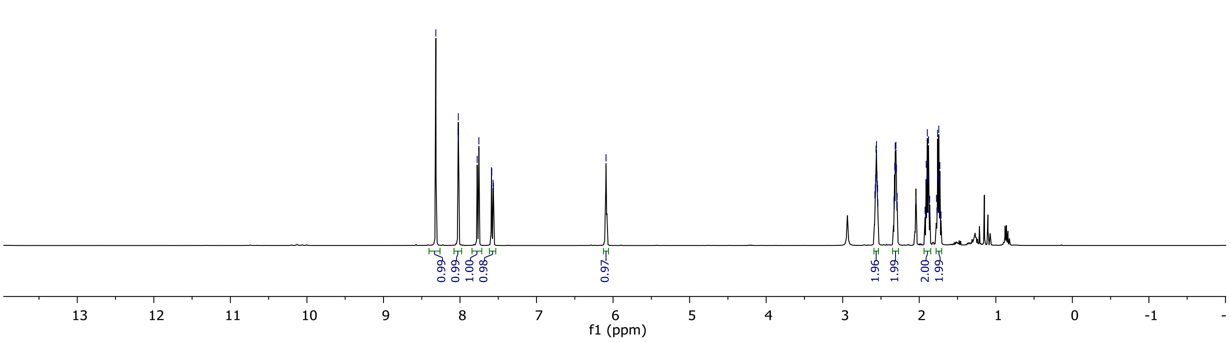

BZ9485_FCC_fR12-12.2.fid

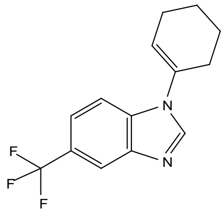

	Parameter	Value
1	Solvent	$(CD_3)_2CO$
2	Temperature	298.0
3	Spectrometer Frequency	100.62
4	Nucleus	13C

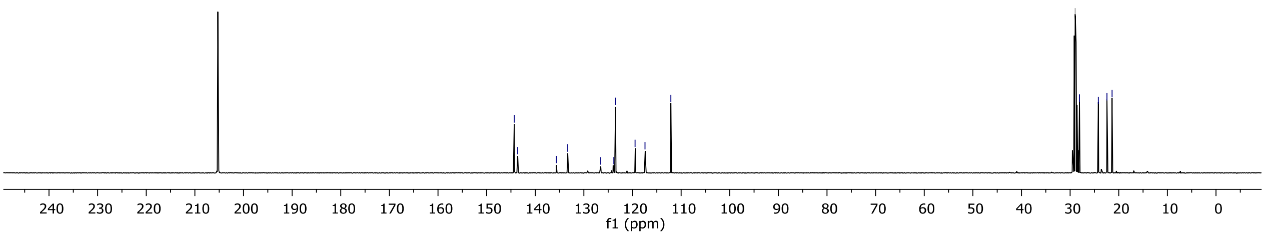

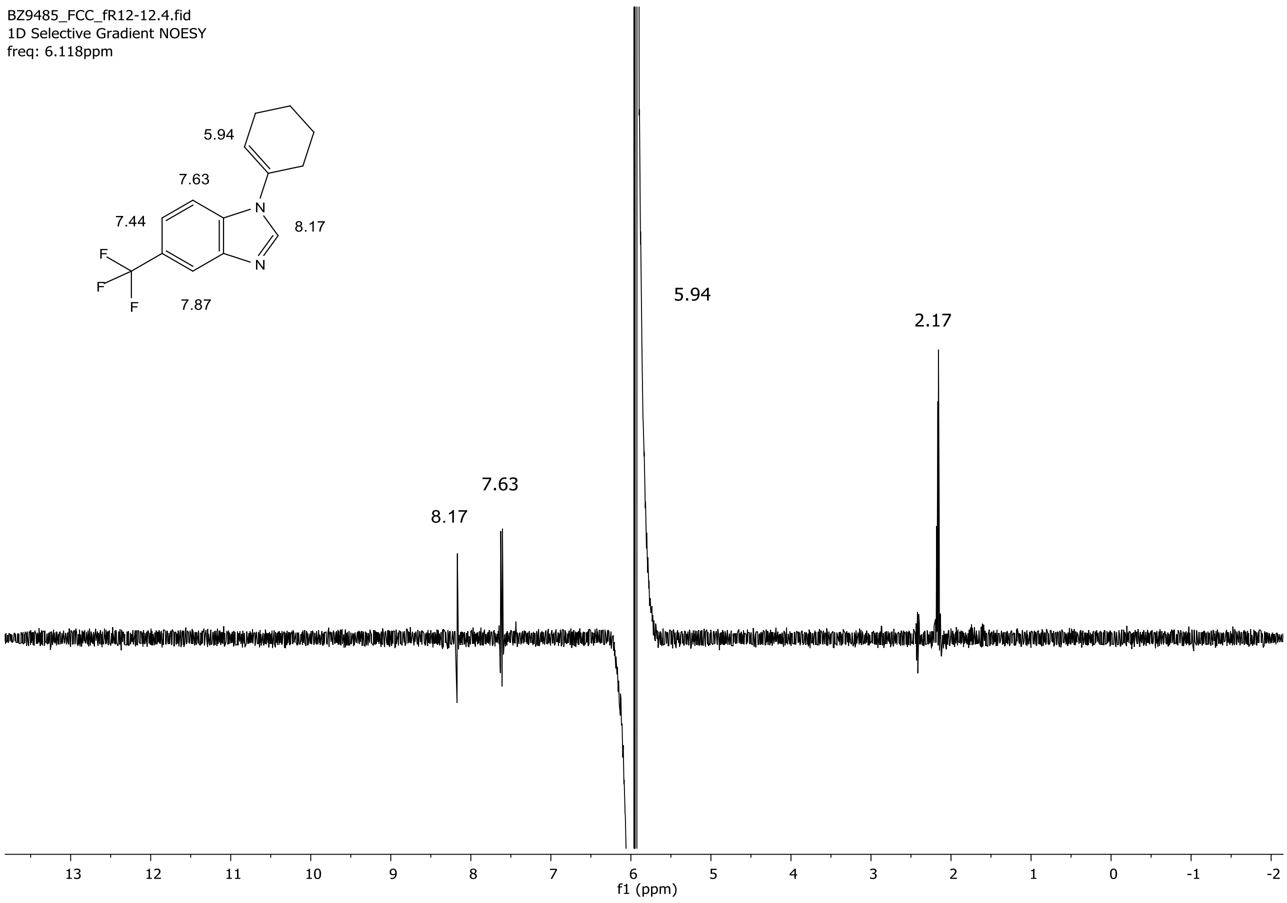
BZ9485_FCC_fR12-12.4.fid
1D Selective Gradient NOESY
freq: 6.118ppm
5.94
7.63
7.44
8.17
7.87
N
N
F
F
F
5.94
2.17
7.63
8.17
13
12
11
10
9
8
7
6
5
4
3
2
1
0
-1
-2
f1 (ppm)

1-(cyclohex-1-en-1-yl)-6-(trifluoromethyl)-1H-benzo[d]imidazole 3j-2

BZ9485_FCC_fr13-17.6.fid

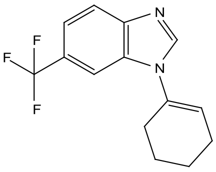

	Parameter	Value
1	Solvent	$(CD_3)_2CO$
2	Temperature	298.0
3	Spectrometer Frequency	100.62
4	Nucleus	13C

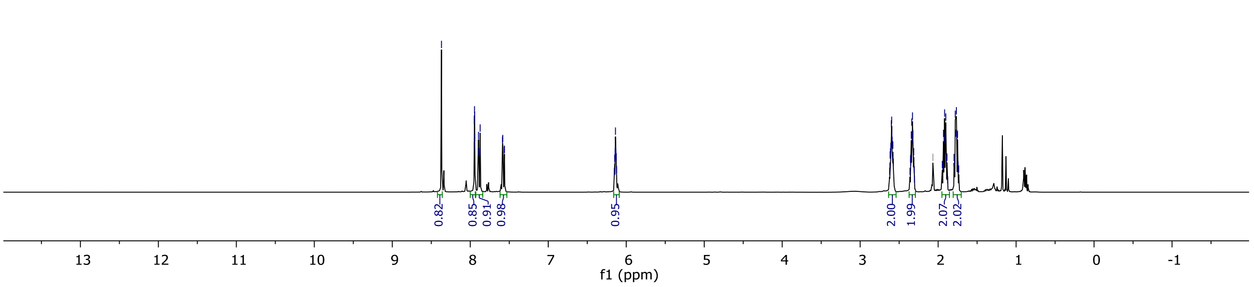

BZ9485_FCC_fR13_17.5.fid

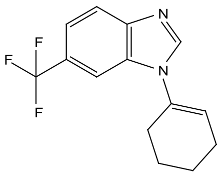

	Parameter	Value
1	Solvent	CD_3Cl
2	Temperature	298.0
3	Spectrometer Frequency	100.62
4	Nucleus	13C

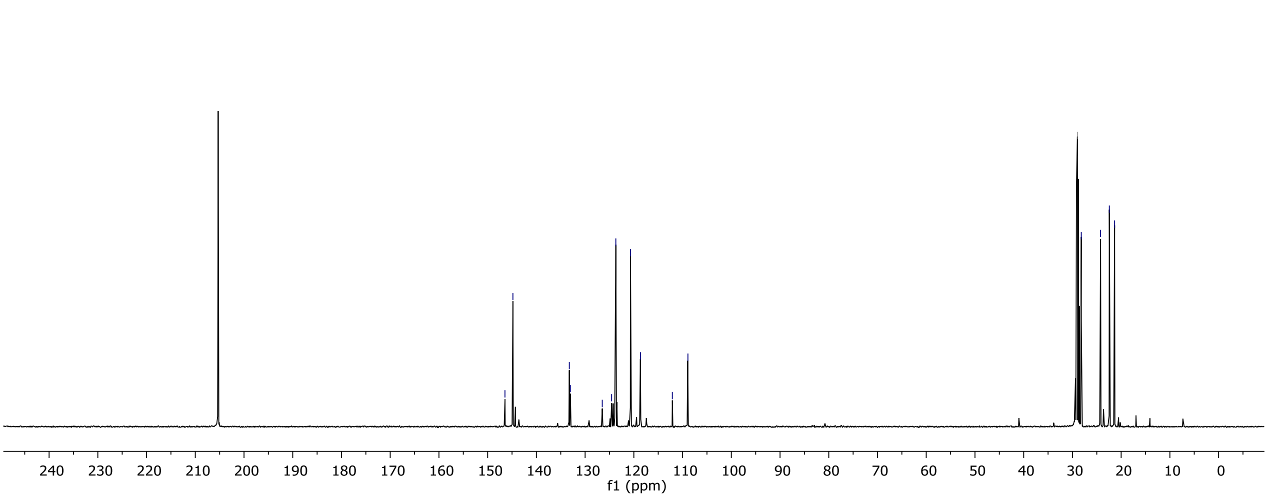

9-(Cyclohex-1-en-1-yl)-9H-carbazole, 3k

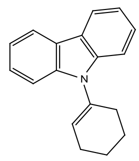

	Parameter	Value
1	Solvent	$(CD_3)_2SO$
2	Temperature	298.0
3	Spectrometer Frequency	400.13
4	Nucleus	^1H

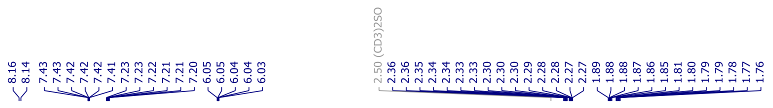

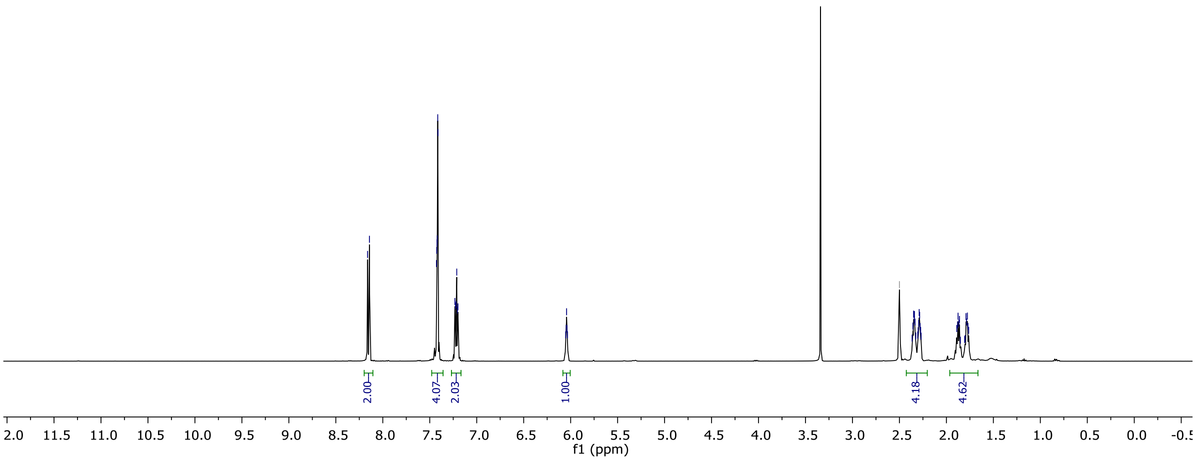

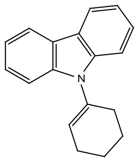

	Parameter	Value
1	Solvent	$(CD_3)_2SO$
2	Temperature	298.0
3	Spectrometer Frequency	100.62
4	Nucleus	^{13}C

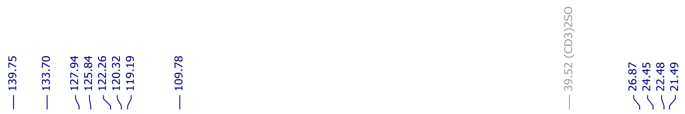

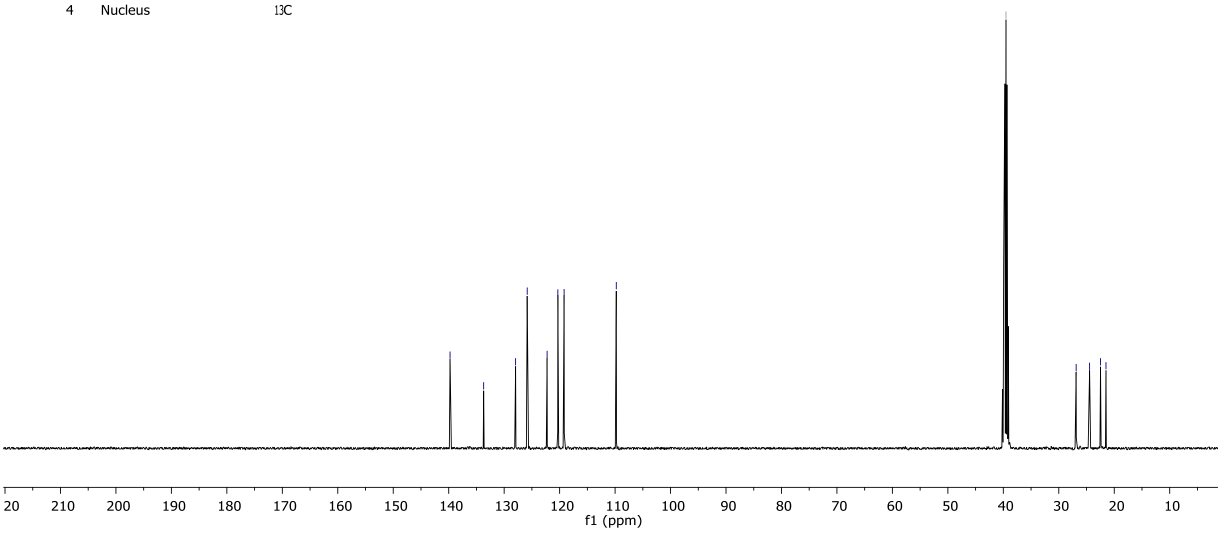

9-(cyclohexen-1-yl)-1,2,3,4-tetrahydrocarbazole, 3l

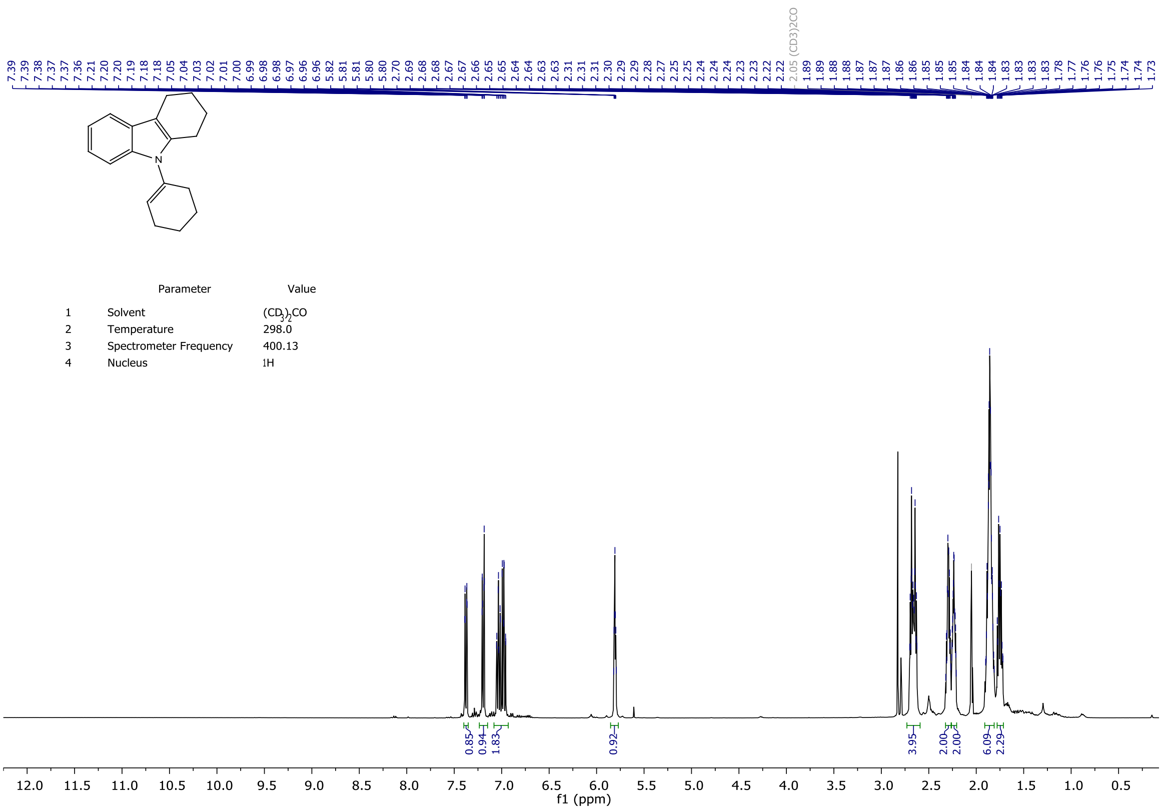

	Parameter	Value
1	Solvent	$(CD_3)_2CO$
2	Temperature	298.0
3	Spectrometer Frequency	400.13
4	Nucleus	1H

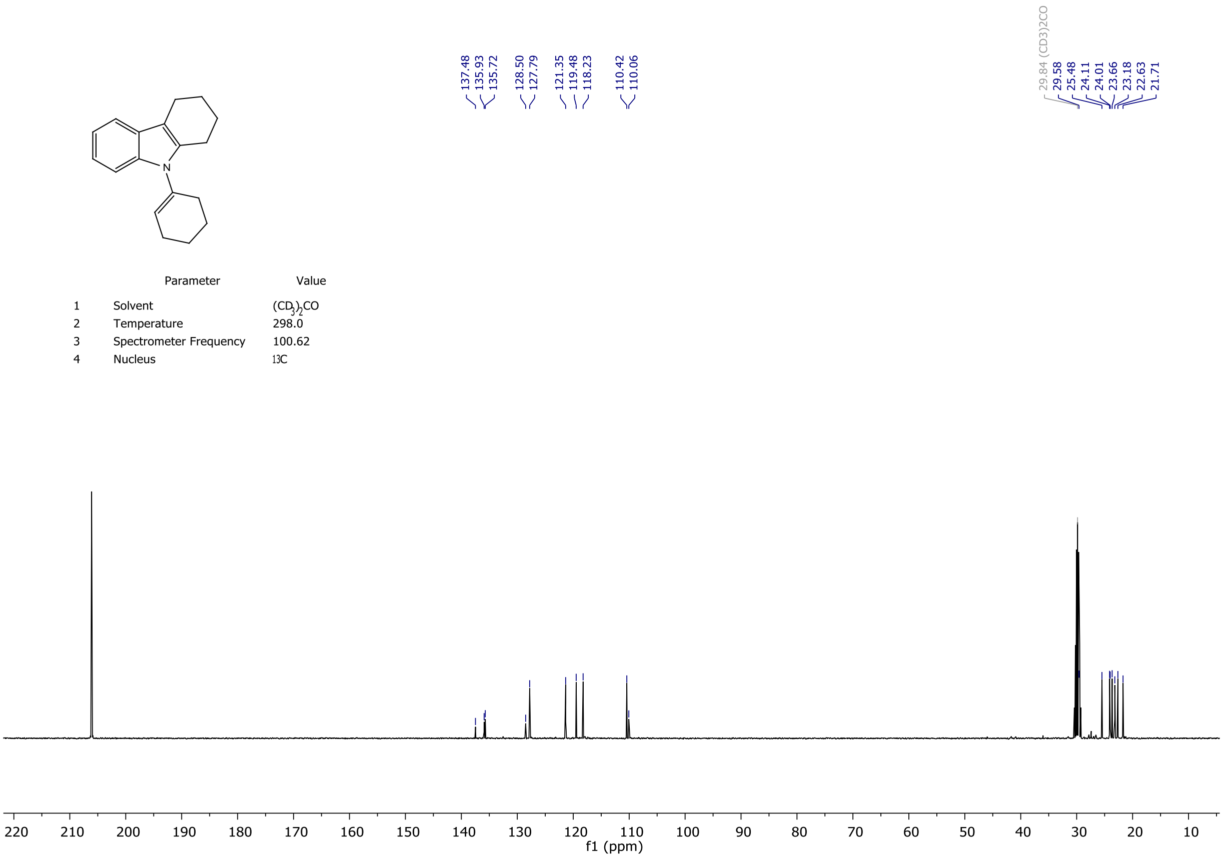

	Parameter	Value
1	Solvent	$(CD_3)_2CO$
2	Temperature	298.0
3	Spectrometer Frequency	100.62
4	Nucleus	13C

10-(cyclohexen-1-yl)phenoxazine, 3m

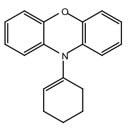

	Parameter	Value
1	Solvent	$CDCl_3$
2	Temperature	298.0
3	Spectrometer Frequency	400.13
4	Nucleus	^{1}H

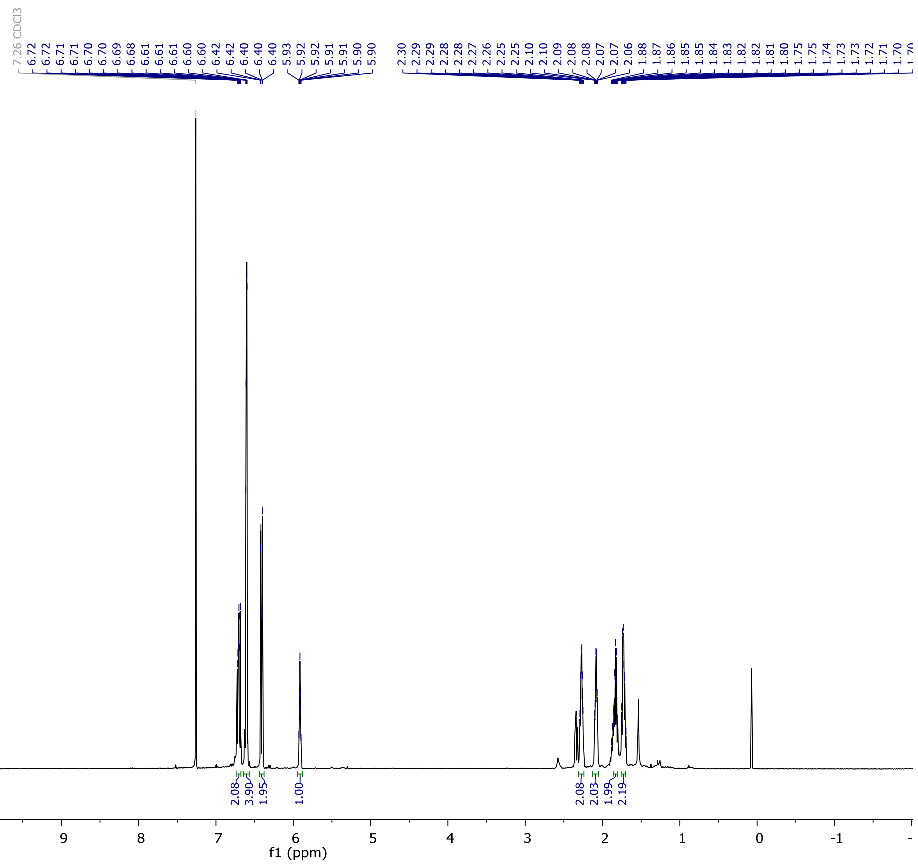

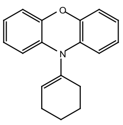

	Parameter	Value
1	Solvent	$CDCl_3$
2	Temperature	298.0
3	Spectrometer Frequency	100.62
4	Nucleus	^{13}C

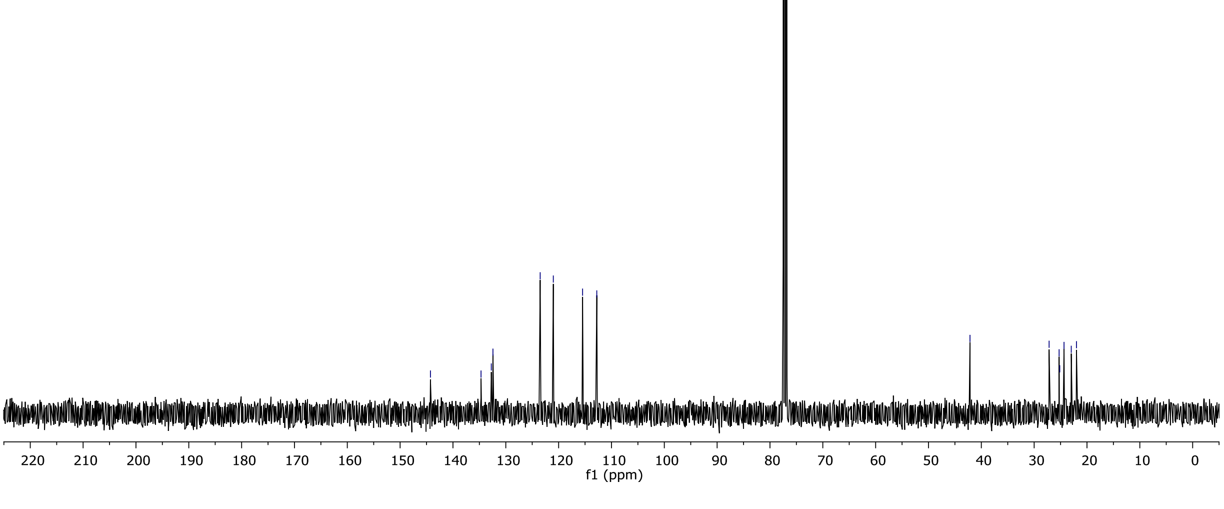

Methyl Nα-(((9H-fluoren-9-yl)methoxy)carbonyl)-1-(cyclohex-1-en-1-yl)tryptophanate, 3n

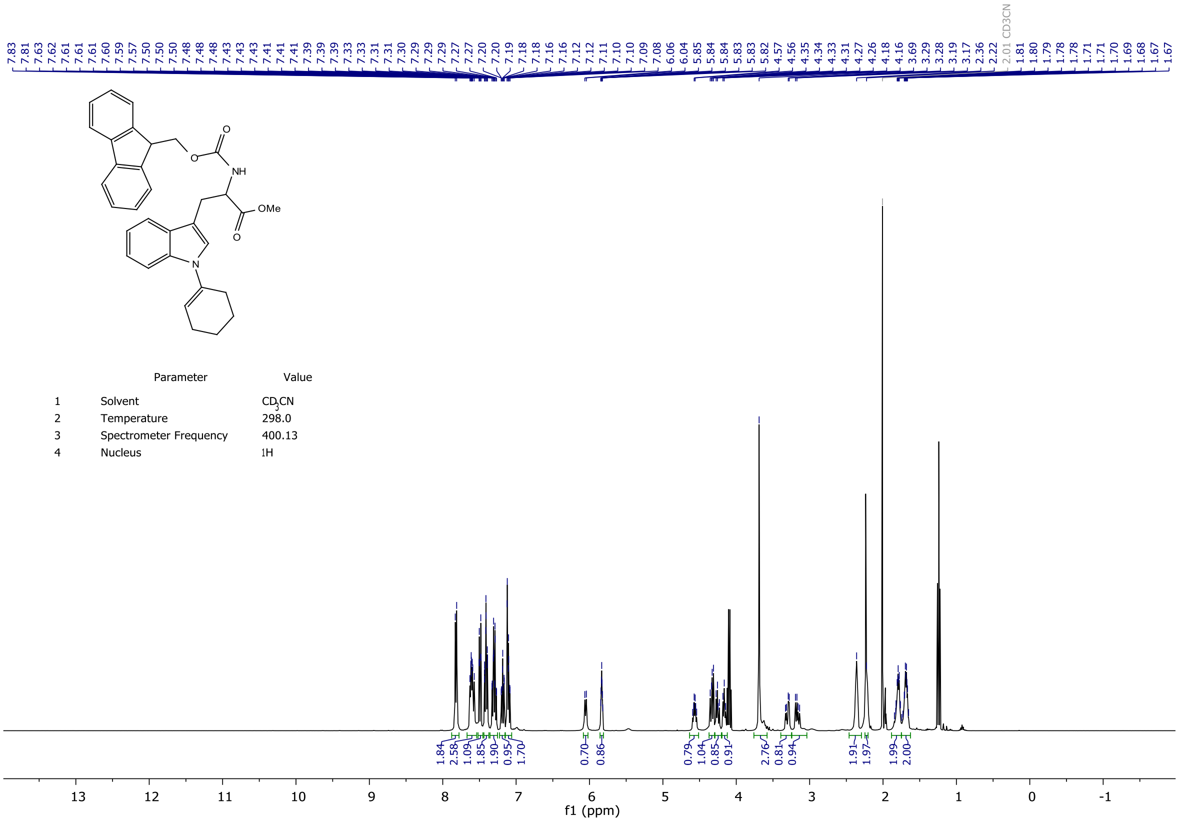

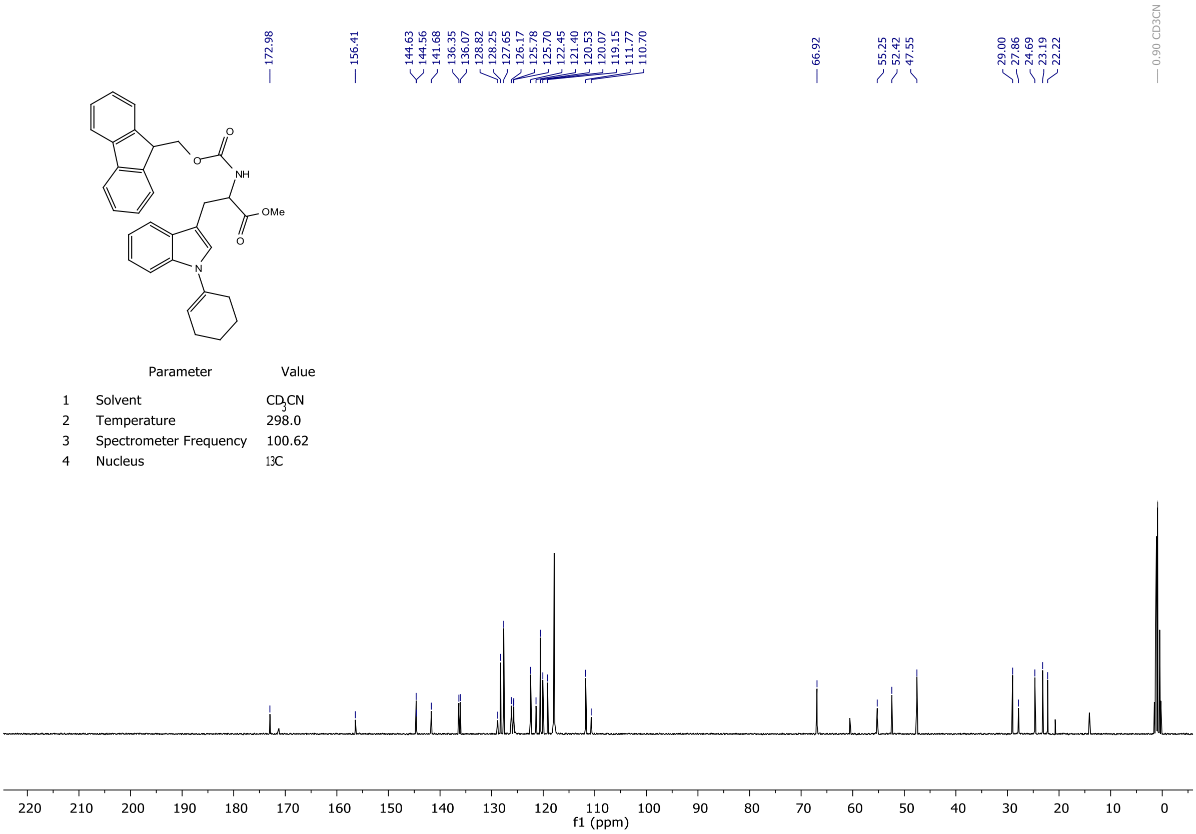

3-benzyl-1-(cyclohex-1-en-1-yl)-1H-indole, 3o

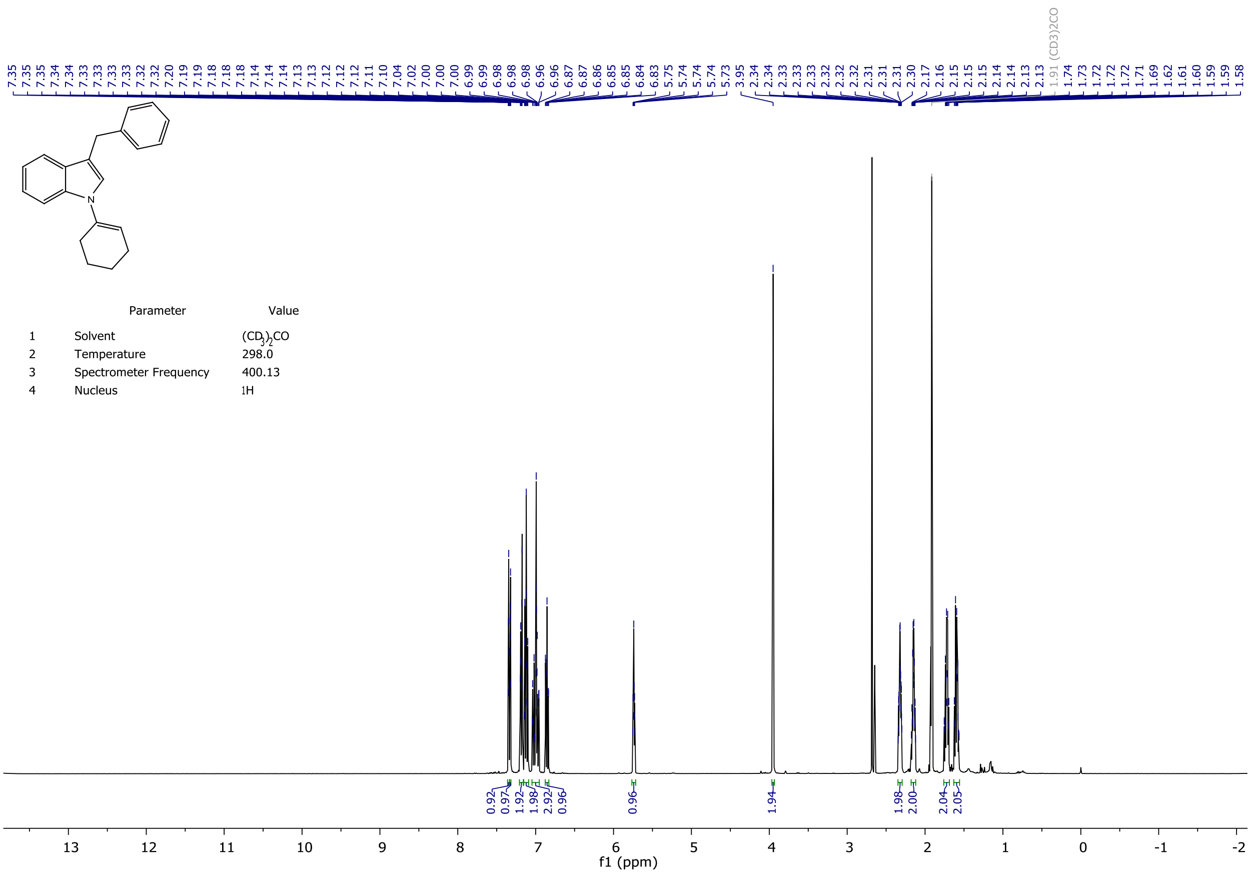

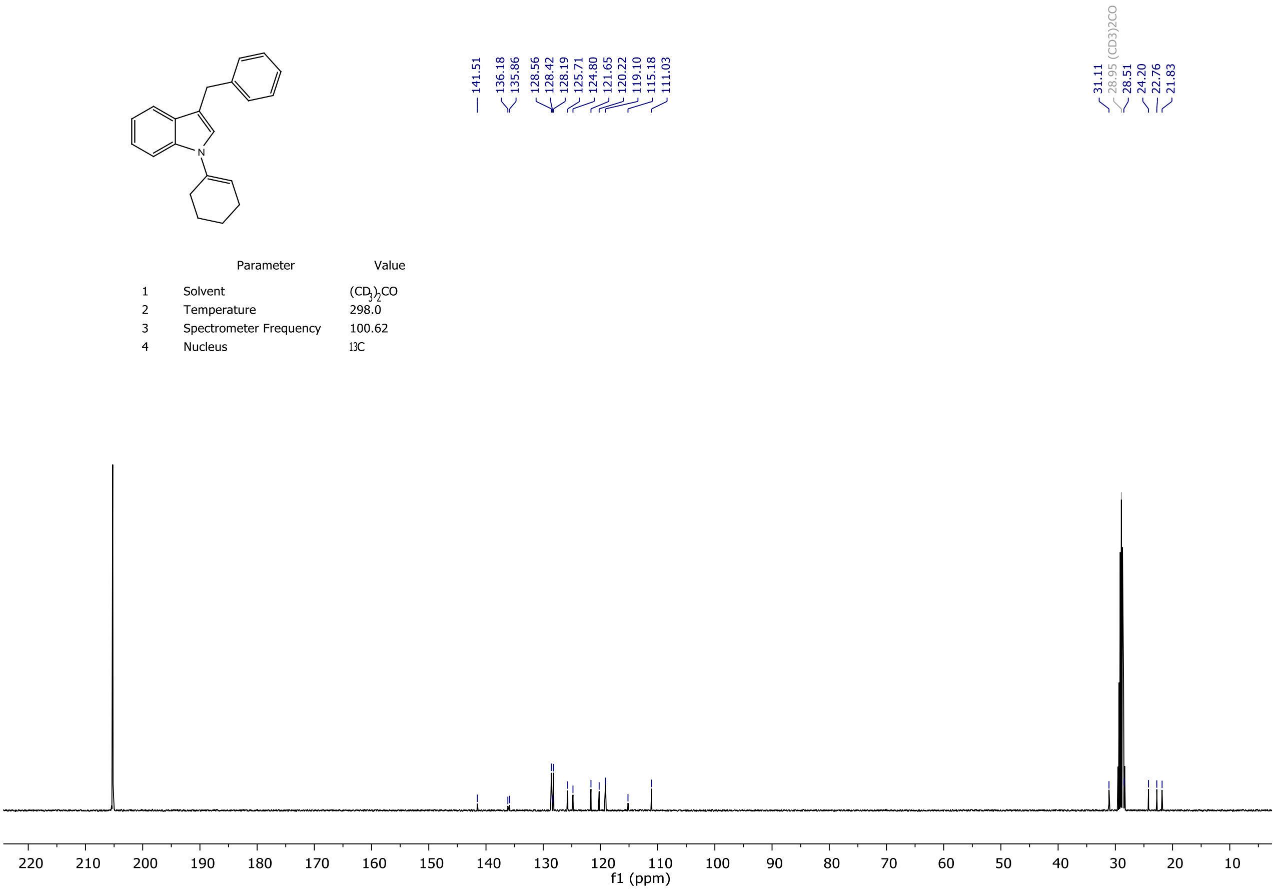

1-(cyclohex-1-en-1-yl)-3-phenyl-1H-indole, 3p

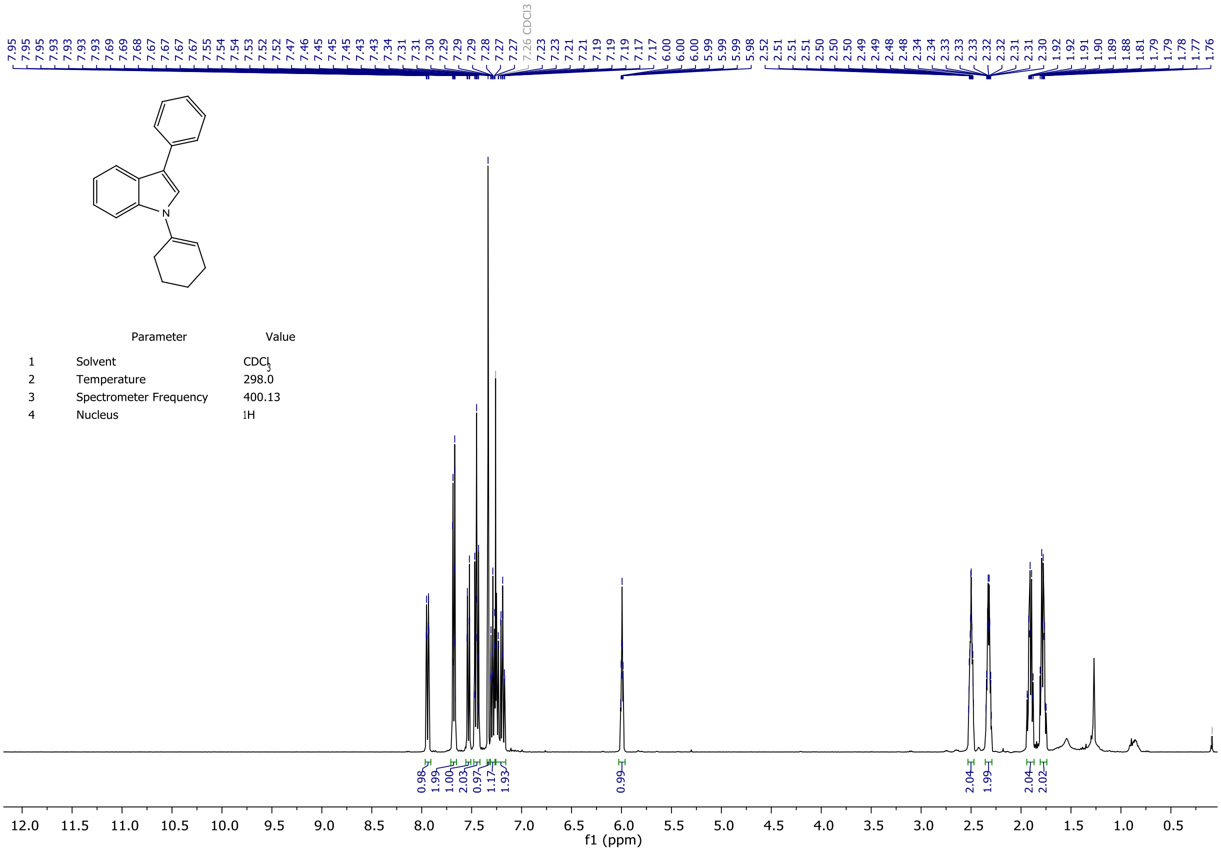

	Parameter	Value
1	Solvent	$CDCl_3$
2	Temperature	298.0
3	Spectrometer Frequency	400.13
4	Nucleus	1H

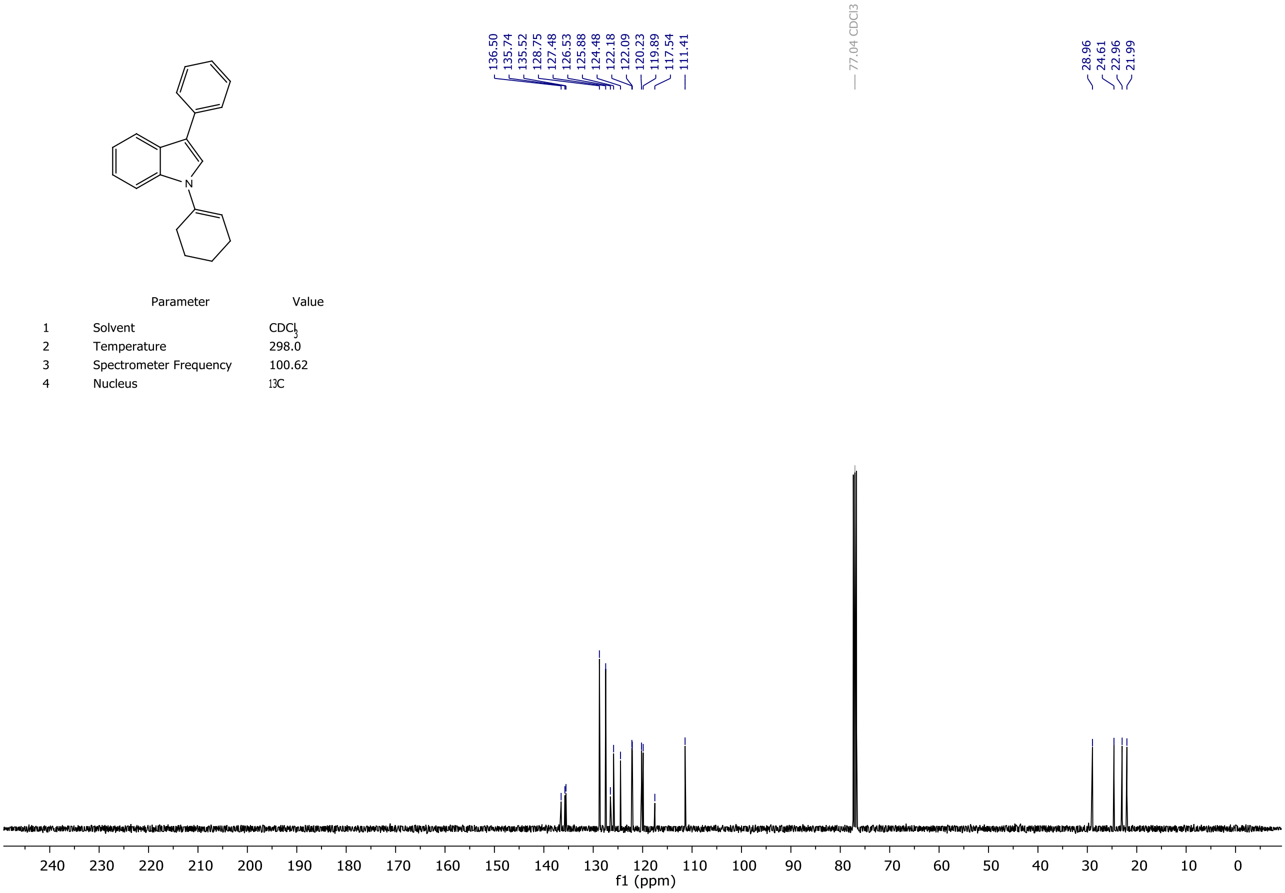

	Parameter	Value
1	Solvent	$CDCl_3$
2	Temperature	298.0
3	Spectrometer Frequency	100.62
4	Nucleus	13C

1,3-di(cyclohex-1-en-1-yl)-1H-indole, 3q

Bz9484_PP_2wash.1.fid

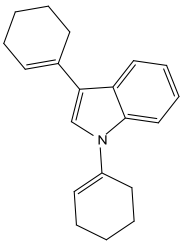

	Parameter	Value
1	Solvent	$(CD_3)_2CO$
2	Temperature	298.0
3	Spectrometer Frequency	100.62
4	Nucleus	13C

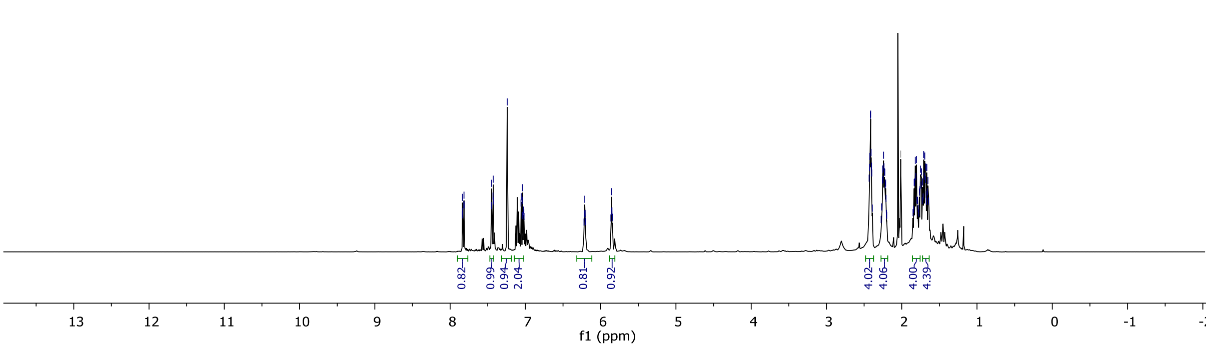

Bz9484_PP_2wash.2.fid

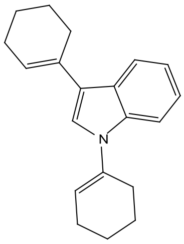

	Parameter	Value
1	Solvent	$CDCl_3$
2	Temperature	298.0
3	Spectrometer Frequency	100.62
4	Nucleus	13C

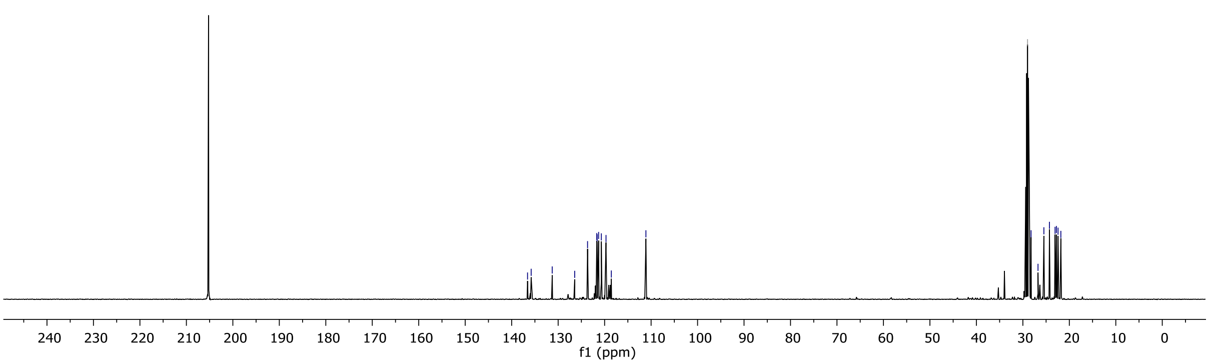

1-(cyclohept-1-en-1-yl)-3-methyl-1H-indole, 5a CAS 2122299-51-6 [4]

BW4681-01-HNMR-Acetone.1.fid

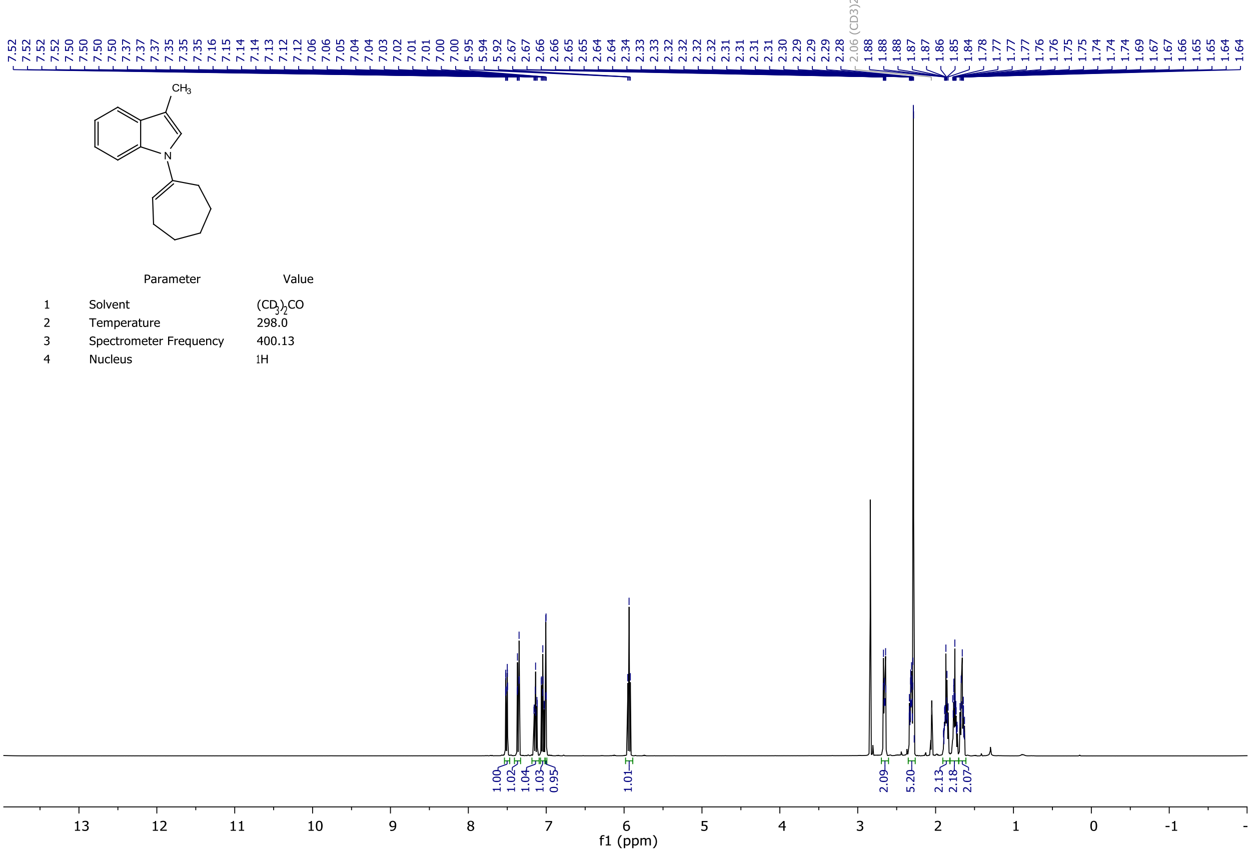

BW4681-01-CNMR-Acetone.1.fid

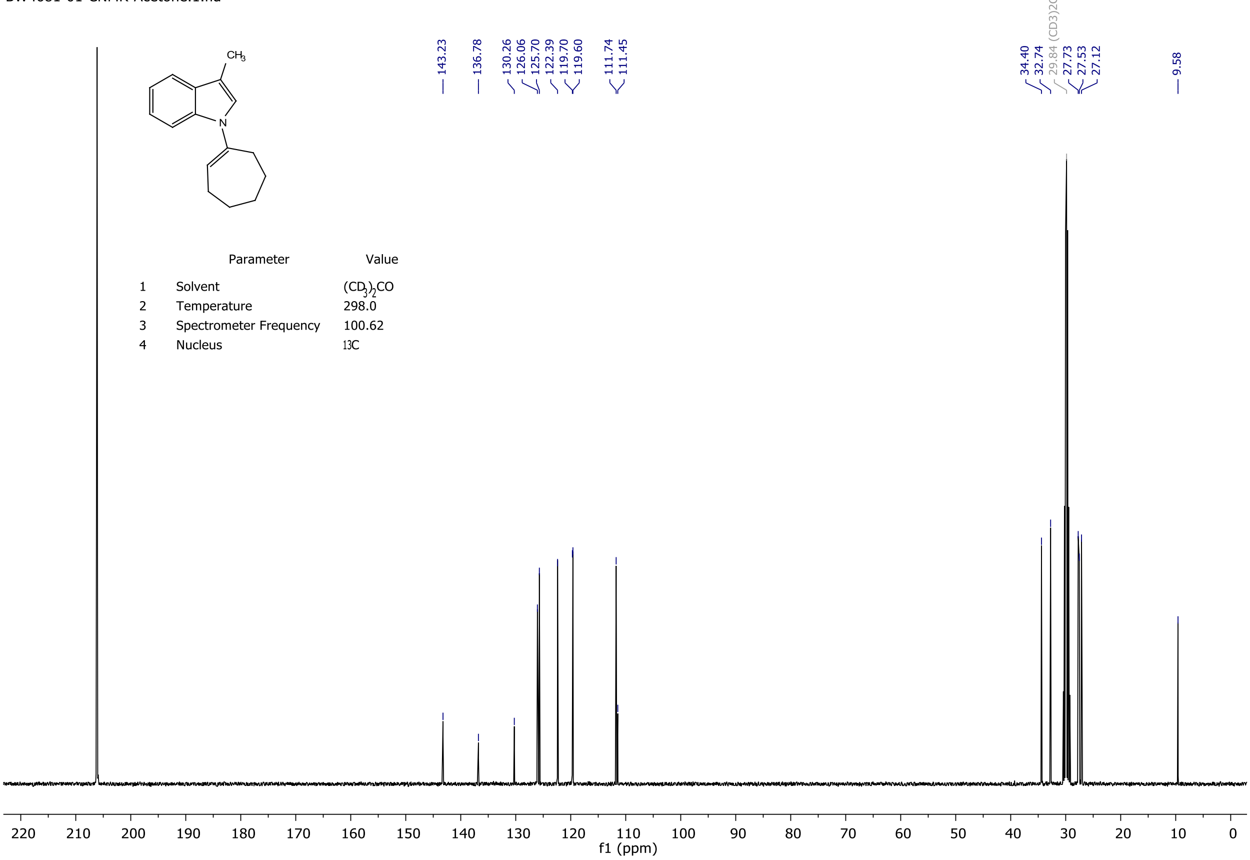

1-(but-2-en-2-yl)-3-methyl-1H-indole, 5b

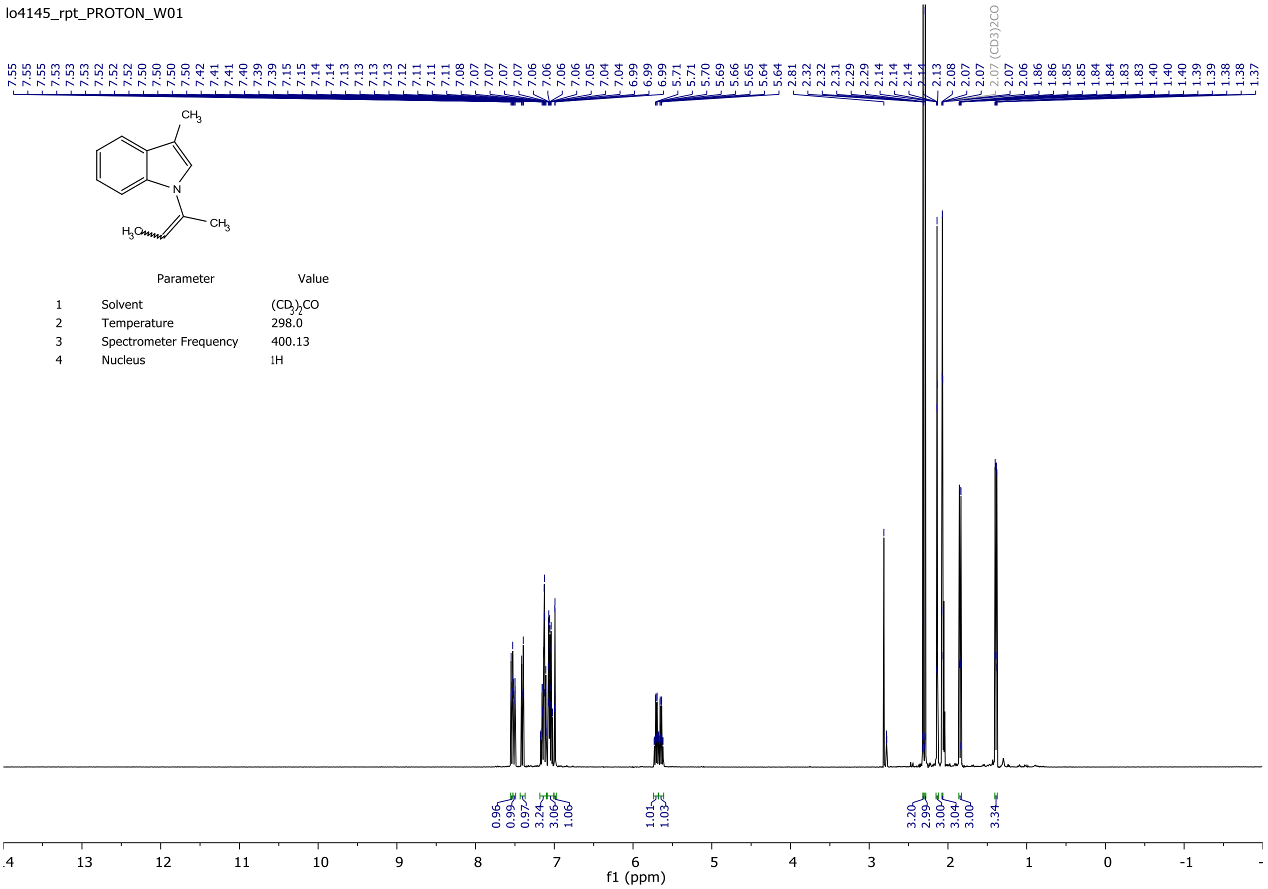

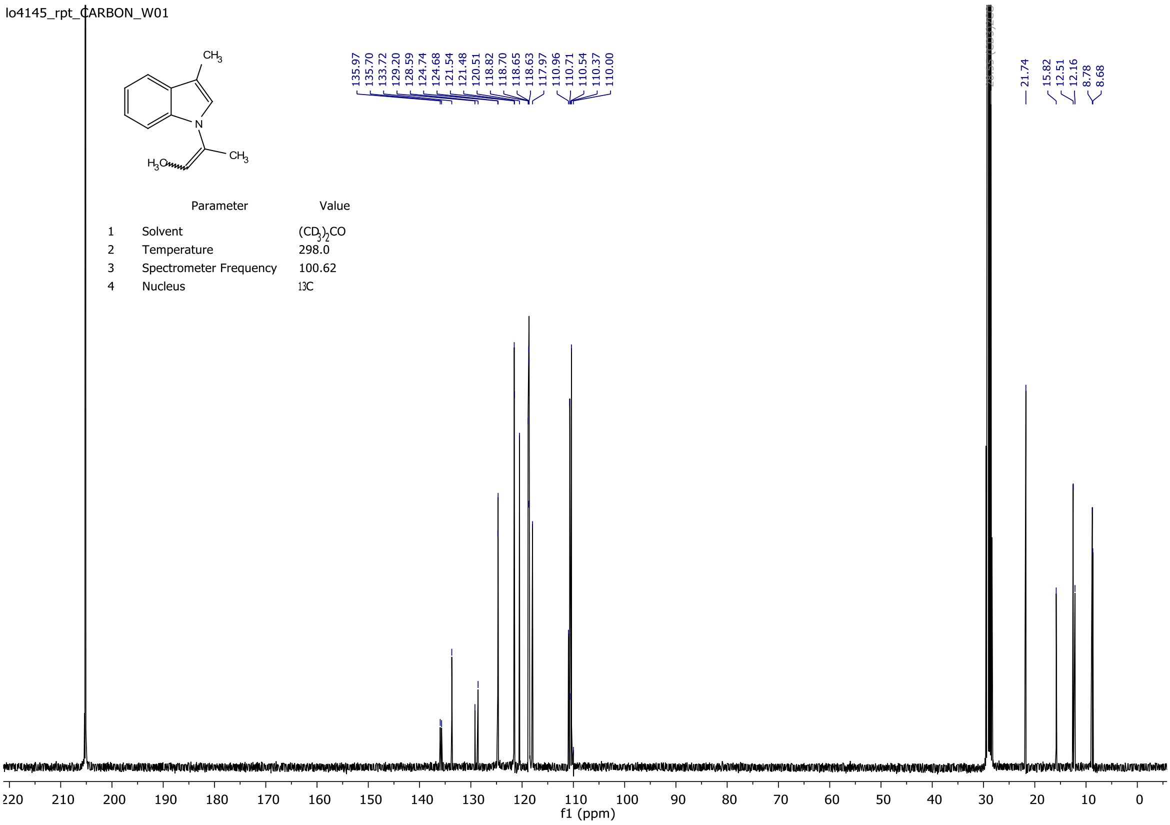

3-methyl-1-(pent-2-en-3-yl)-1H-indole, 5c

lo5143_chr_PROTON_01

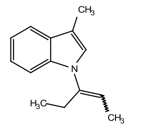

	Parameter	Value
1	Solvent	$(CD_3)_2CO$
2	Temperature	298.0
3	Spectrometer Frequency	400.13
4	Nucleus	1H

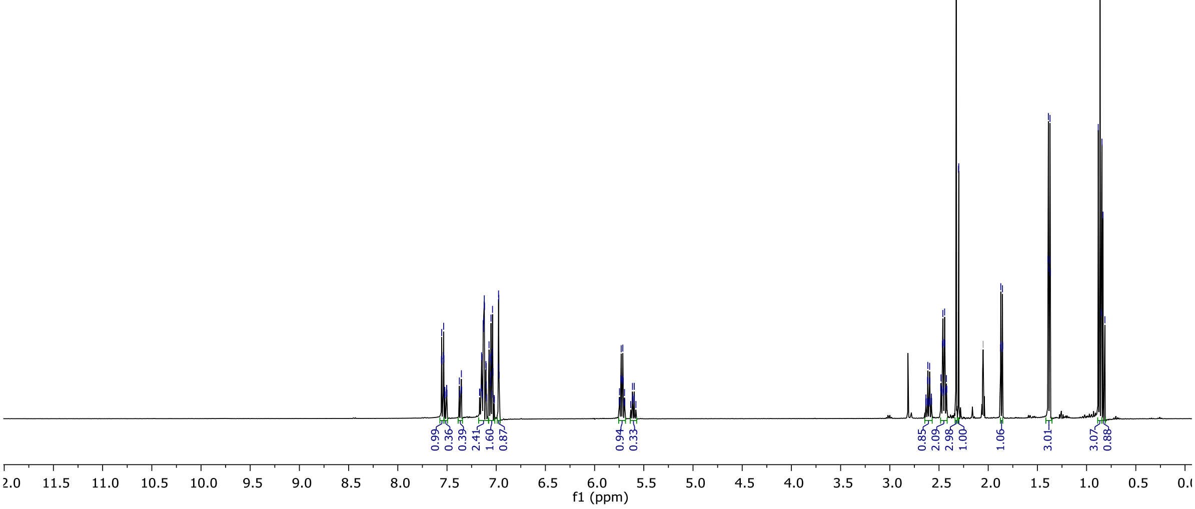

lo5143_chr_CARBON_01

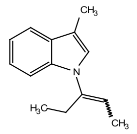

	Parameter	Value
1	Solvent	$(CD_3)_2CO$
2	Temperature	298.0
3	Spectrometer Frequency	100.62
4	Nucleus	13C

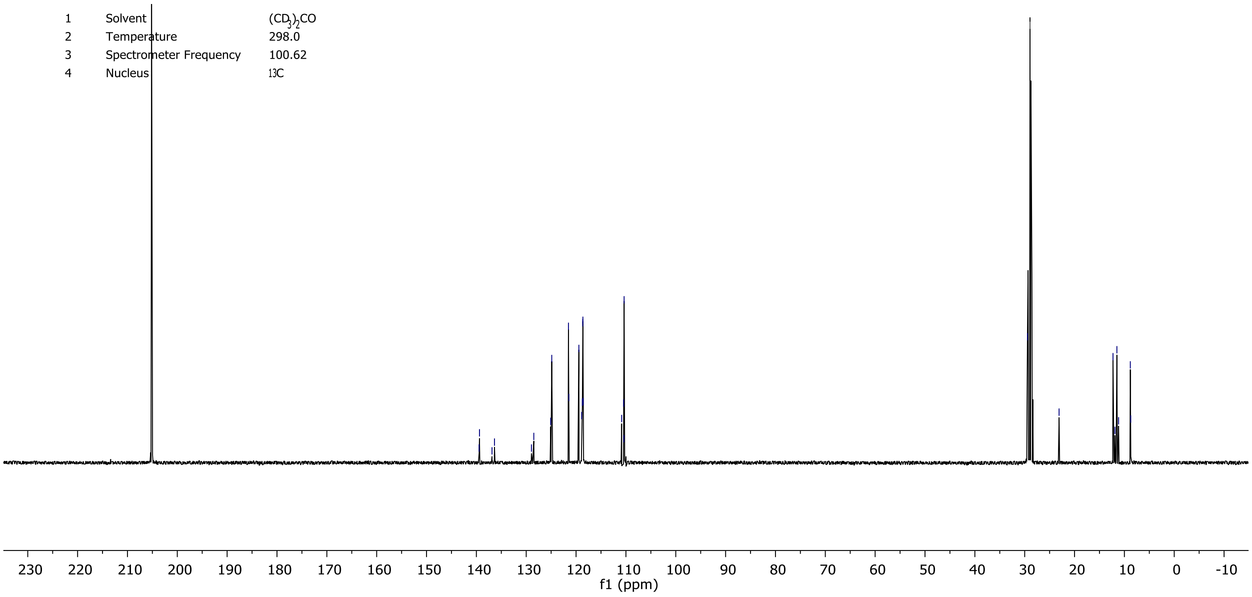

LOBB5443.18.fid
1D Selective Gradient NOESY
freq: 6.995ppm

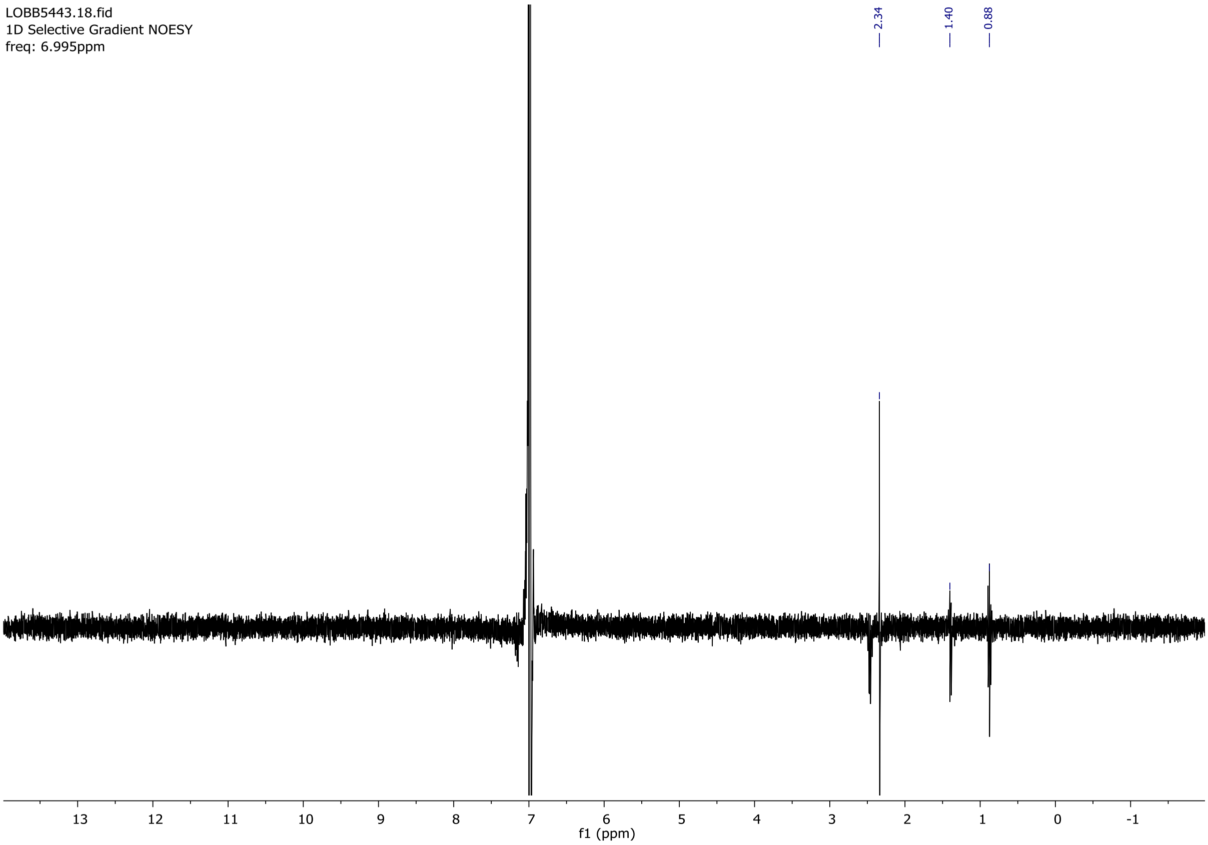

1-[(Z)-1-isobutyl-3-methyl-but-1-enyl]-3-methyl-indole and 1-[(E)-1-isobutyl-3-methyl-but-1-enyl]-3-methyl-indole, 5d

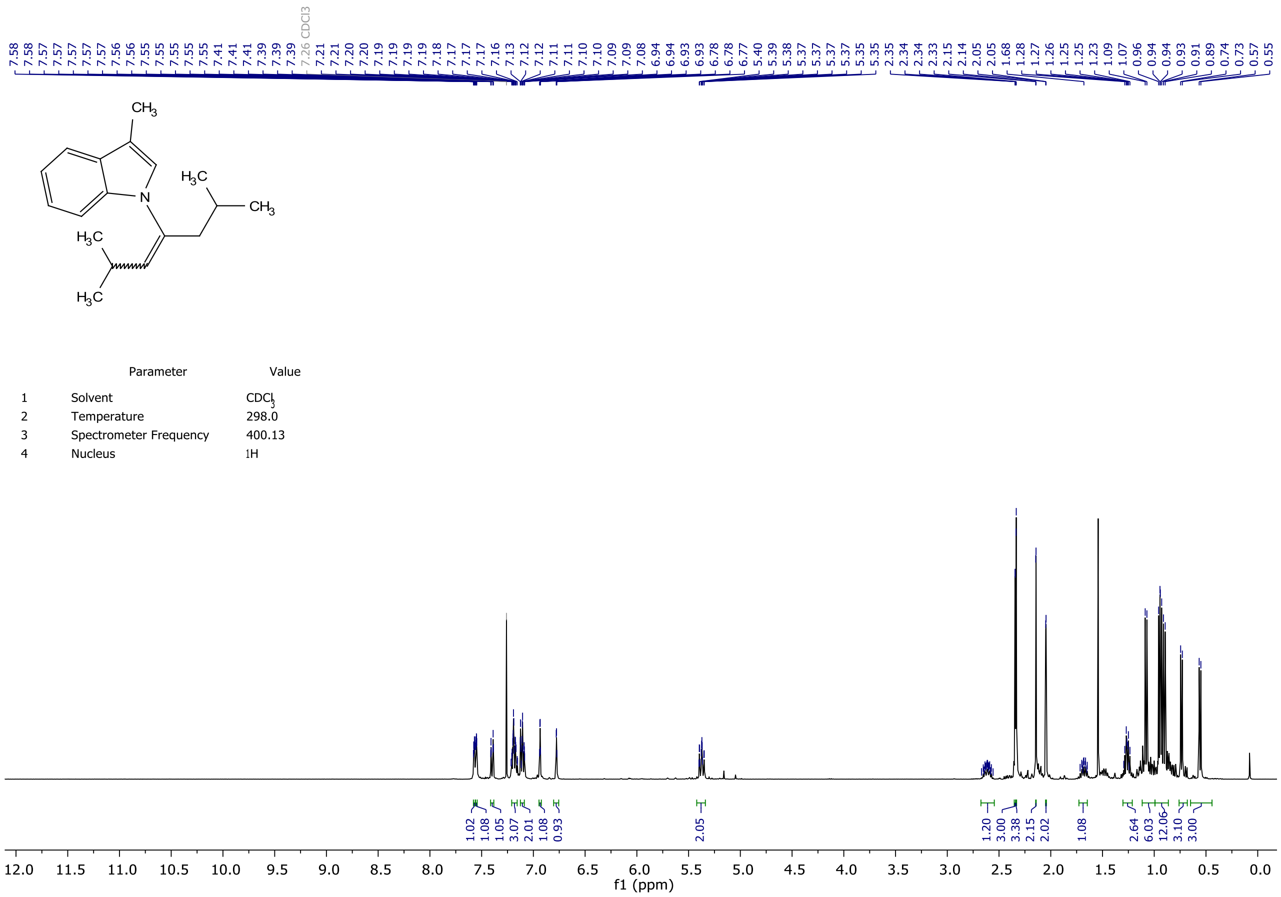

	Parameter	Value
1	Solvent	$CDCl_3$
2	Temperature	298.0
3	Spectrometer Frequency	400.13
4	Nucleus	1H

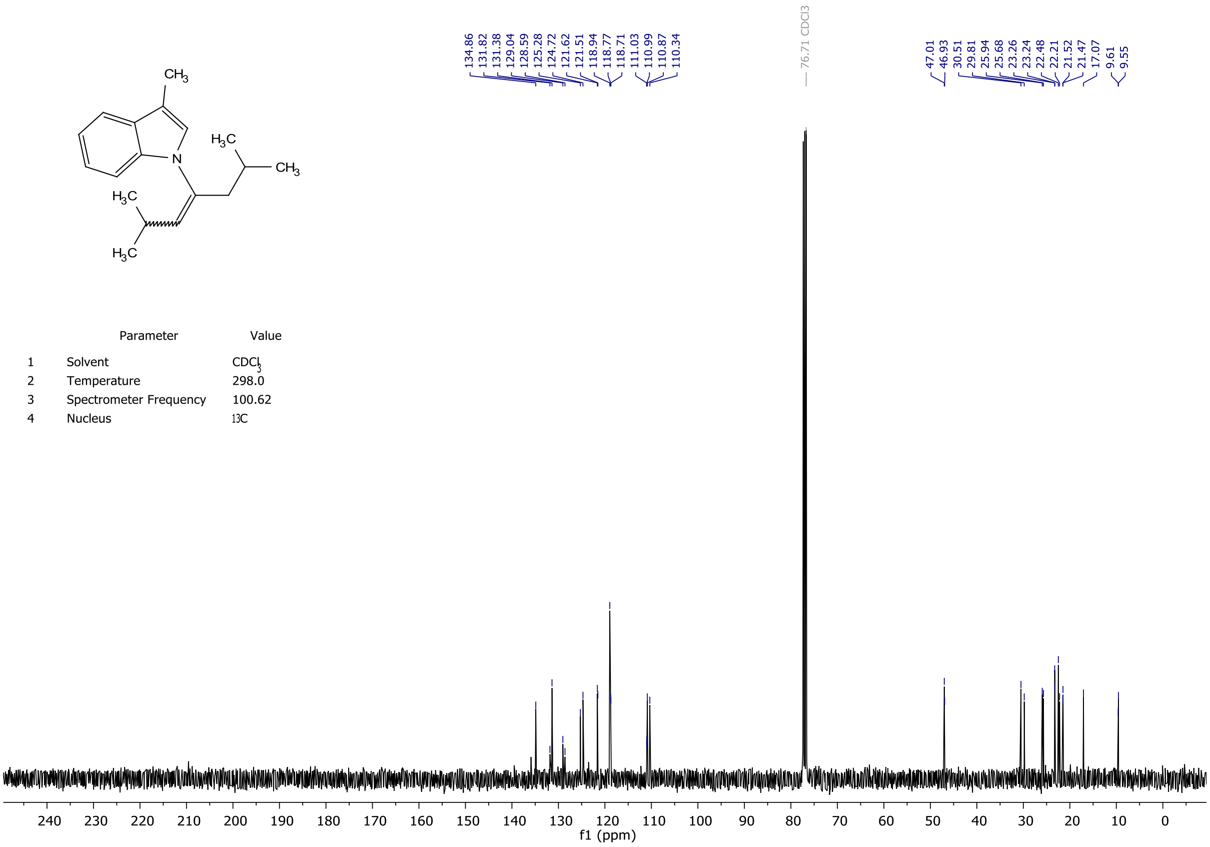

	Parameter	Value
1	Solvent	$CDCl_3$
2	Temperature	298.0
3	Spectrometer Frequency	100.62
4	Nucleus	13C

3-methyl-1-(1-phenylvinyl)-1H-indole, 5e CAS1176684-23-3[5]

lo5144_chr_PROTON_01

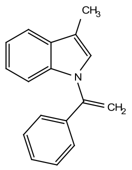

	Parameter	Value
1	Solvent	$(CD_3)_2CO$
2	Temperature	298.0
3	Spectrometer Frequency	400.13
4	Nucleus	1H

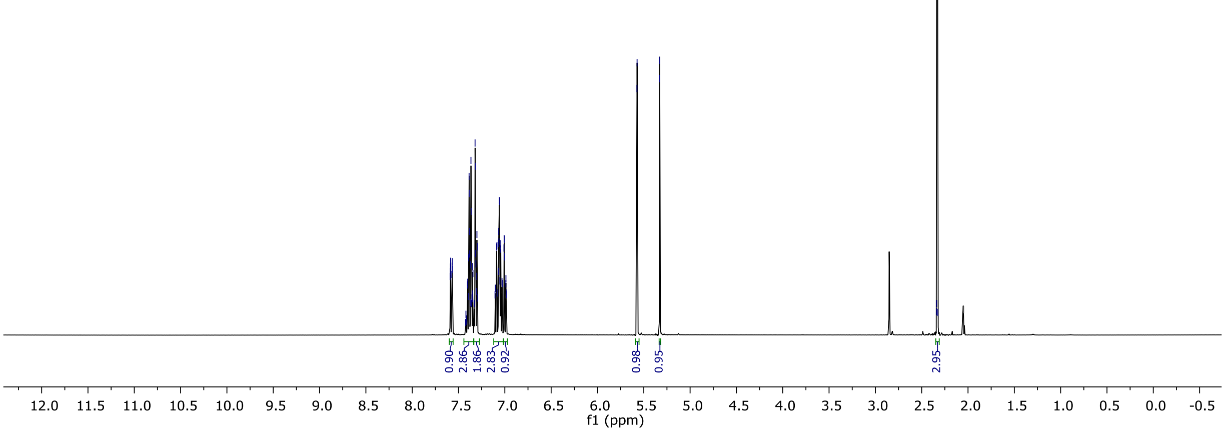

lo5144_chr_CARBON_01

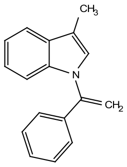

	Parameter	Value
1	Solvent	$(CD_3)_2CO$
2	Temperature	298.0
3	Spectrometer Frequency	100.62
4	Nucleus	13C

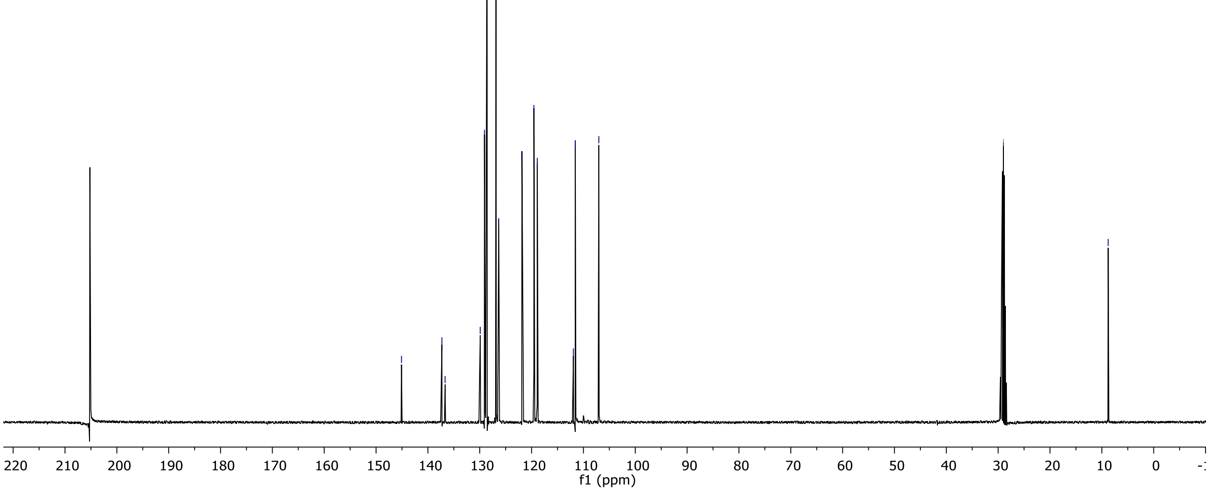

1-(4-tert-butylcyclohexen-1-yl)-3-methyl-indole, 5f

BW4694-01-HNMR-Acetone.1.fid

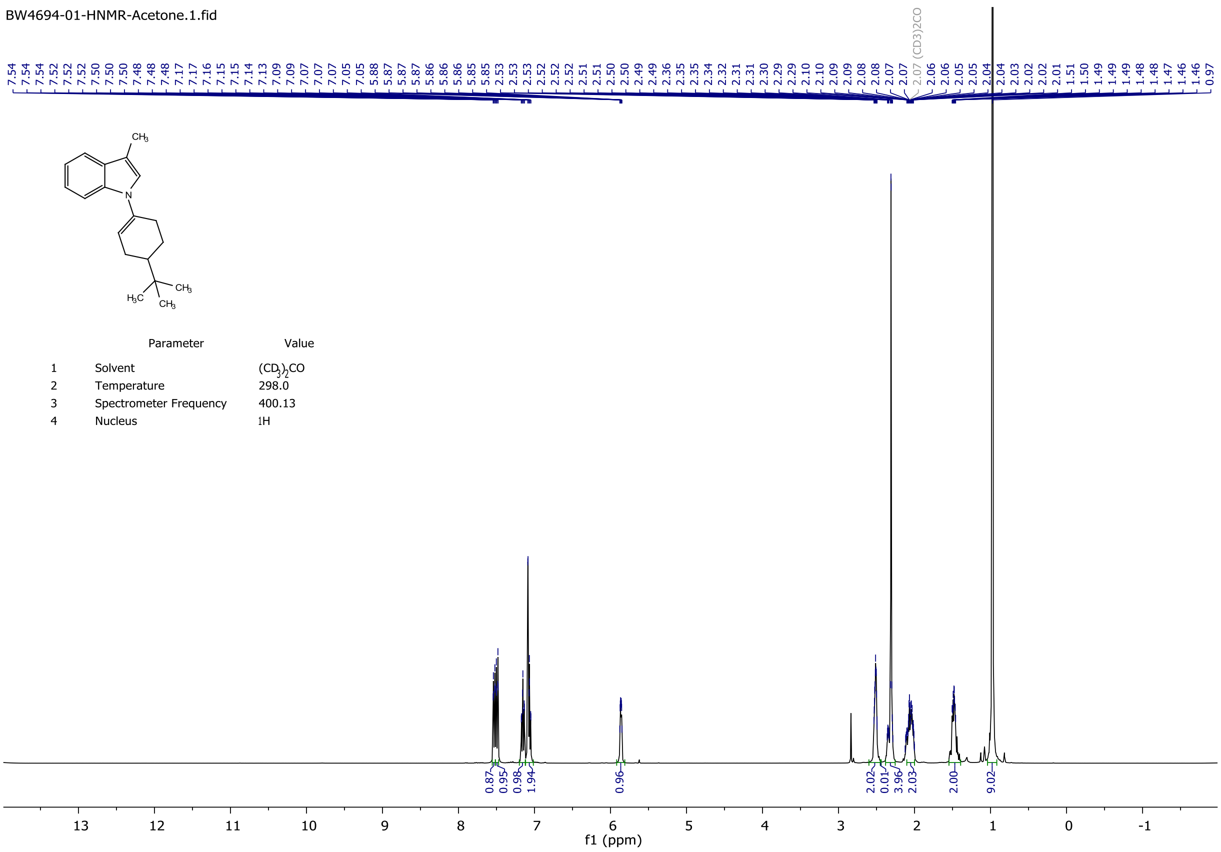

	Parameter	Value
1	Solvent	$(CD_3)_2CO$
2	Temperature	298.0
3	Spectrometer Frequency	400.13
4	Nucleus	1H

BW4694-01-CNMR-Acetone.1.fid

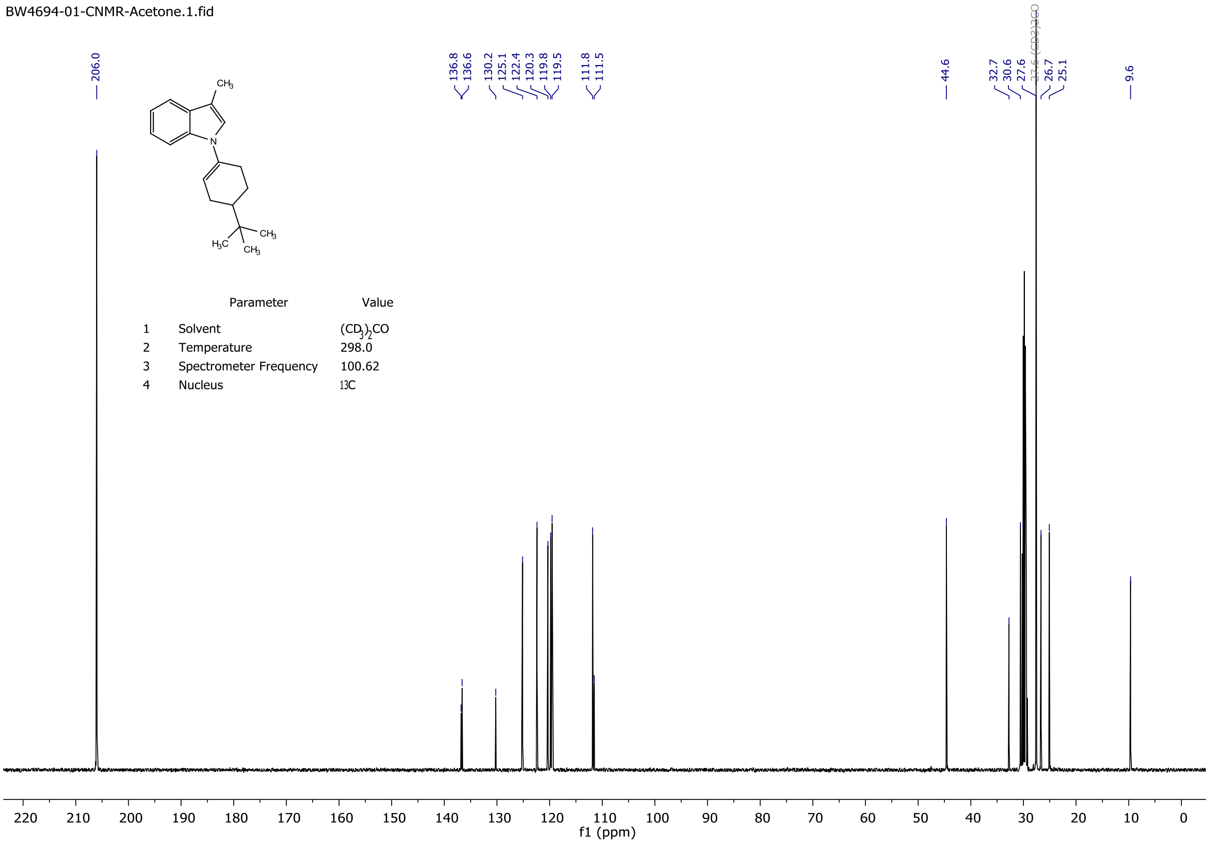

	Parameter	Value
1	Solvent	$(CD_3)_2CO$
2	Temperature	298.0
3	Spectrometer Frequency	100.62
4	Nucleus	13C

1-(3,4-dihydronaphthalen-2-yl)-3-methyl-indole, 5g

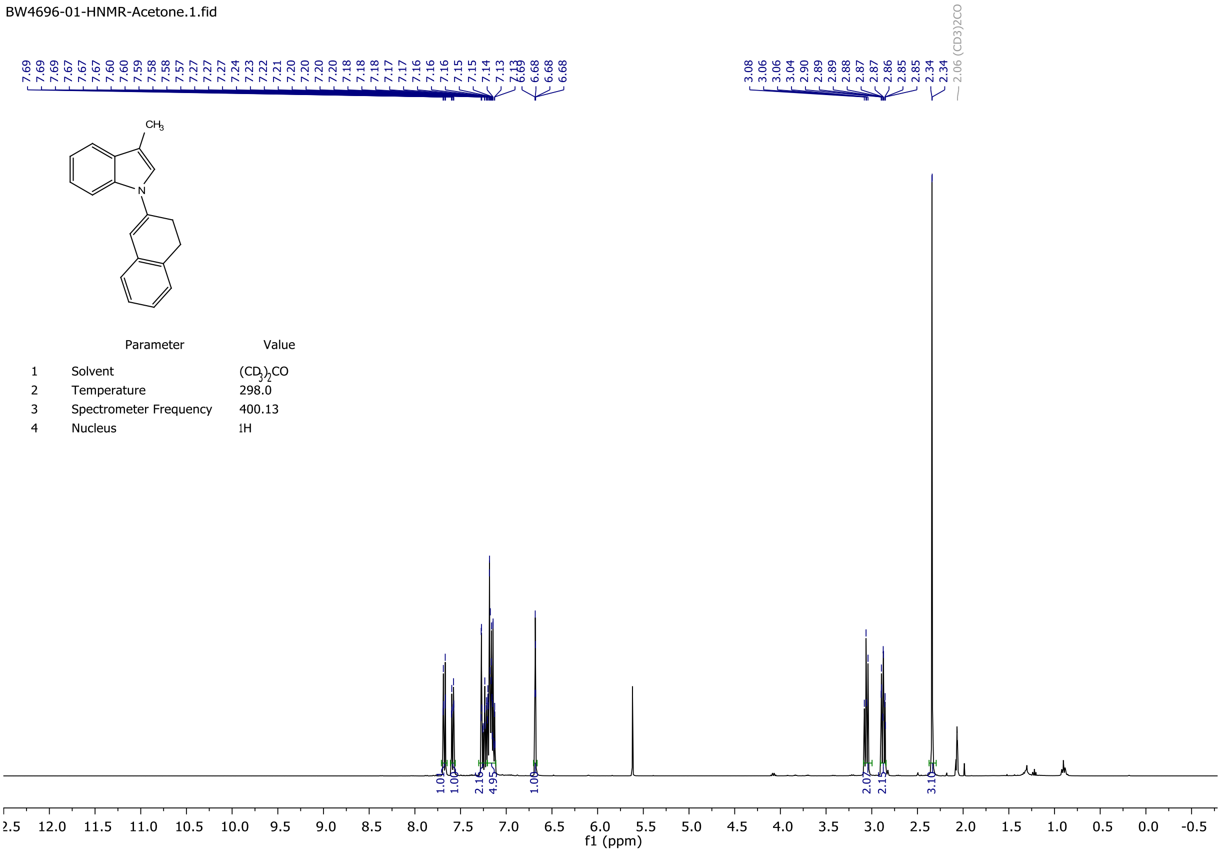

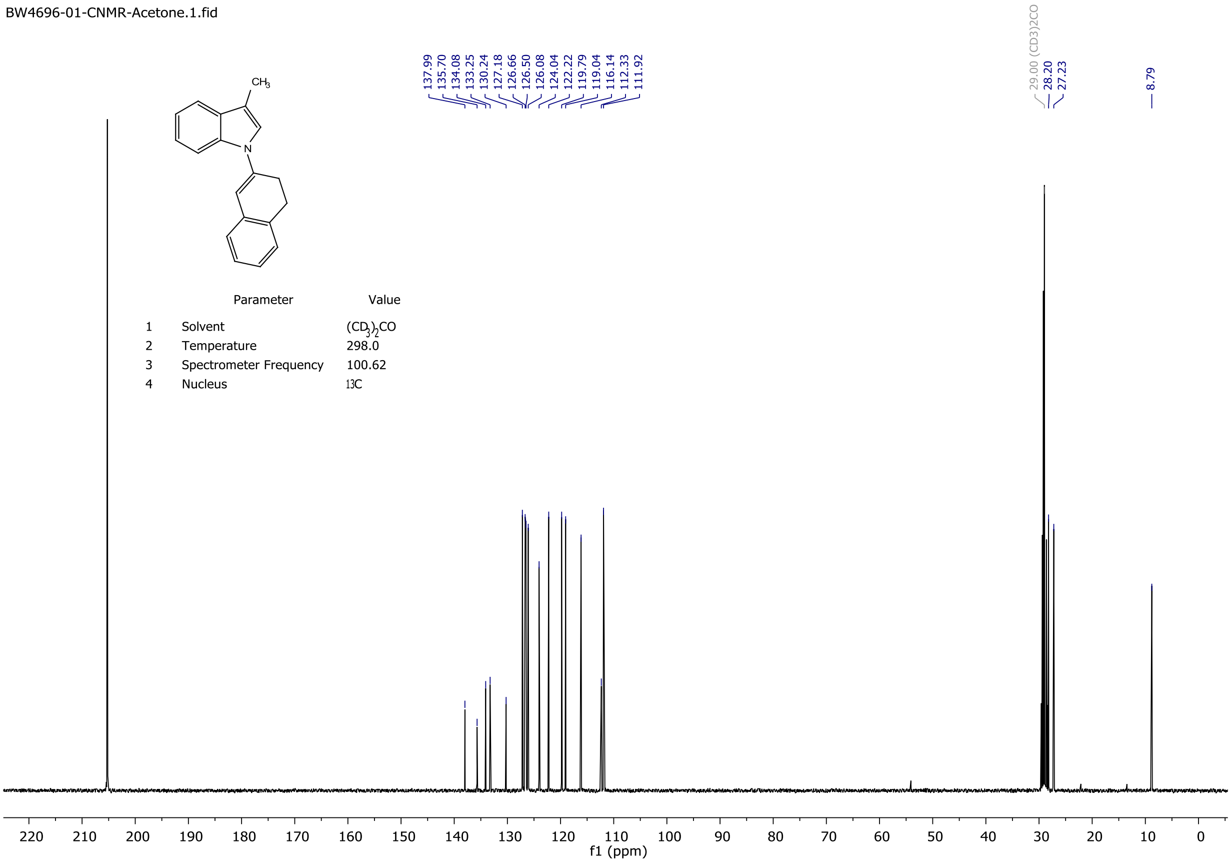

1-(3,6-dihydro-2H-pyran-4-yl)-3-methyl-1H-indole), 5h

BX3328 Second Column.1.fid

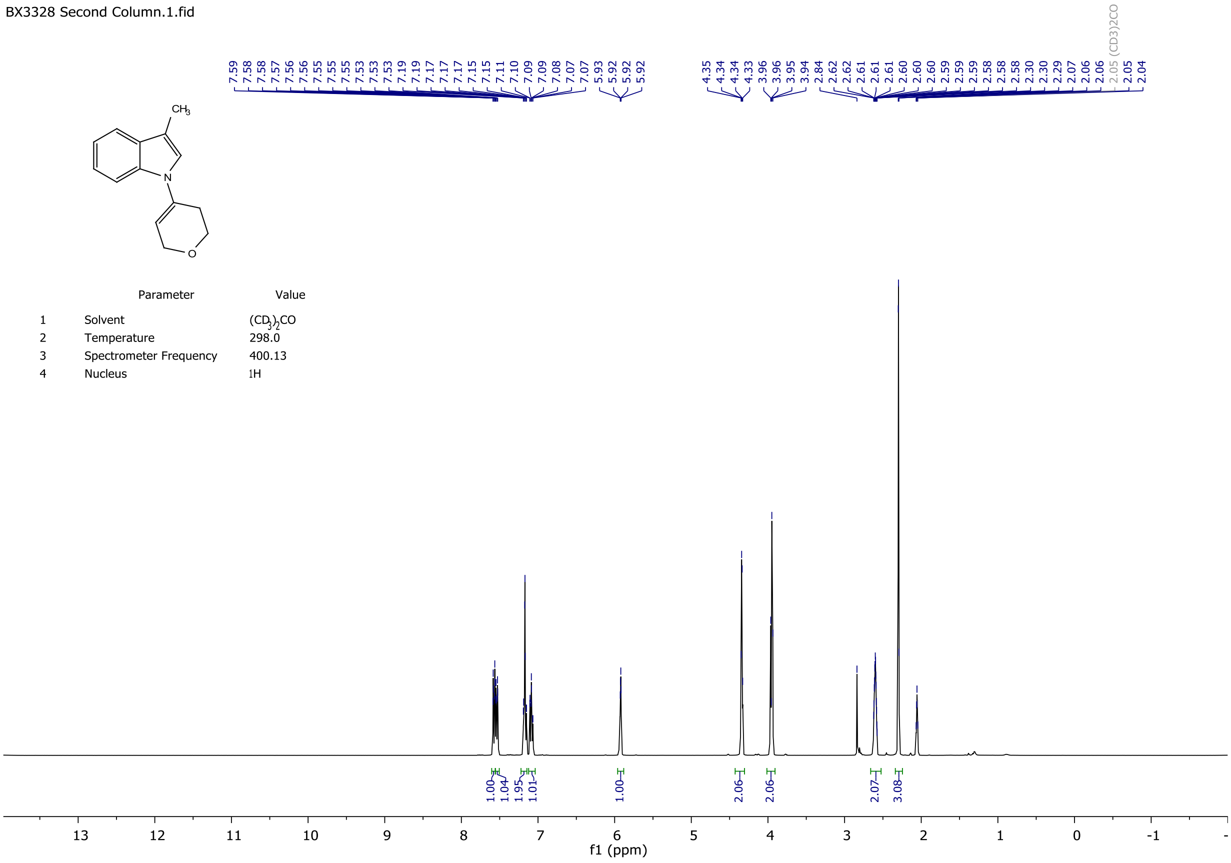

	Parameter	Value
1	Solvent	$(CD_3)_2CO$
2	Temperature	298.0
3	Spectrometer Frequency	400.13
4	Nucleus	1H

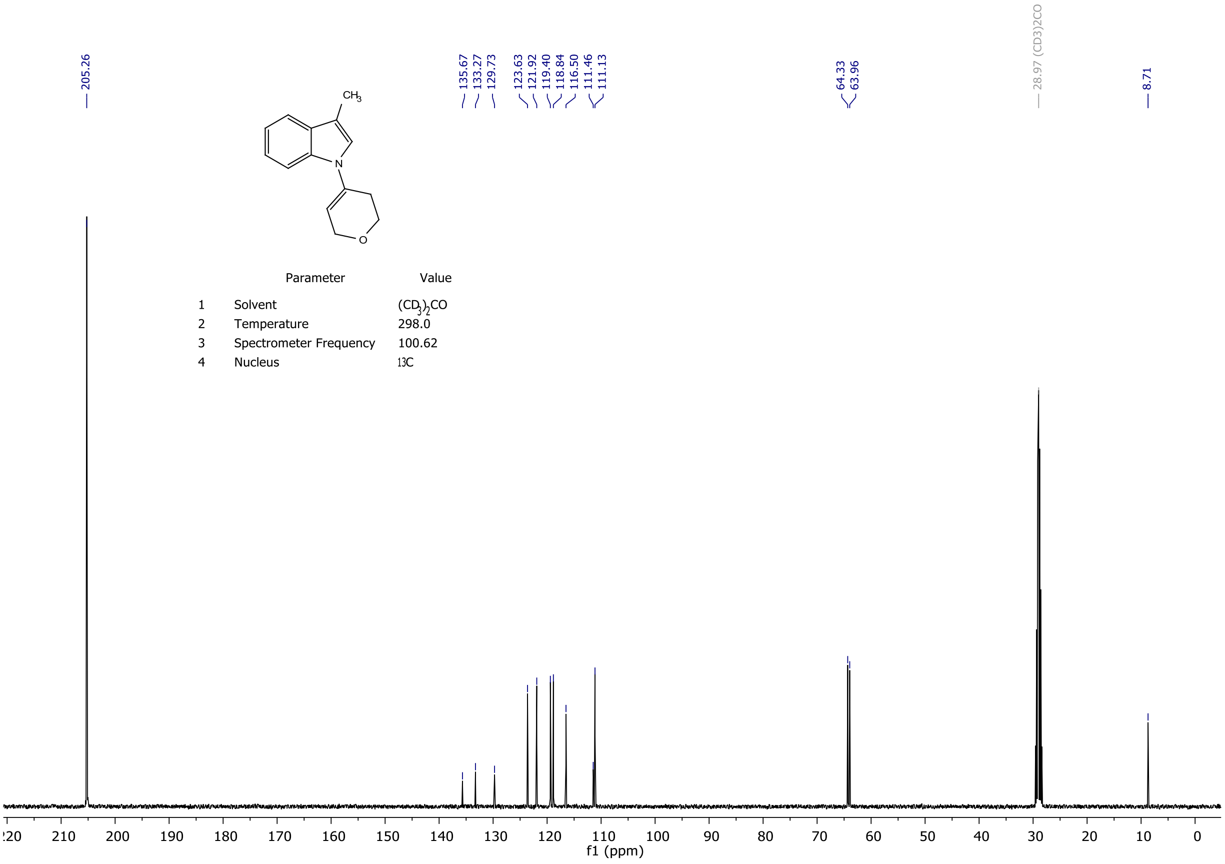

	Parameter	Value
1	Solvent	$(CD_3)_2CO$
2	Temperature	298.0
3	Spectrometer Frequency	100.62
4	Nucleus	13C

Ethyl 4-(3-methyl-1H-indol-1-yl)cyclohex-3-ene-1-carboxylate, 5i

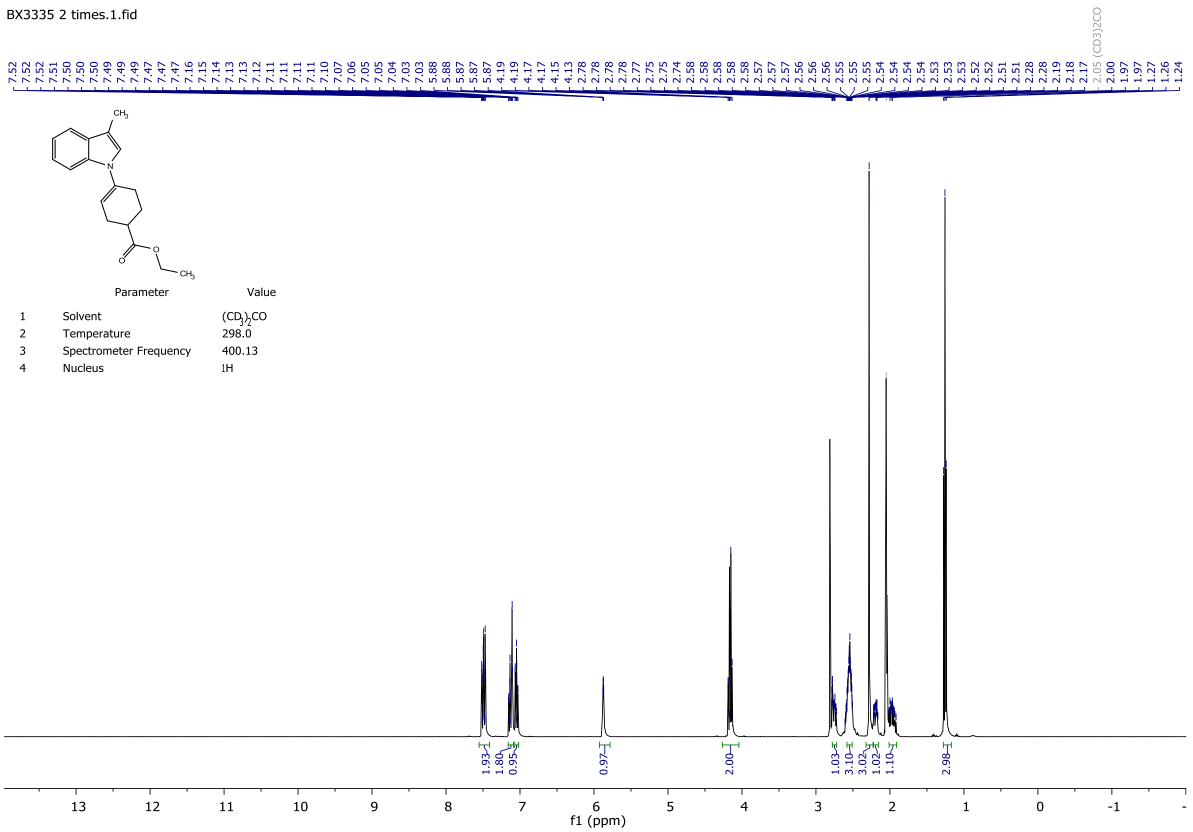

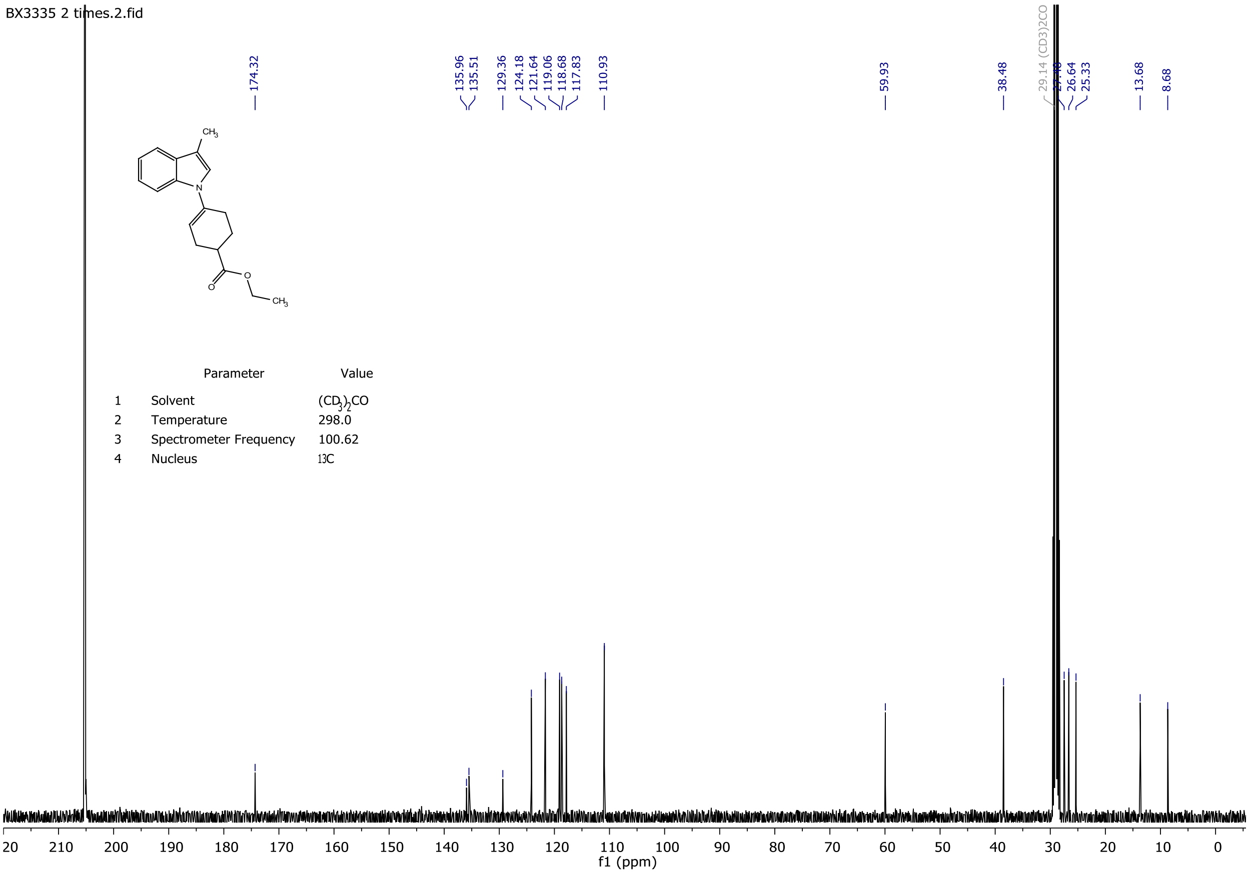

Tert-butyl 4-(3-methylindol-1-yl)-3,6-dihydro-2H-pyridine-1-carboxylate, 5j

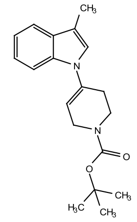

	Parameter	Value
1	Solvent	$(CD_3)_2CO$
2	Temperature	298.0
3	Spectrometer Frequency	400.13
4	Nucleus	1H

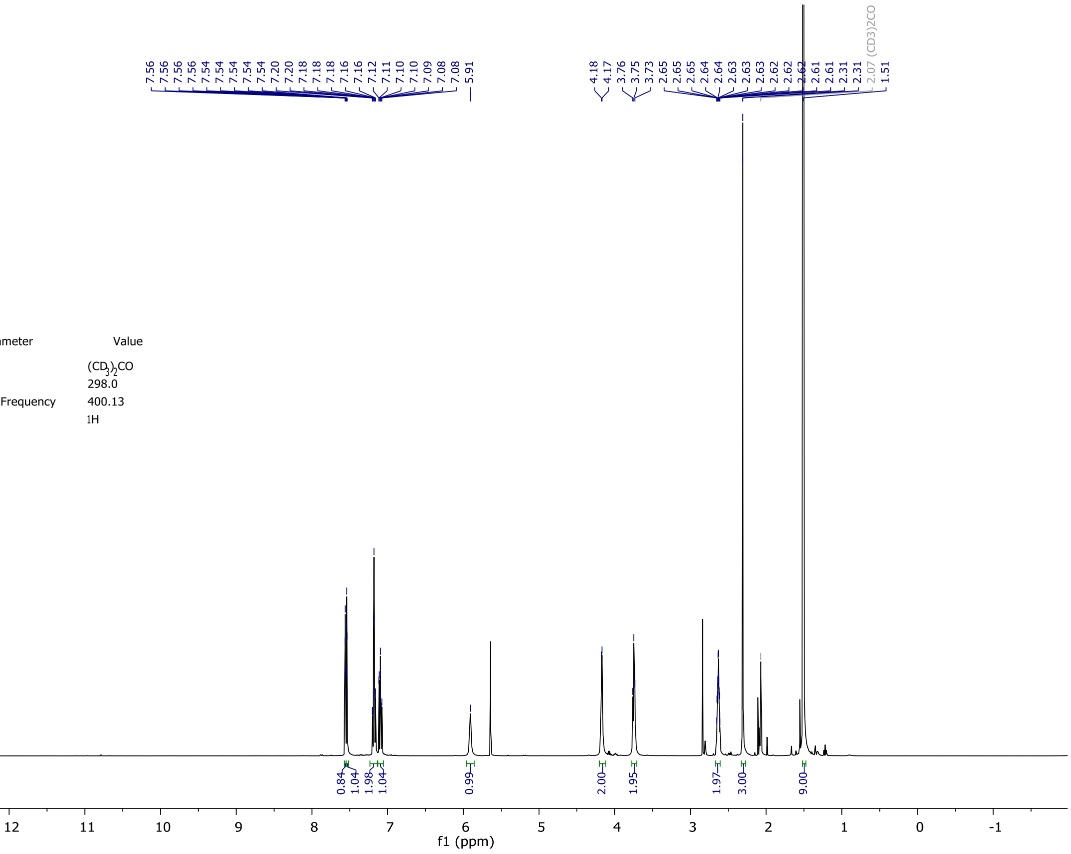

BX3330 rt 2 Column.2.fid

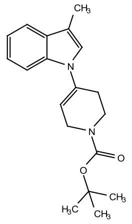

	Parameter	Value
1	Solvent	$(CD_3)_2CO$
2	Temperature	298.0
3	Spectrometer Frequency	100.62
4	Nucleus	13C

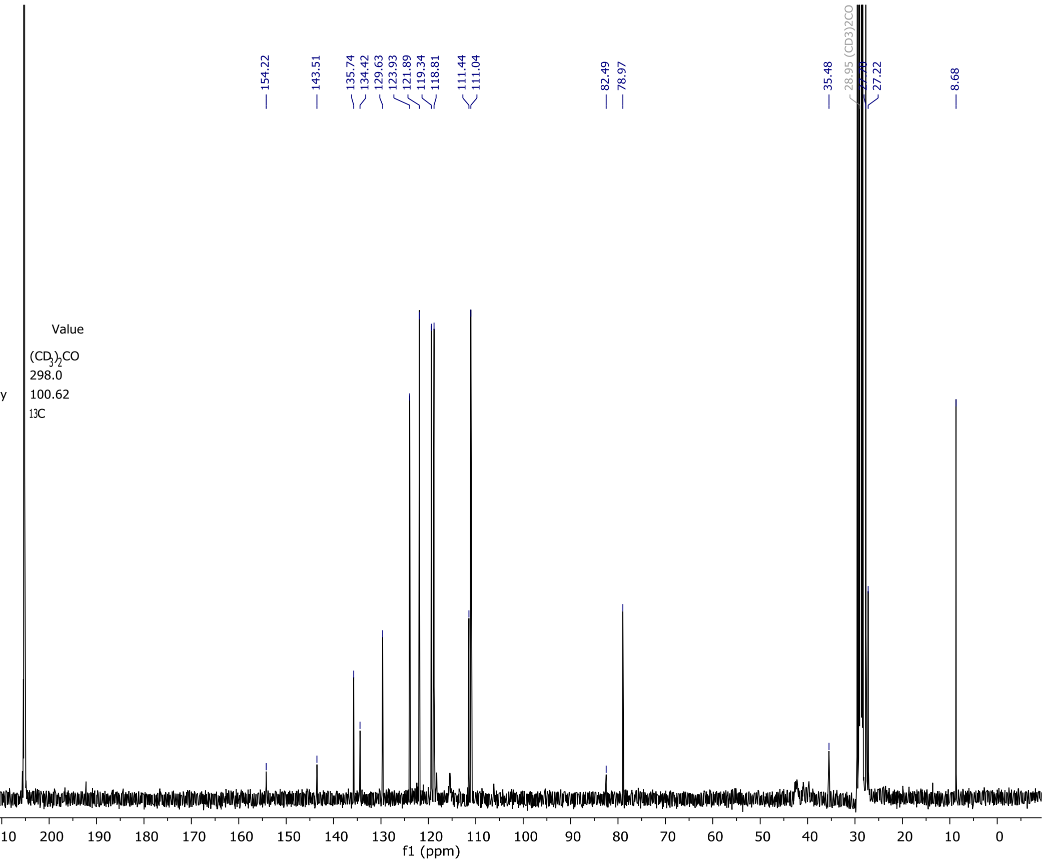

ethyl (E)-3-(3-methyl-1H-indol-1-yl)but-2-enoate, 5m

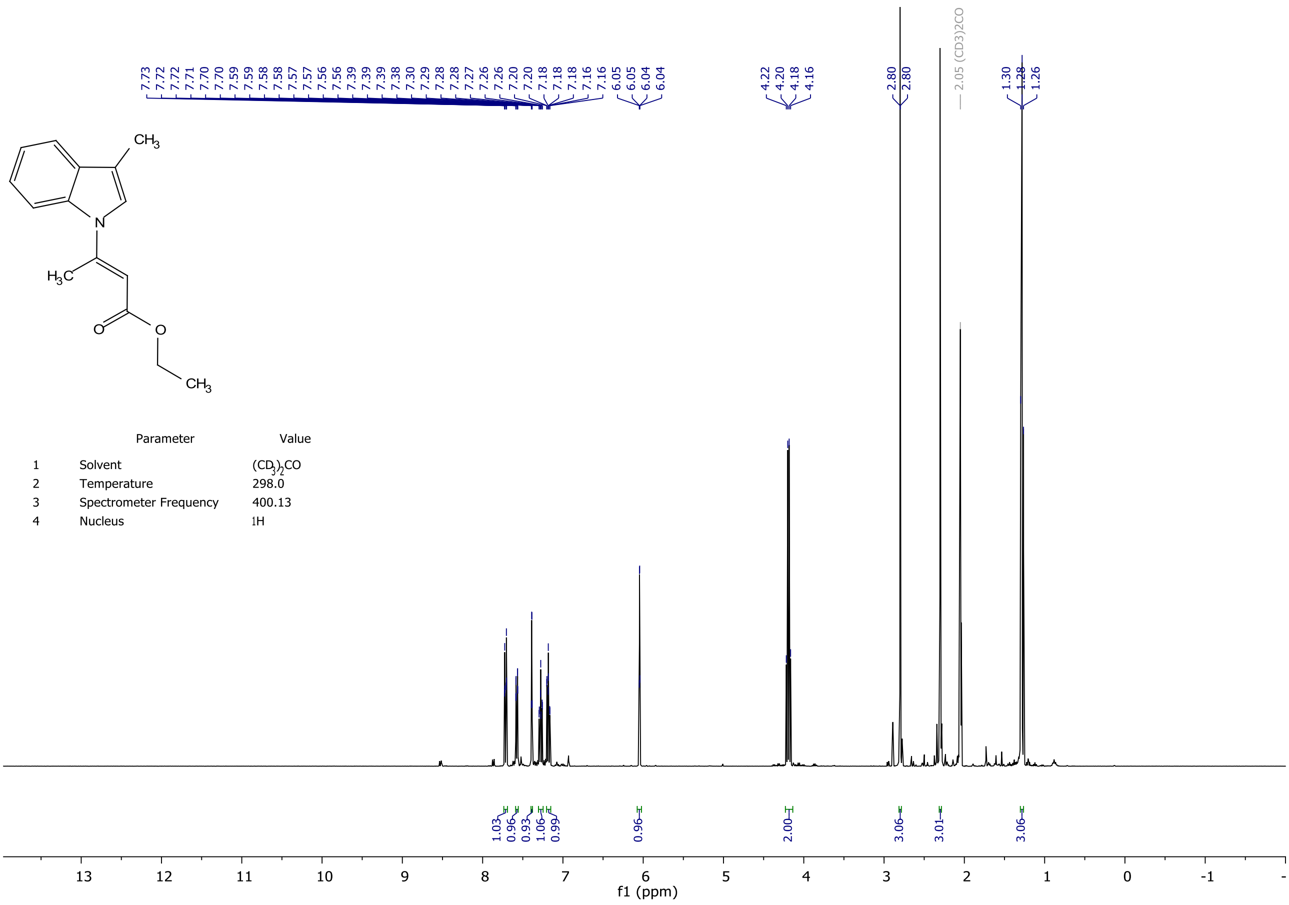

	Parameter	Value
1	Solvent	$(CD_3)_2CO$
2	Temperature	298.0
3	Spectrometer Frequency	400.13
4	Nucleus	1H

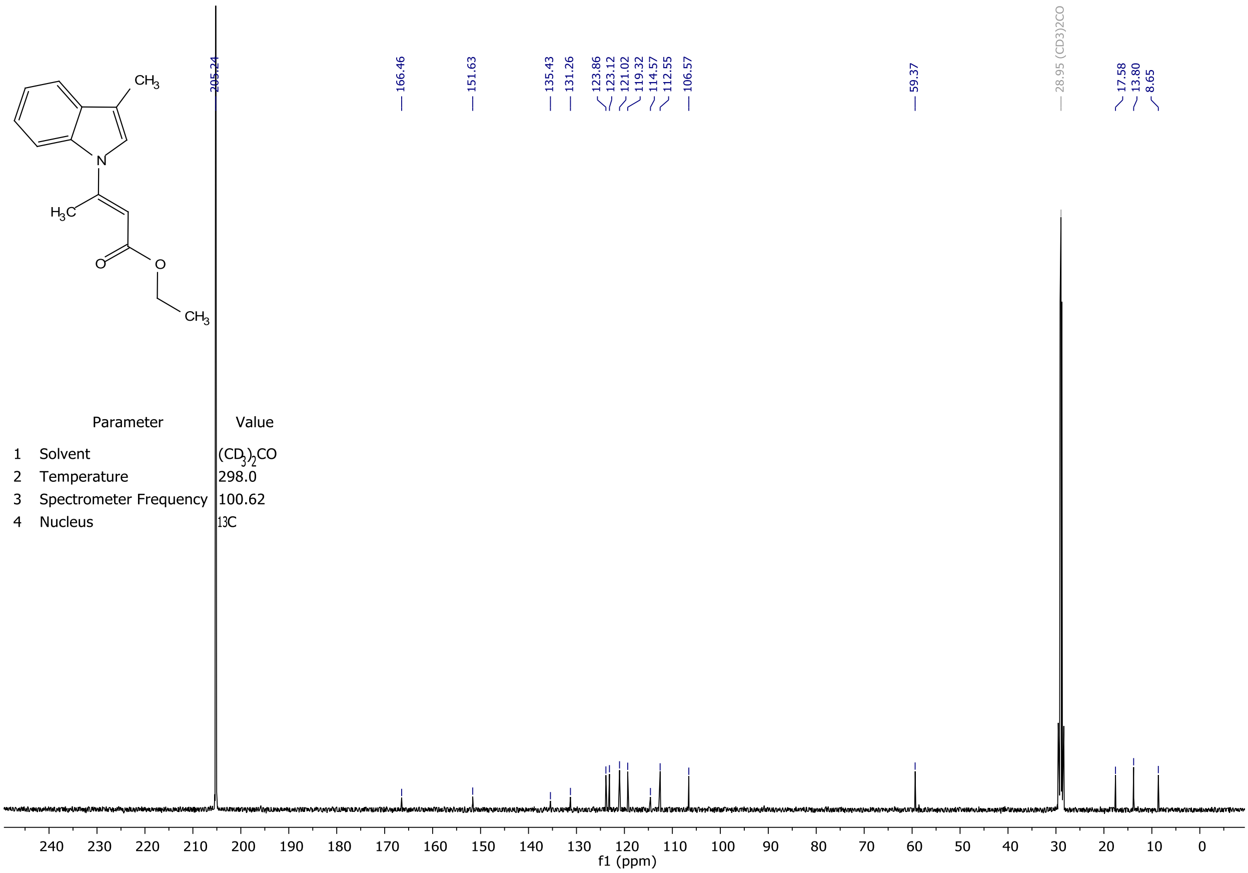

	Parameter	Value
1	Solvent	$(CD_3)_2CO$
2	Temperature	298.0
3	Spectrometer Frequency	100.62
4	Nucleus	13C

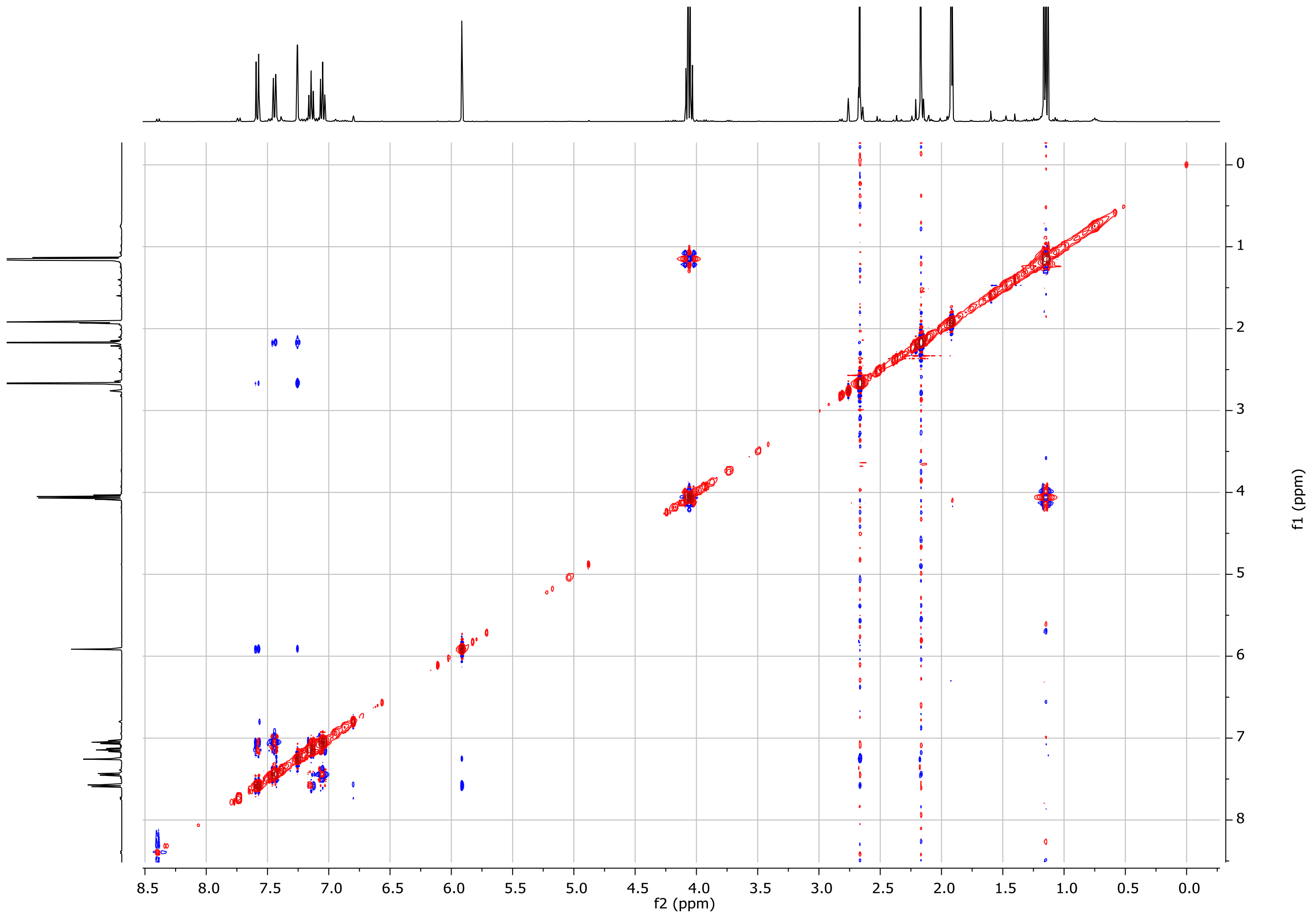

8.5
8.0
7.5
7.0
6.5
6.0
5.5
5.0
4.5
4.0
3.5
3.0
2.5
2.0
1.5
1.0
0.5
0.0
f2 (ppm)
0
1
2
3
4
5
6
7
8
f1 (ppm)

3-methyl-1-(2-methylcyclohex-1-en-1-yl)-1H-indole, 5n

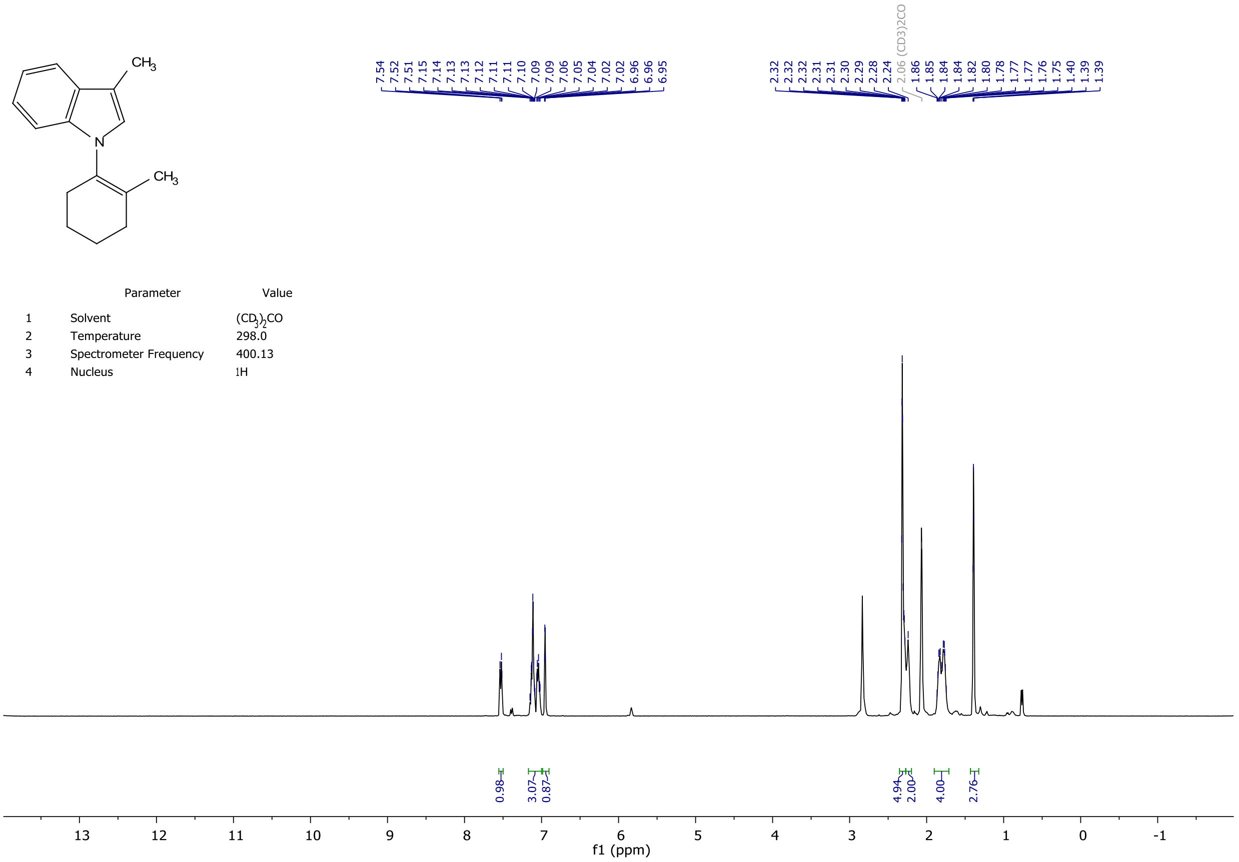

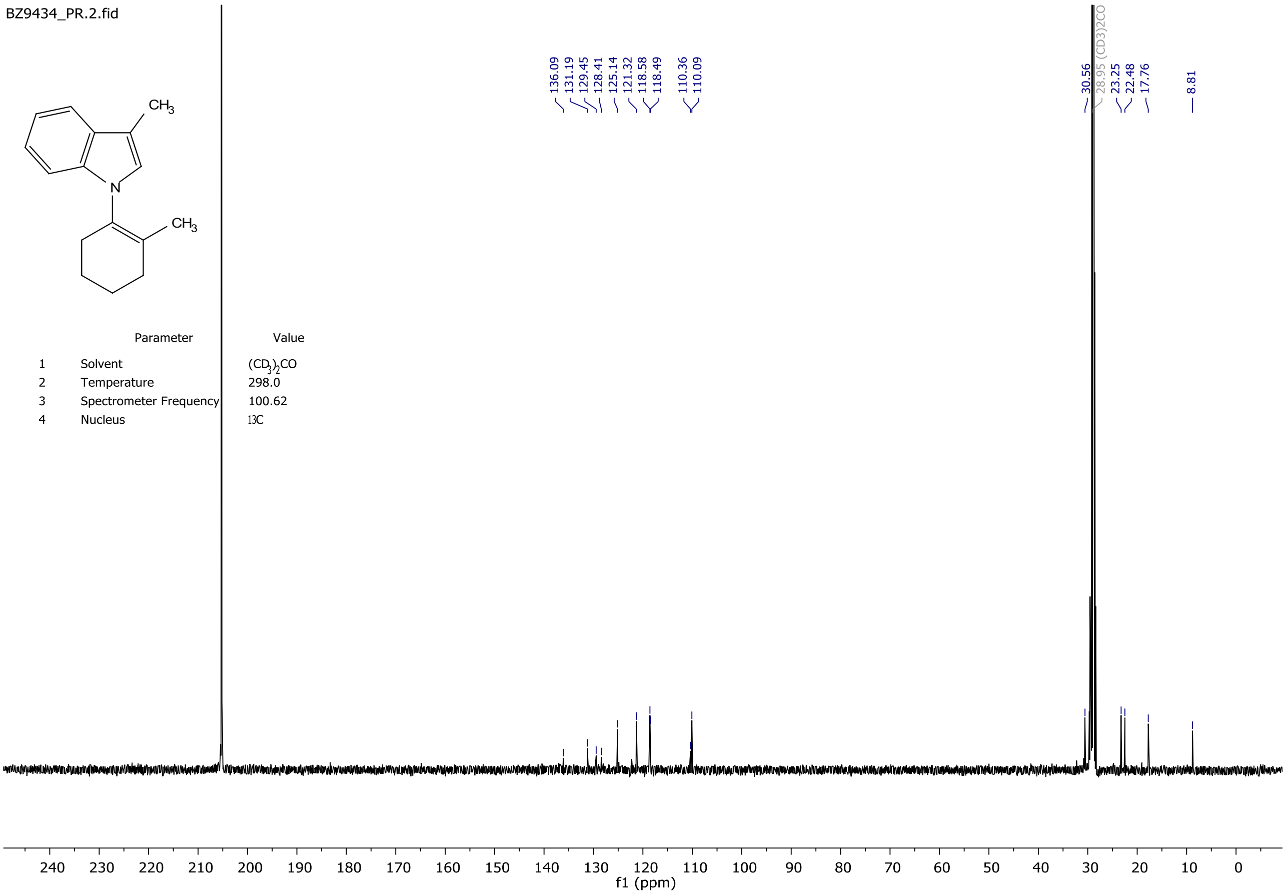

1-(2-allylcyclohex-1-en-1-yl)-3-methyl-1H-indole, 5o

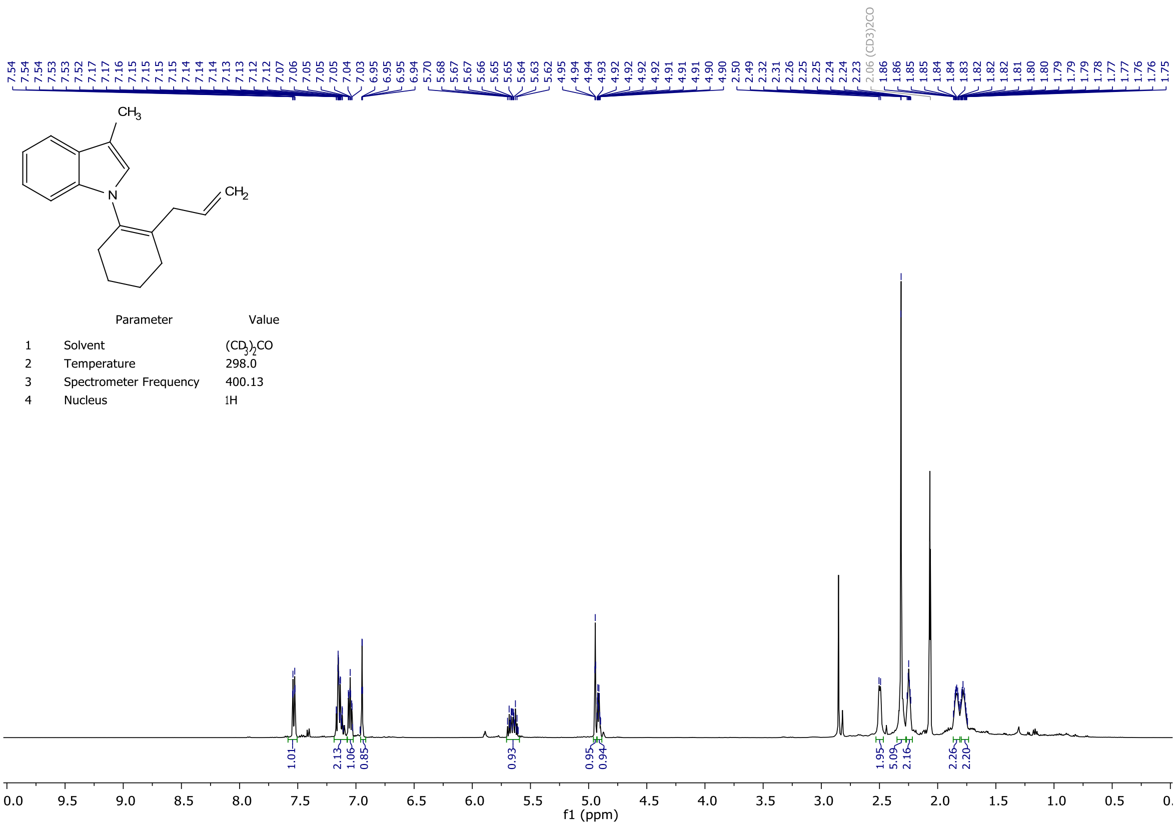

	Parameter	Value
1	Solvent	$(CD_3)_2CO$
2	Temperature	298.0
3	Spectrometer Frequency	400.13
4	Nucleus	1H

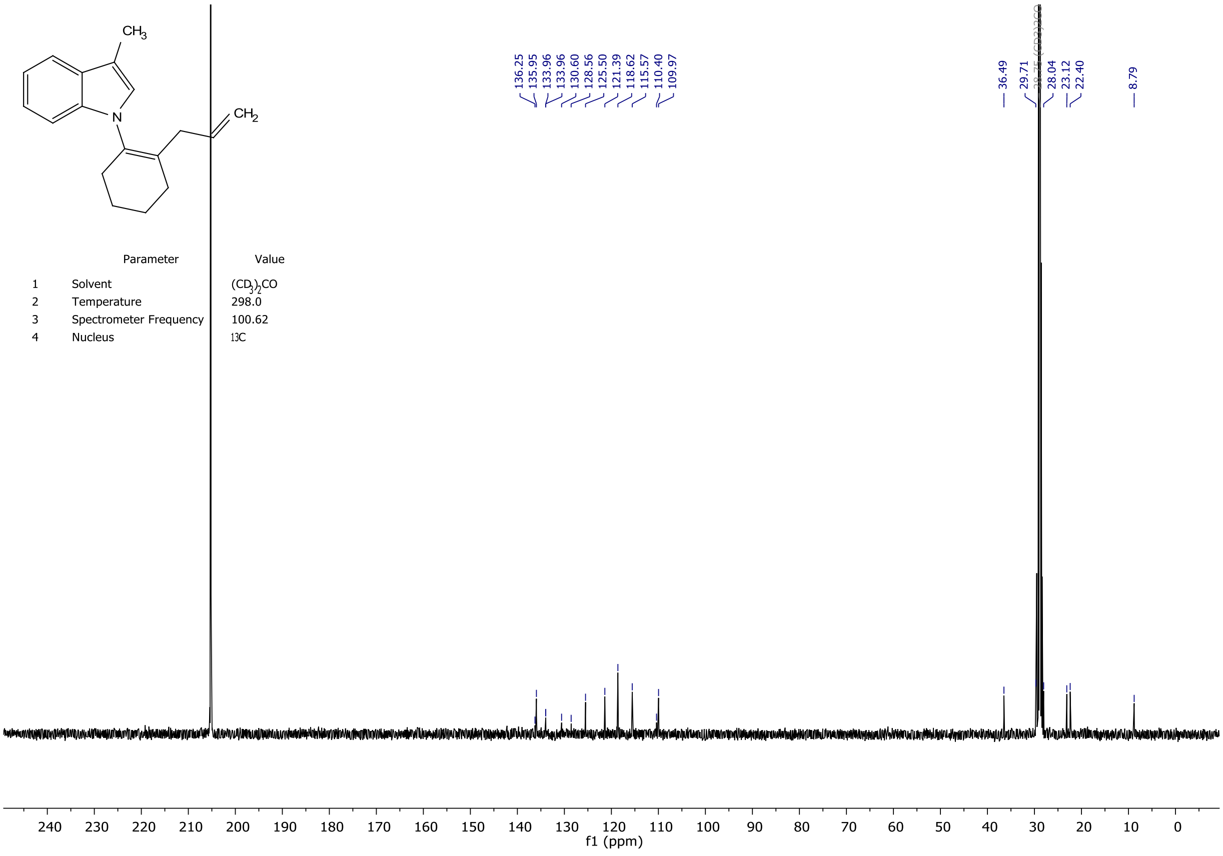

	Parameter	Value
1	Solvent	$(CD_3)_2CO$
2	Temperature	298.0
3	Spectrometer Frequency	100.62
4	Nucleus	13C

Reaction conducted using procedure B

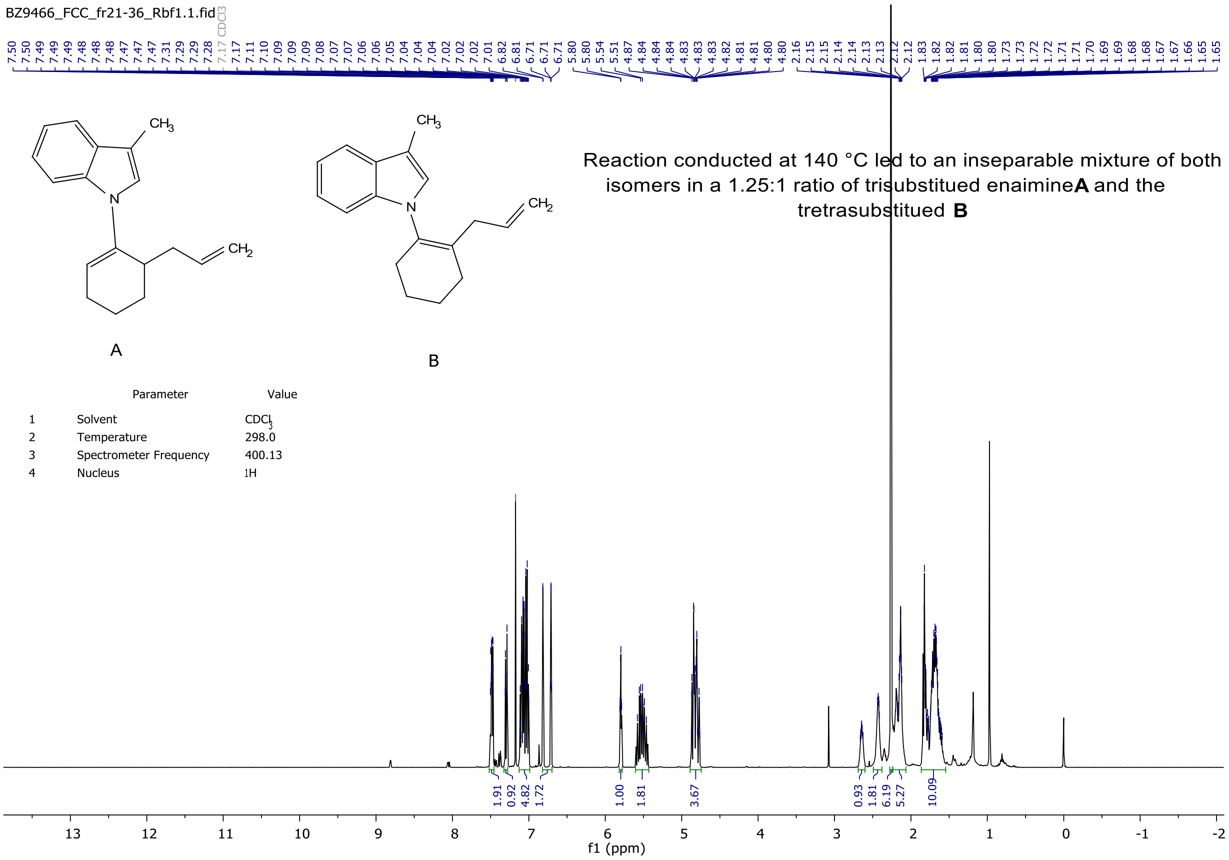

	Parameter	Value
1	Solvent	$CDCl_3$
2	Temperature	298.0
3	Spectrometer Frequency	400.13
4	Nucleus	1H

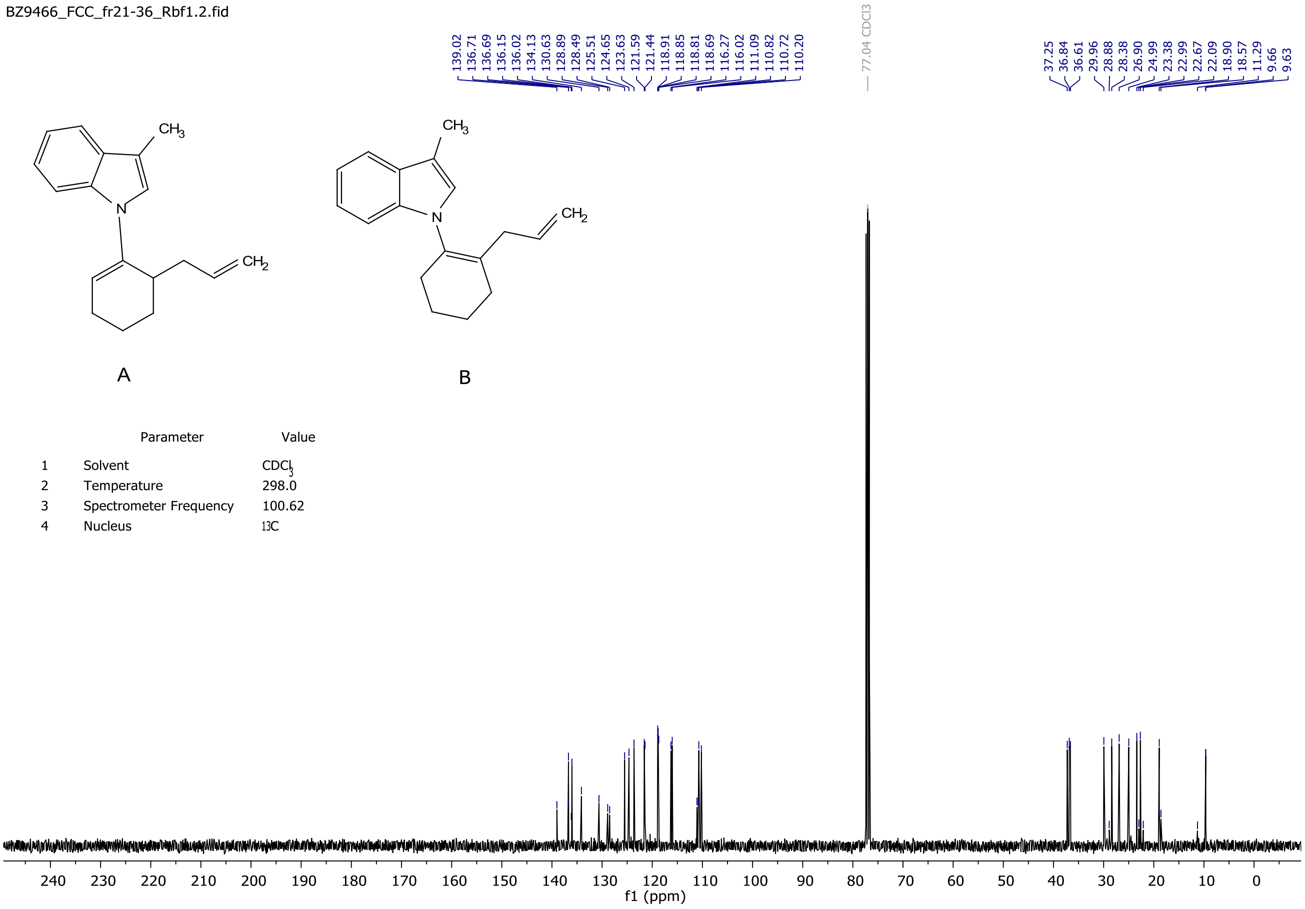

	Parameter	Value
1	Solvent	$CDCl_3$
2	Temperature	298.0
3	Spectrometer Frequency	100.62
4	Nucleus	13C

Inseparable mixture reacted using procedure C

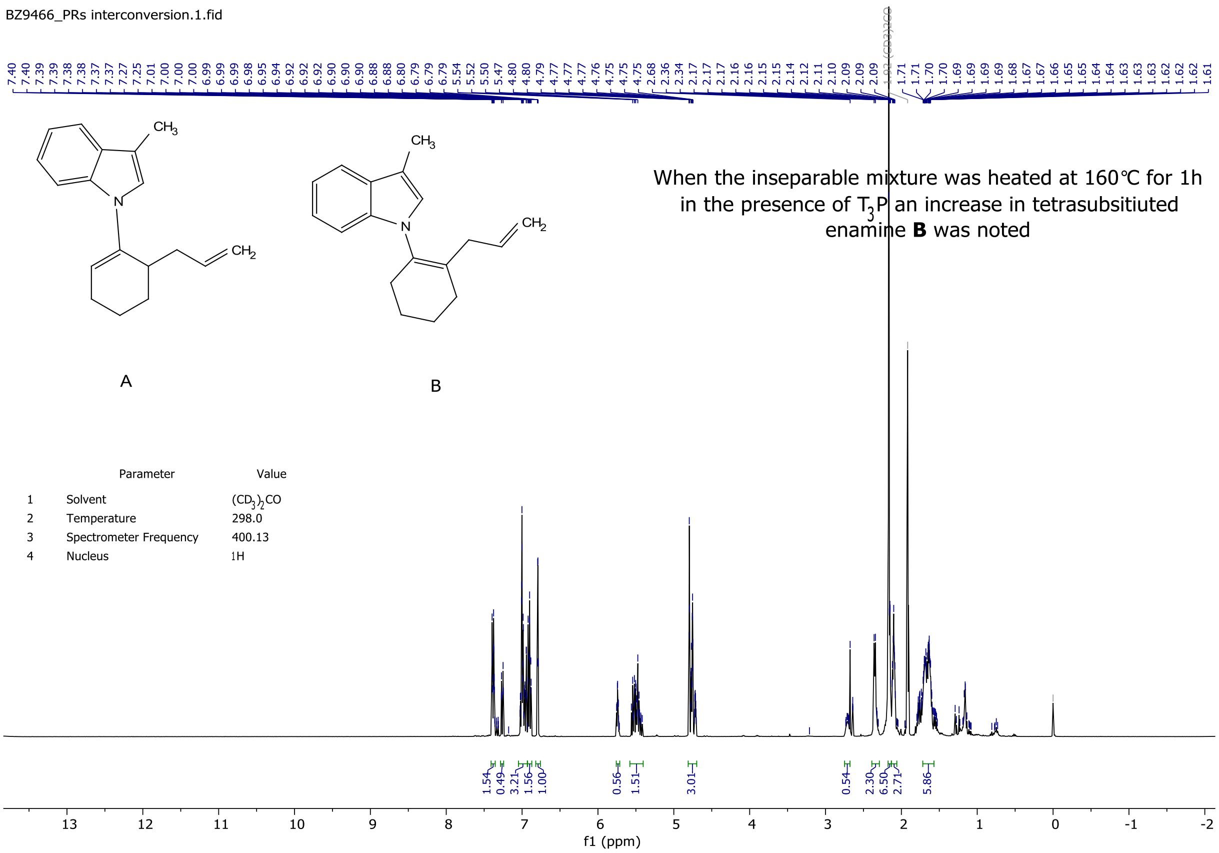

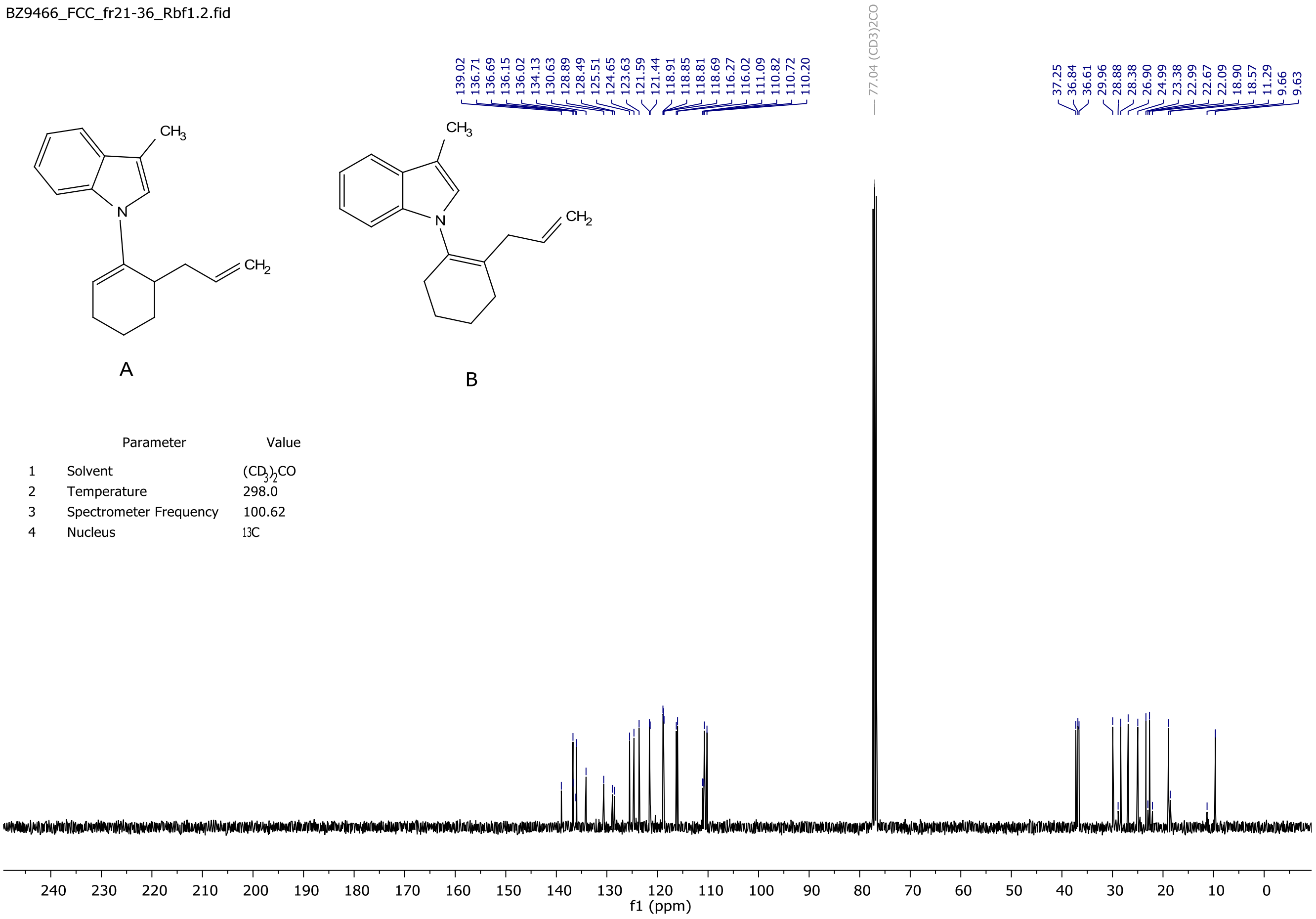

4-(3-methyl-1H-indol-1-yl)cyclohex-3-en-1-one, 5p

BZ9486_4setup_80C for 20 min_FCCfr25-35.1.fid

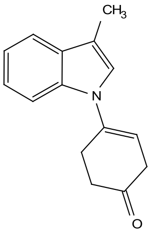

	Parameter	Value
1	Solvent	$(CD_3)_2CO$
2	Temperature	298.0
3	Spectrometer Frequency	400.13
4	Nucleus	1H

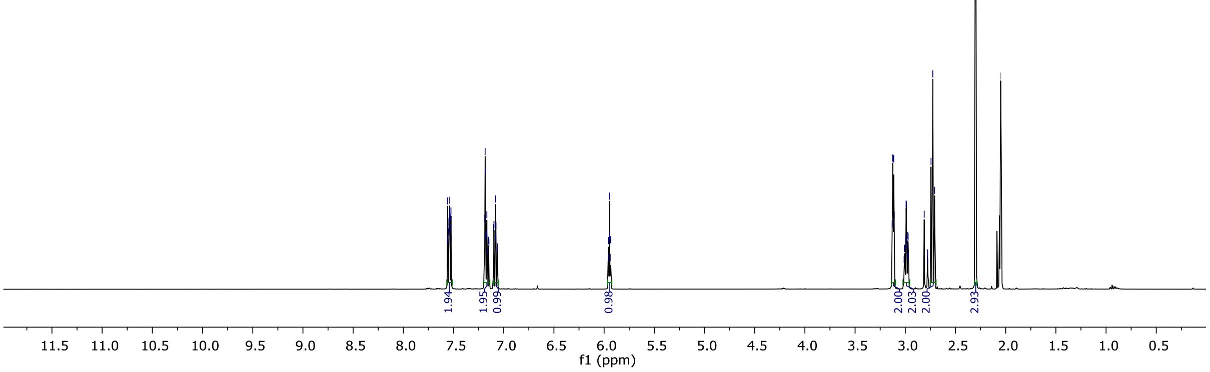

BZ9486_4setup_80C for 20 min_FCCfr25-35.2.fid

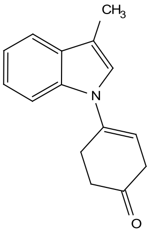

	Parameter	Value
1	Solvent	$(CD_3)_2CO$
2	Temperature	298.0
3	Spectrometer Frequency	100.62
4	Nucleus	13C

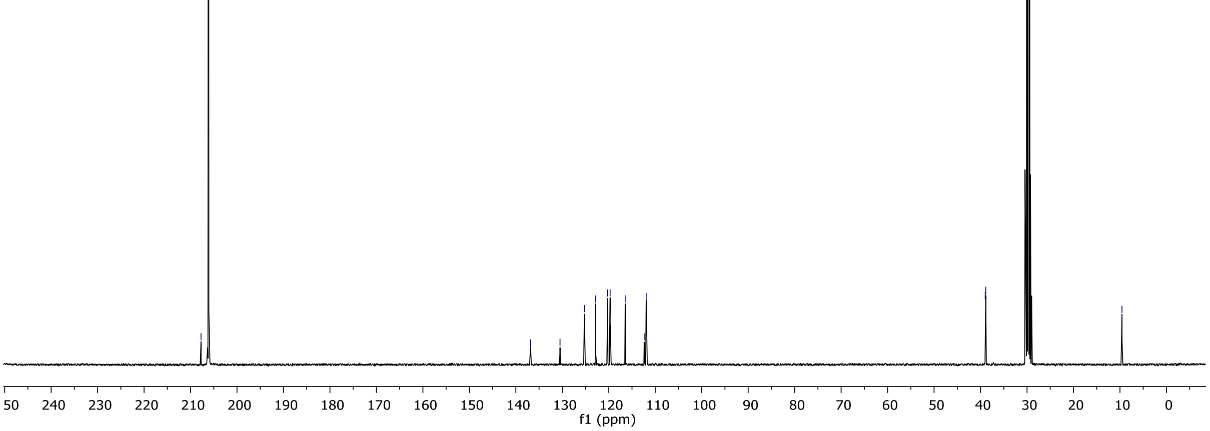

3-methyl-2-((3-methyl-1H-indol-1-yl)(phenyl)methyl)-1H-indole, 5q

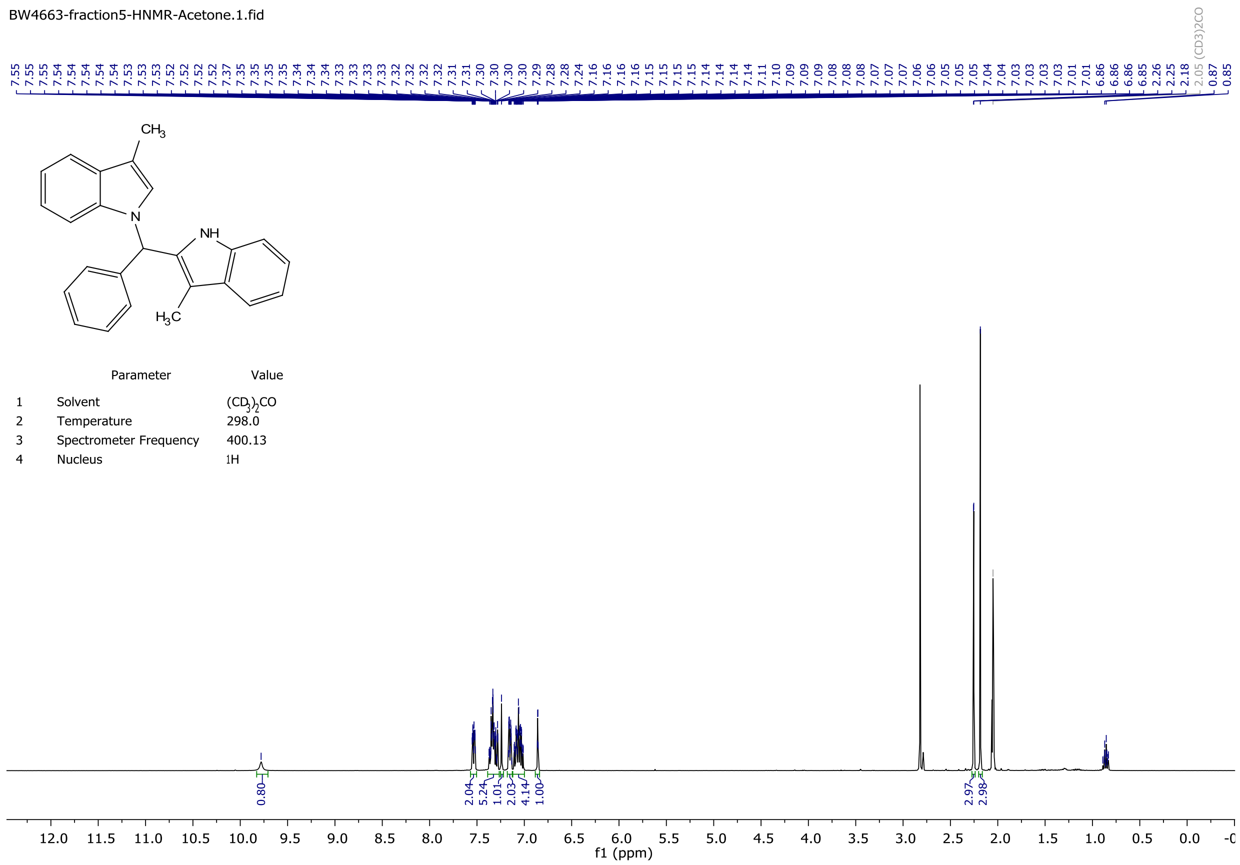

	Parameter	Value
1	Solvent	$(CD_3)_2CO$
2	Temperature	298.0
3	Spectrometer Frequency	400.13
4	Nucleus	1H

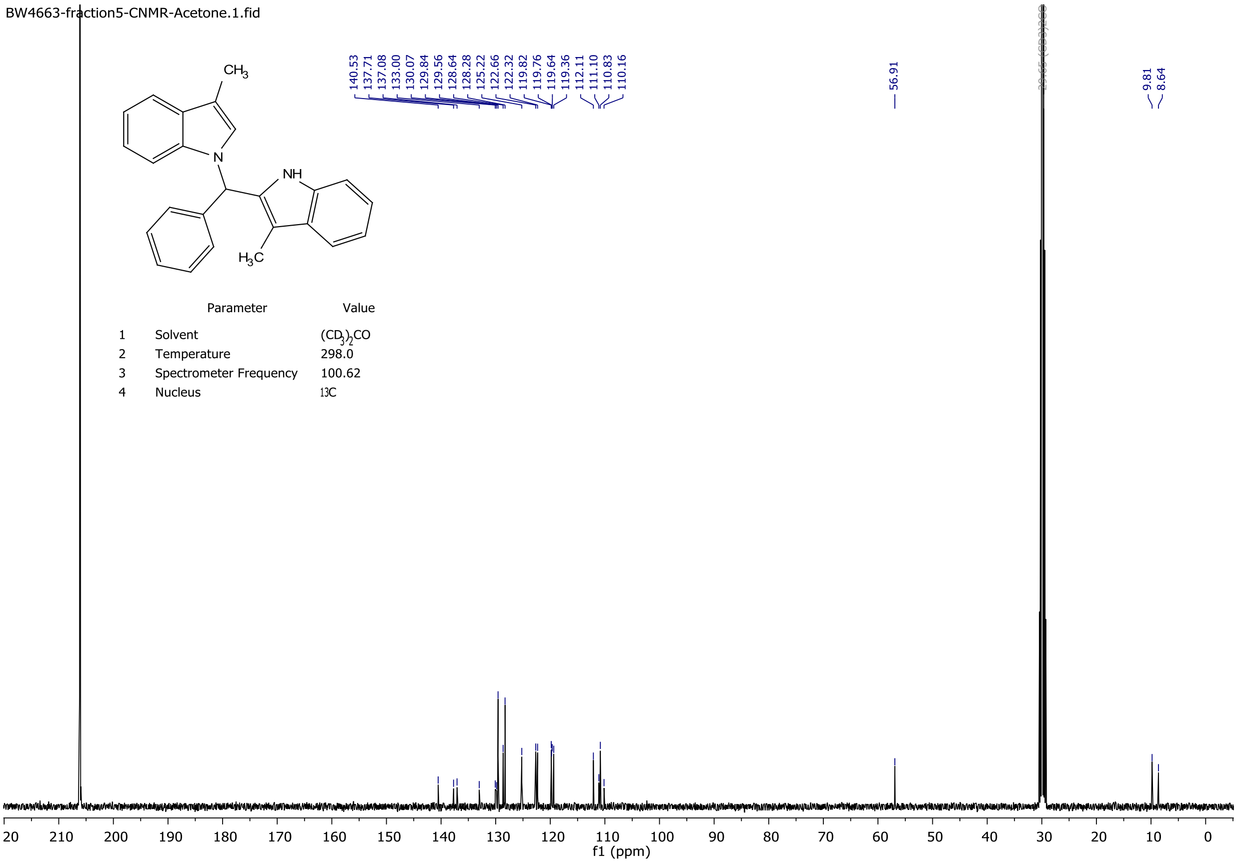

	Parameter	Value
1	Solvent	$(CD_3)_2CO$
2	Temperature	298.0
3	Spectrometer Frequency	100.62
4	Nucleus	13C

Large scale synthesis of 1-(Cyclohex-1-en-1-yl)-3-methyl-1H-indole, 3a

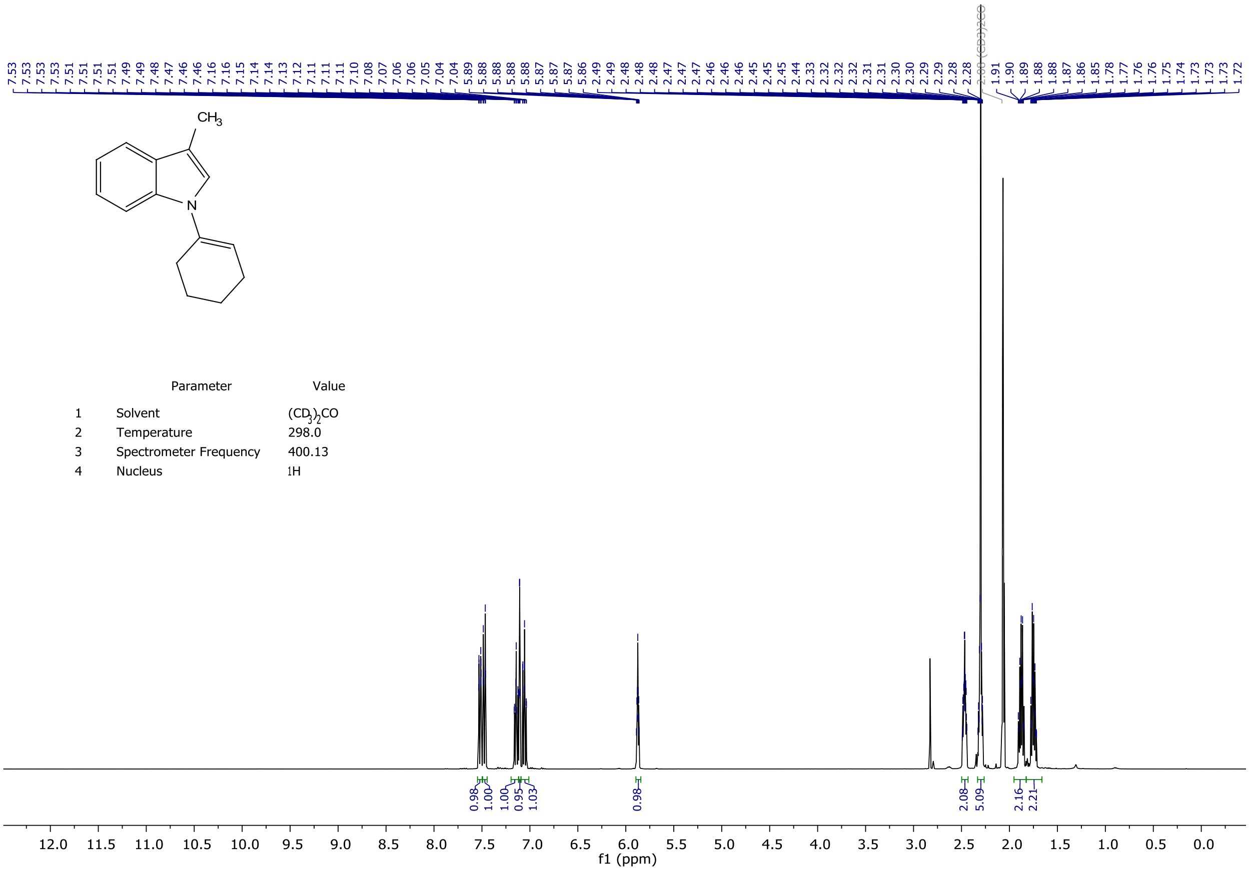

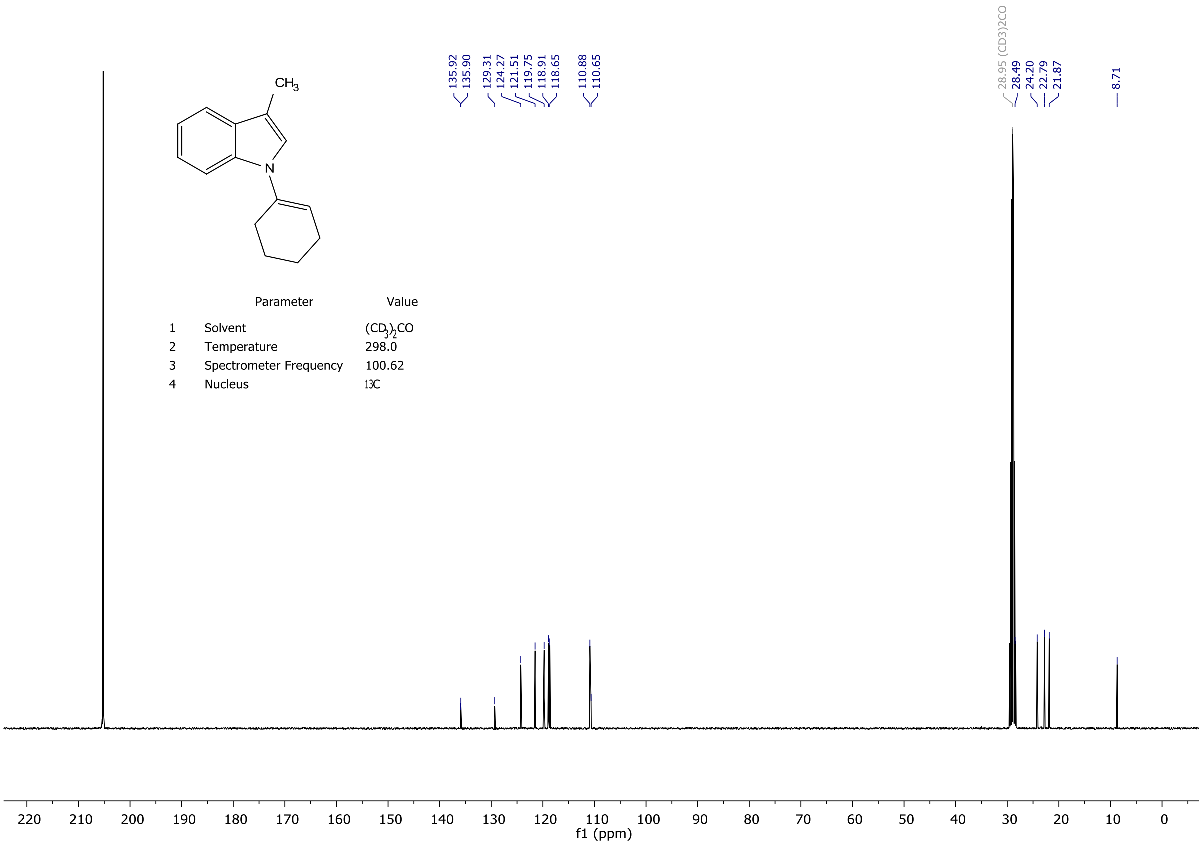

3β-acetoxy-17-(1H-benzimidazol-1-yl)-androsta-5,16-diene, 6 CAS 851895-79-9[6]

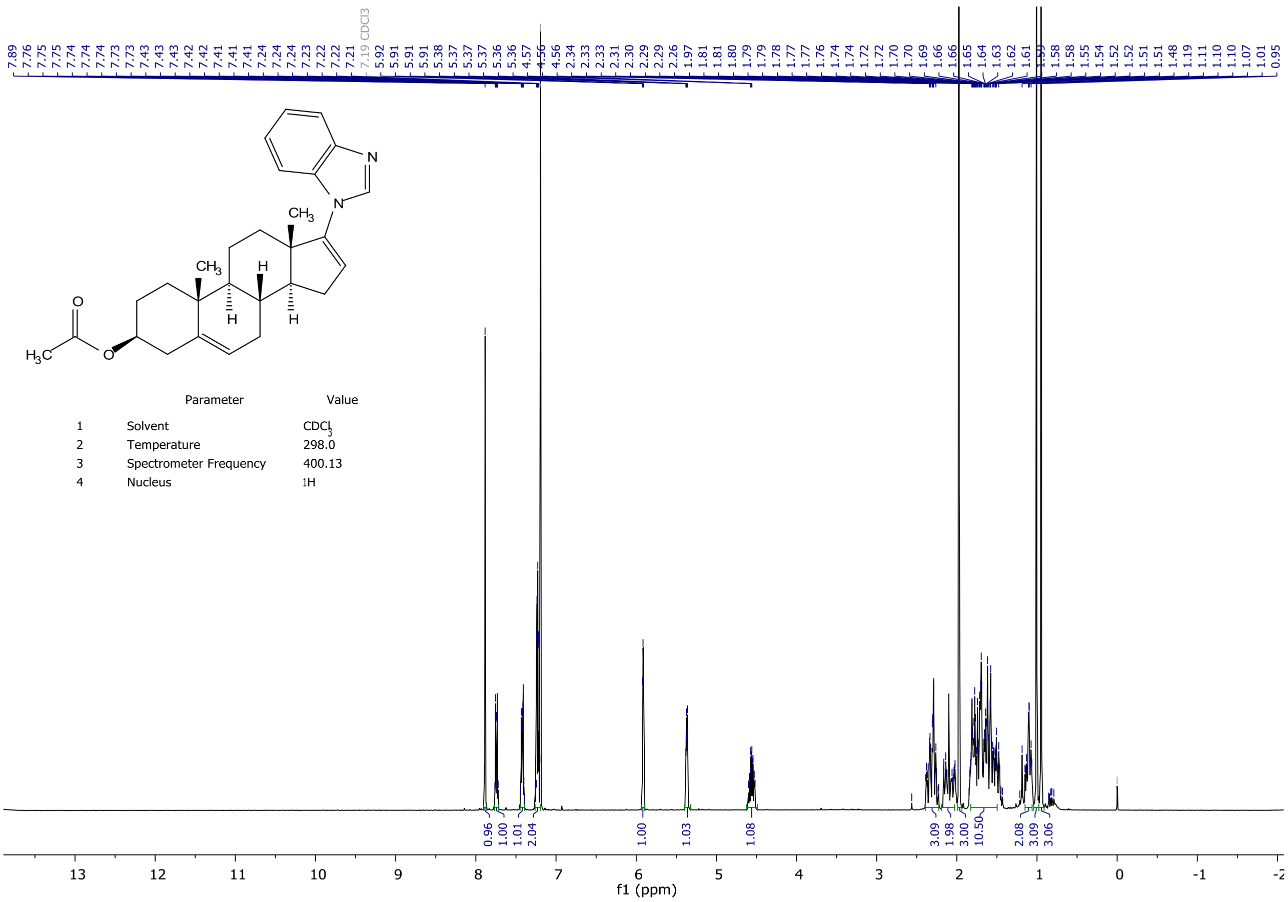

	Parameter	Value
1	Solvent	$CDCl_3$
2	Temperature	298.0
3	Spectrometer Frequency	400.13
4	Nucleus	1H

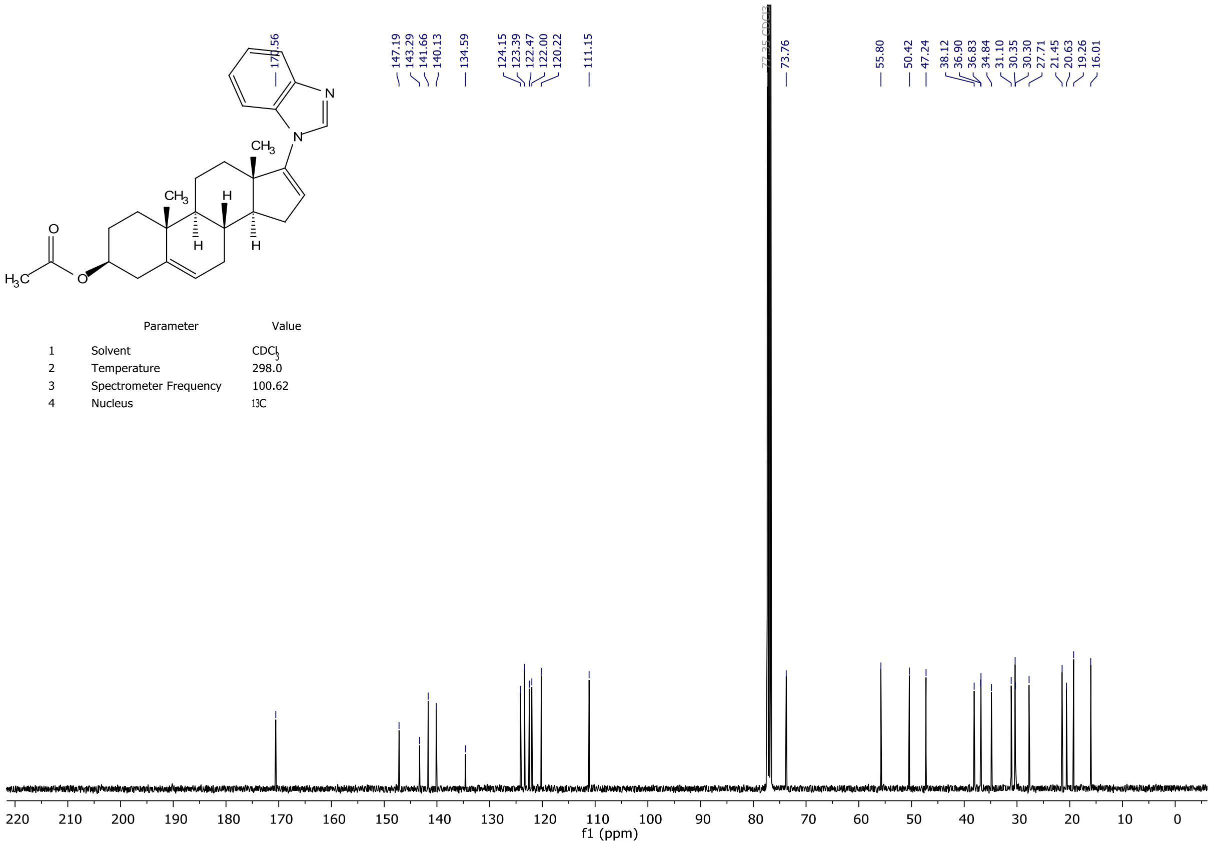

	Parameter	Value
1	Solvent	$CDCl_3$
2	Temperature	298.0
3	Spectrometer Frequency	100.62
4	Nucleus	13C

Synthesis of Galeterone, CAS 851983-85-2[6]

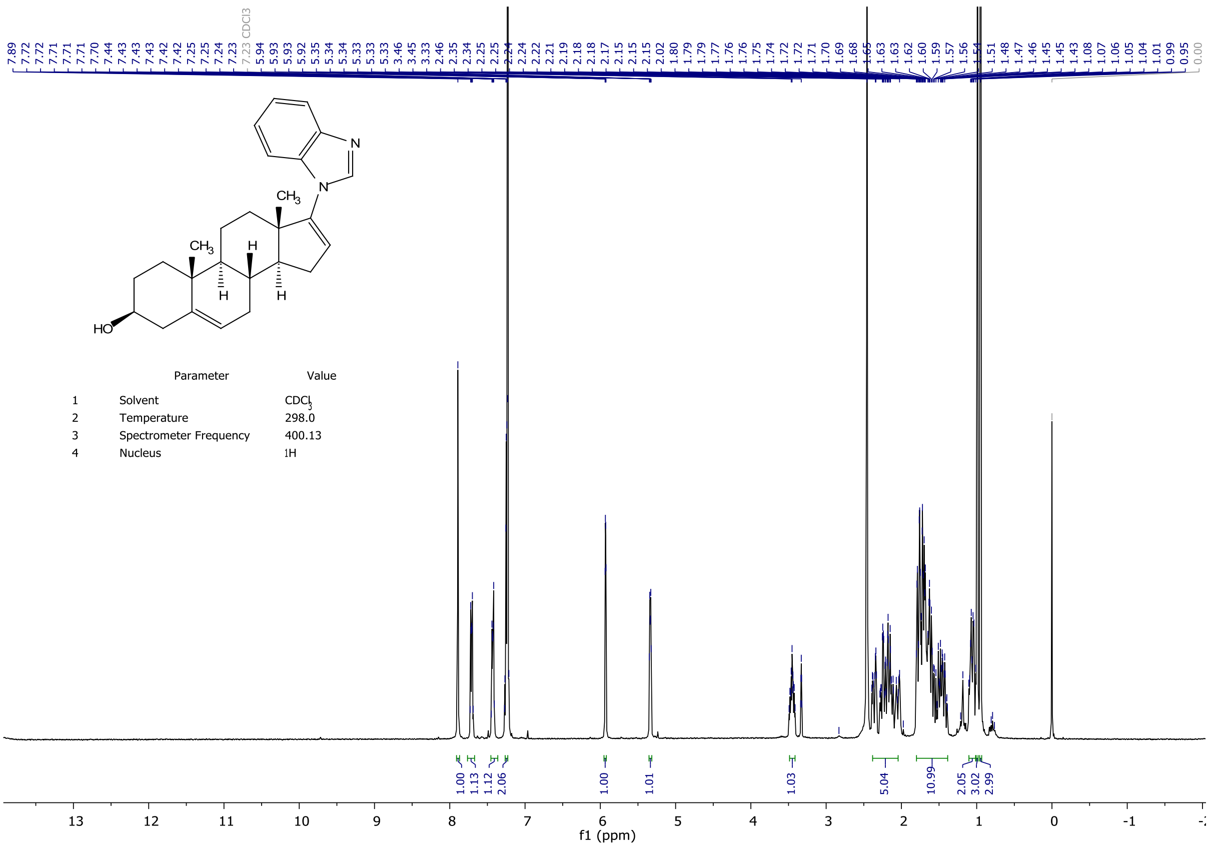

	Parameter	Value
1	Solvent	$CDCl_3$
2	Temperature	298.0
3	Spectrometer Frequency	400.13
4	Nucleus	1H

Galeterone-final.2.fid

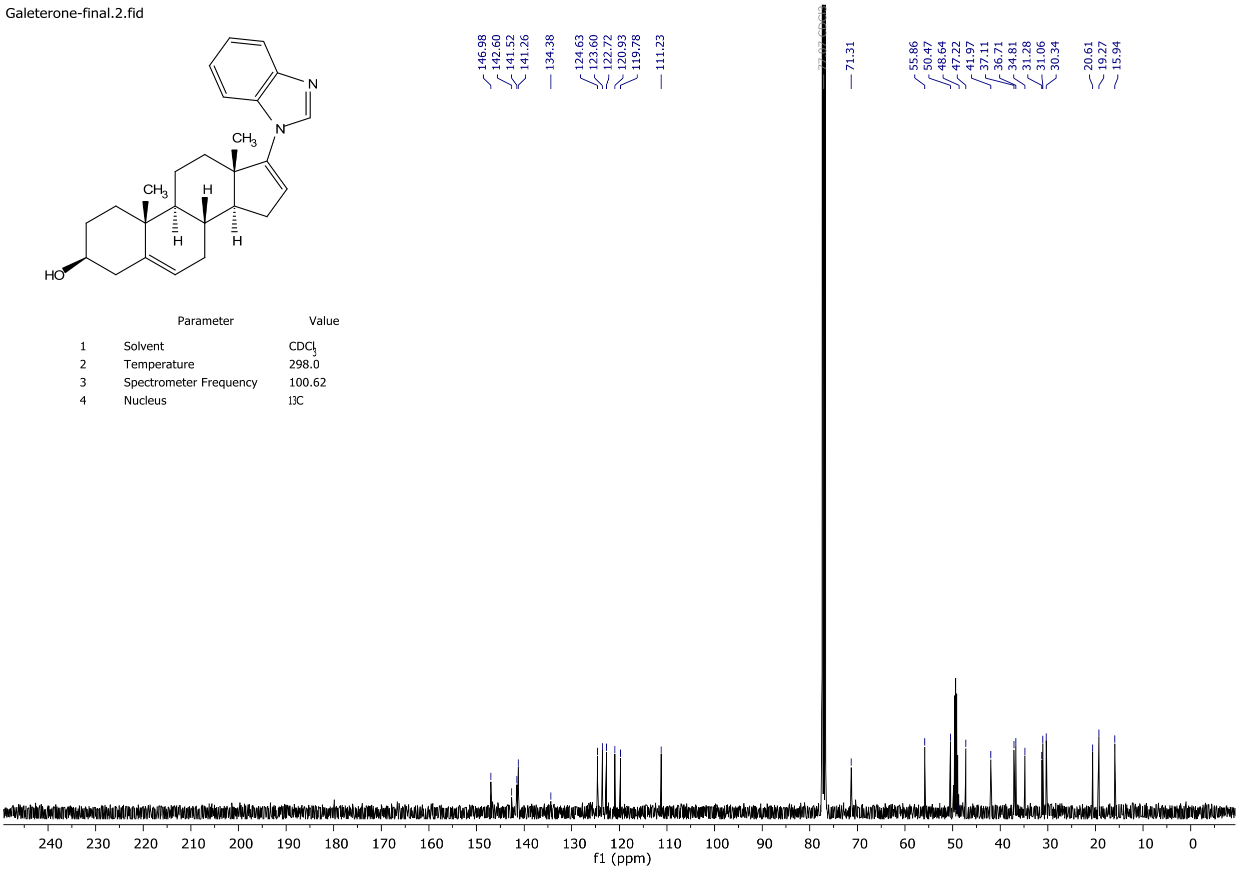

	Parameter	Value
1	Solvent	$CDCl_3$
2	Temperature	298.0
3	Spectrometer Frequency	100.62
4	Nucleus	13C

Benzyl 4-(1H-pyrazol-1-yl)-3,6-dihydropyridine-1(2H)-carboxylate, 9

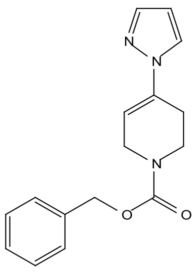

	Parameter	Value
1	Solvent	$CDCl_3$
2	Temperature	298.0
3	Spectrometer Frequency	400.13
4	Nucleus	1H

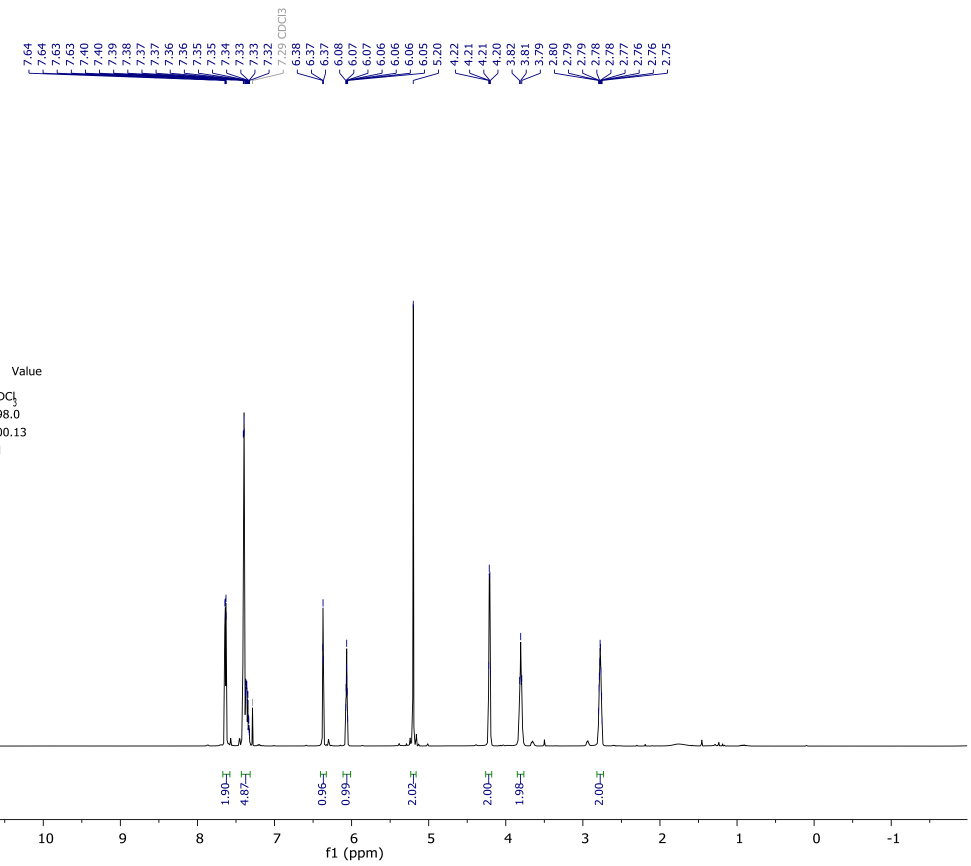

BX3387-3Setup 23-27.2.fid

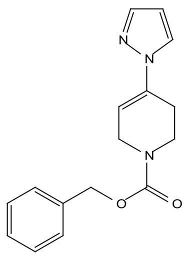

	Parameter	Value
1	Solvent	$CDCl_3$
2	Temperature	298.0
3	Spectrometer Frequency	100.62
4	Nucleus	13C

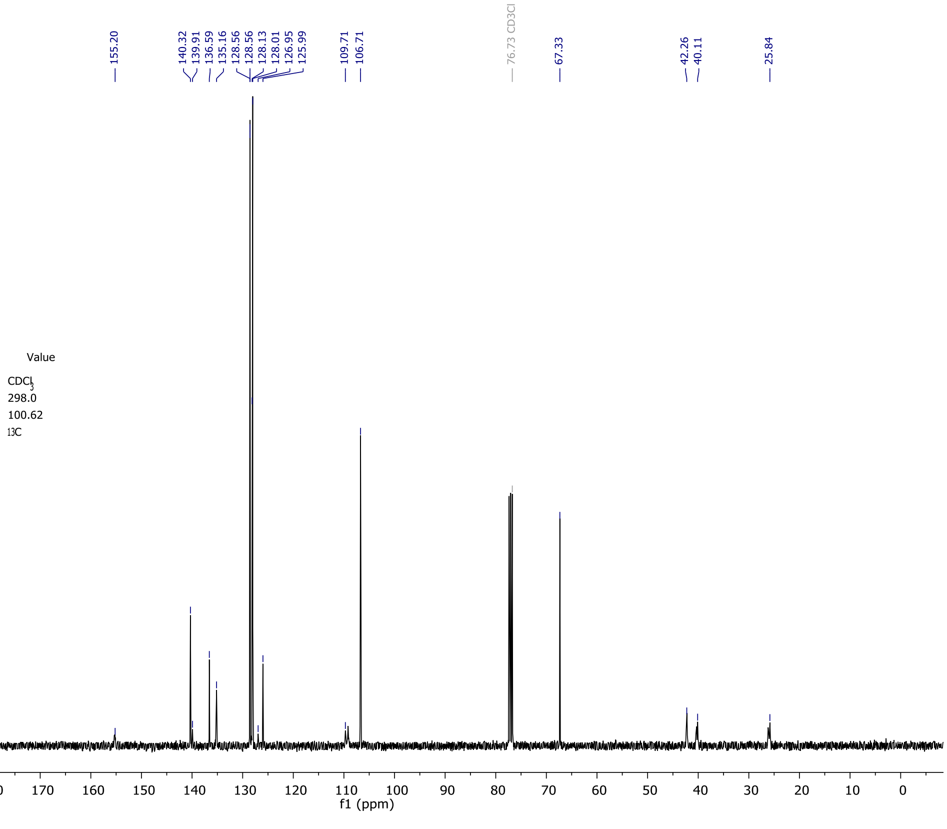

4-(1H-pyrazol-1-yl)piperidine, 8 CAS 762240-09-5[7]

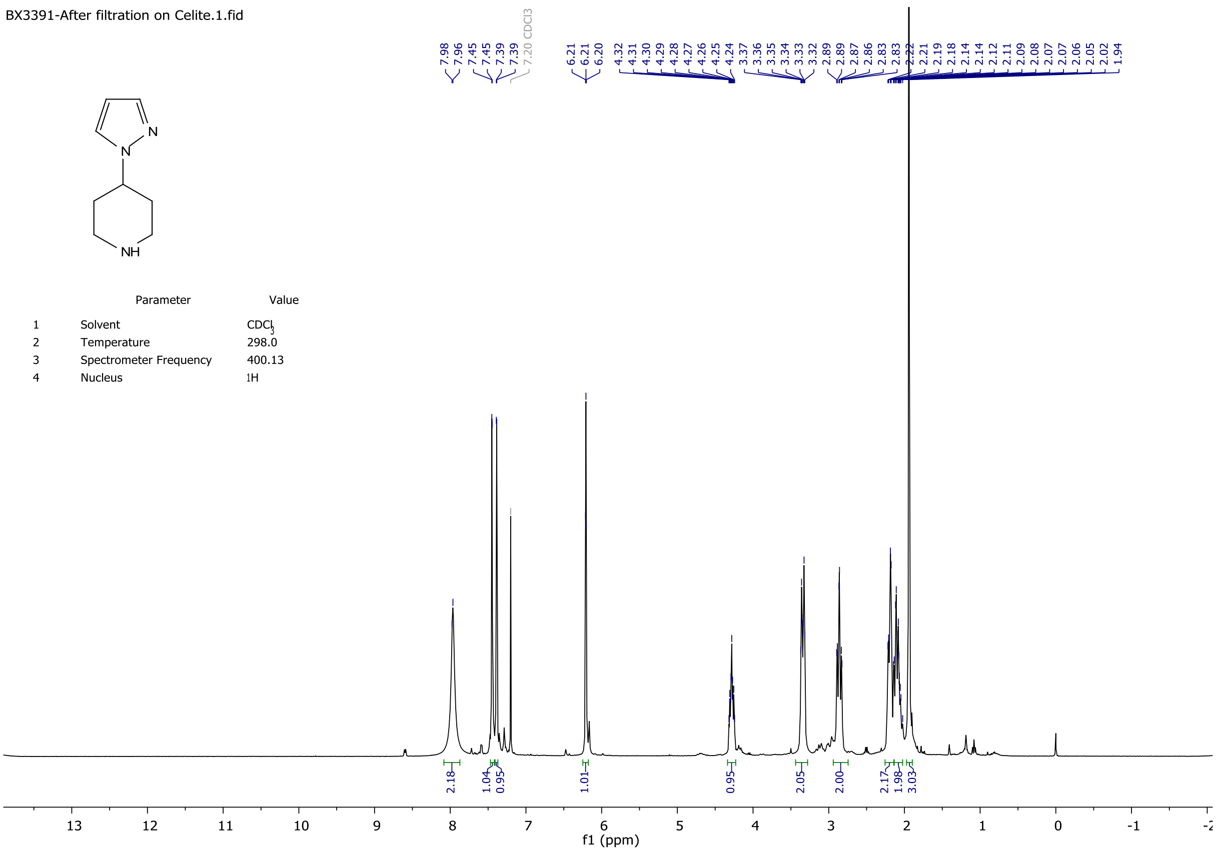

	Parameter	Value
1	Solvent	$CDCl_3$
2	Temperature	298.0
3	Spectrometer Frequency	400.13
4	Nucleus	1H

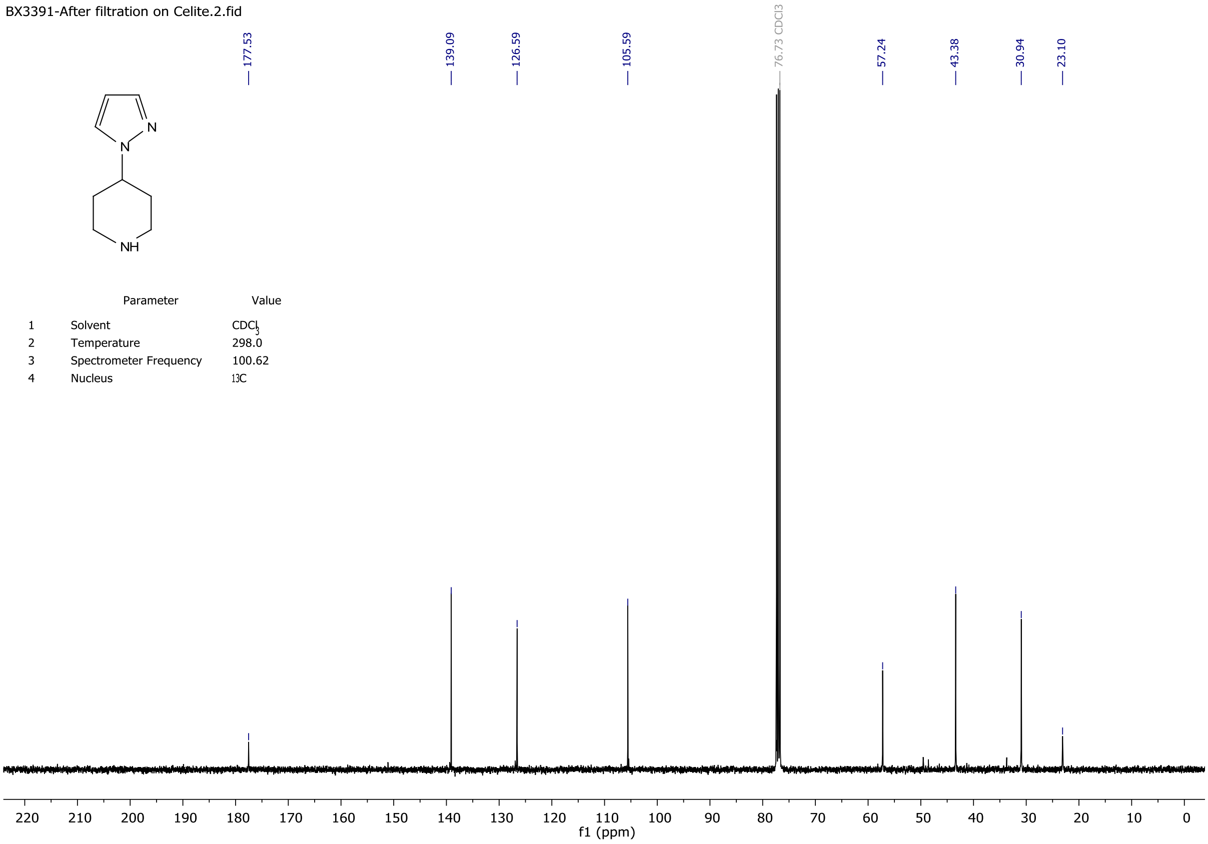

	Parameter	Value
1	Solvent	$CDCl_3$
2	Temperature	298.0
3	Spectrometer Frequency	100.62
4	Nucleus	13C

1-Cyclohexyl-3-methyl-1H-indole, 11 CAS 1037739-68-6[8]

lo5142chr_PROTON_01

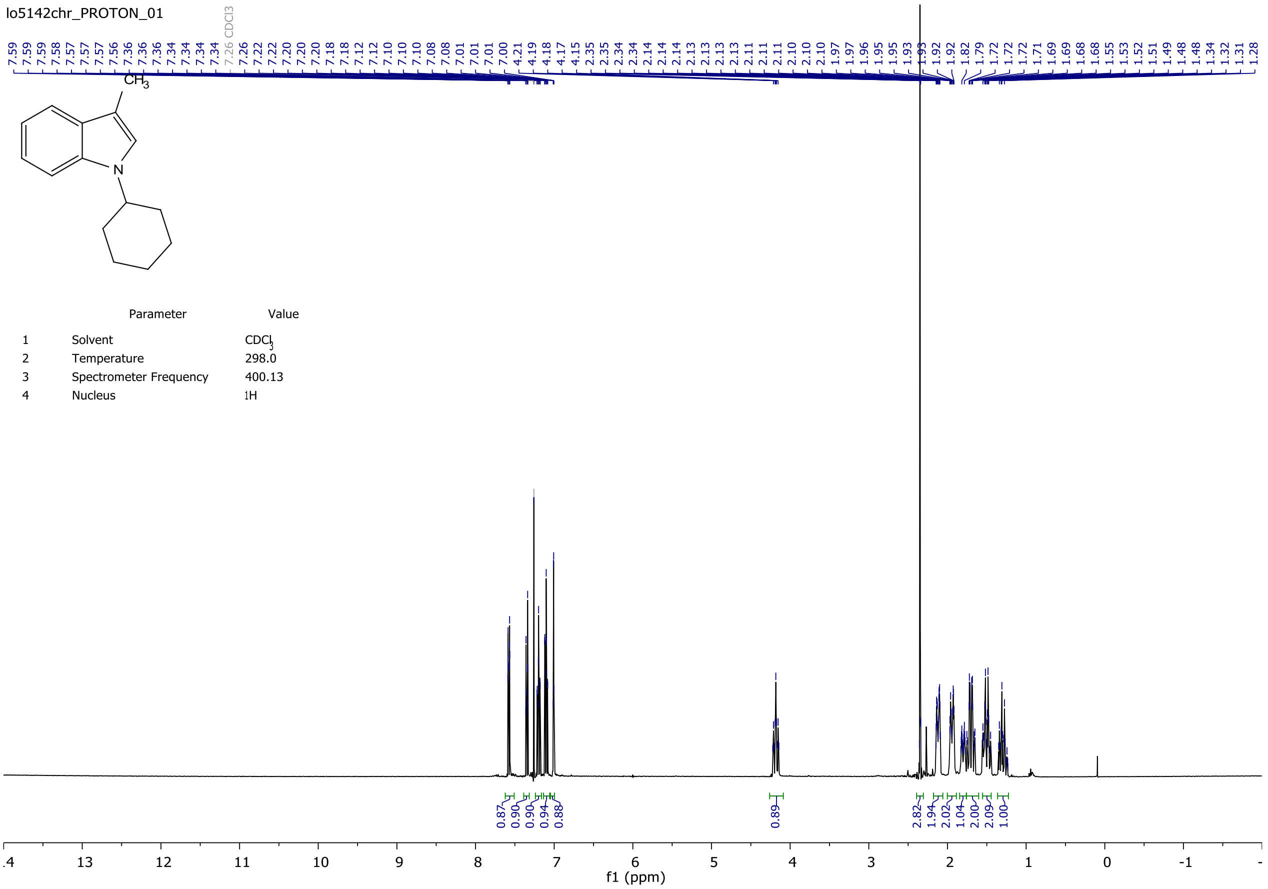

	Parameter	Value
1	Solvent	$CDCl_3$
2	Temperature	298.0
3	Spectrometer Frequency	400.13
4	Nucleus	1H

lo5142chr_CARBON_01

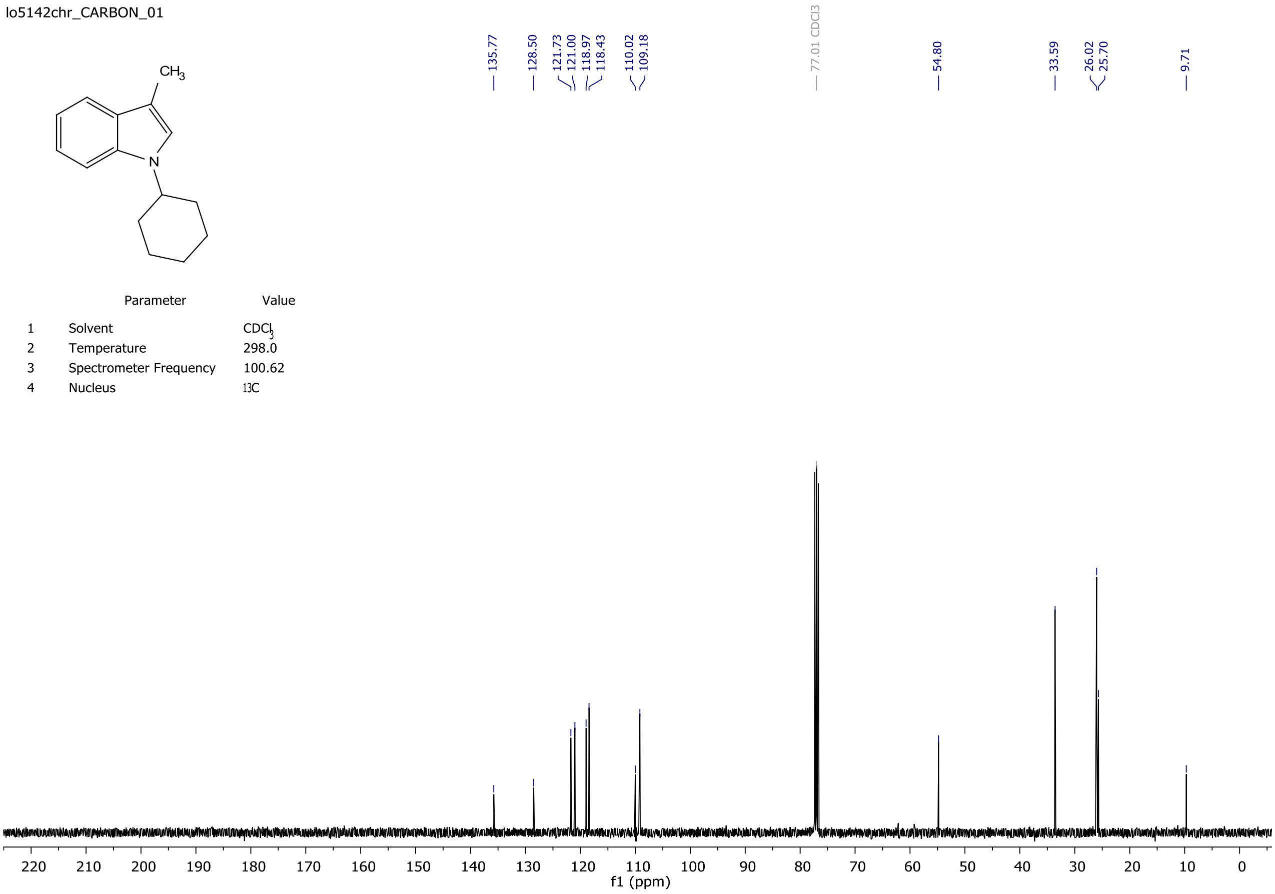

	Parameter	Value
1	Solvent	$CDCl_3$
2	Temperature	298.0
3	Spectrometer Frequency	100.62
4	Nucleus	13C

References

(1) Katritzky, A. R.; Maimait, R.; Xu, Y.-J.; Gyoung, Y. S. Synthesis of N-Cycloalkenylazoles. *J. Org. Chem.* **2002**, *67* (23), 8230–8233. https://doi.org/10.1021/jo020336m.

(2) Wang, X.; Wang, Q.; Xue, Y.; Sun, K.; Wu, L.; Zhang, B.; Li, R.; Chemcomm, /; Communication, C. An Organoselenium-Catalyzed N 1-and N 2-Selective Aza-Wacker Reaction of Alkenes with Benzotriazoles *Chem. Commun.* **2020**, 4436. https://doi.org/10.1039/d0cc01079k.

(3) Meng, X.; Li, X.; Chen, W.; Zhang, Y.; Wang, W.; Chen, J.; Song, J.; Feng, H.; Chen, B. Facile One-Pot Synthesis of N-Alkylated Benzimidazole and Benzotriazole from Carbonyl Compounds *J. Heterocyclic Chem.* **2014**, *51*, 349. https://doi.org/10.1002/jhet.1616.

(4) Kilic, H.; Bayindir, S.; Erdogan, E.; Agopcan Cinar, S.; Konuklar, F. A. S.; Bali, S. K.; Saracoglu, N.; Aviyente, V. Bismuth Nitrate-Promoted Disproportionative Condensation of Indoles with Cyclohexanone: A New-Type Azafulvenium Reactivity of Indole. *New J. Chem.* **2017**, *41* (18), 9674–9687. https://doi.org/10.1039/c7nj01987d.

(5) Liao, Q.; Wang, Y.; Zhang, L.; Xi, C. A General Copper-Catalyzed Coupling of Azoles with Vinyl Bromides. *J. Org. Chem.* **2009**, *74* (16), 6371–6373. https://doi.org/10.1021/jo901105r.

(6) Handratta, V. D.; Vasaitis, T. S.; Njar, V. C. O.; Gediya, L. K.; Kataria, R.; Chopra, P.; Newman, D.; Farquhar, R.; Guo, Z.; Qiu, Y.; Brodie, A. M. H. Novel C-17-Heteroaryl Steroidal CYP17 Inhibitors/Antiandrogens: Synthesis, in Vitro Biological Activity, Pharmacokinetics, and Antitumor Activity in the LAPC4 Human Prostate Cancer Xenograft Model. *J. Med. Chem.* **2005**. 48, (8), 2972–2984. https://doi.org/10.1021/jm040202w.

(7) Fussell, S. J.; Luan, A.; Peach, P.; Scotney, G. A Three-Step Synthesis of 4-(4-Iodo-1H-Pyrazol-1-Yl)Piperidine, a Key Intermediate in the Synthesis of Crizotinib. *Tetrahedron Lett* **2012**, *53* (8), 948–951. https://doi.org/10.1016/j.tetlet.2011.12.044.

(8) Schirok, H. Microwave-Assisted Synthesis of N-Sec- and N-Tert-Alkylated Indoles. *Synthesis* **2008**, *9*, 1404–1414. https://doi.org/10.1055/s-2008-1067005.
